# Non-Graphene Two-Dimensional Materials: Structure–Property–Application Relationships from a Curated Data Analysis

**DOI:** 10.1021/acsmaterialsau.6c00190

**Published:** 2026-09-29

**Authors:** Magesh Ganesan, Bhuvaneshwari S. Baranwal, Suparna Roy, Qiongqiong Angela Zhou

**Affiliations:** † ACS International India, No 23, Baner One, Near Baner-Pancard Club Road, Pune 411045, Maharashtra, India; ‡ 2645 CAS, a division of the American Chemical Society, 2540 Olentangy River Road, Columbus, Ohio 43202, United States

**Keywords:** 2D materials, two-dimensional materials, MXenes, applications of 2D materials, 2D chalcogenides, Xenes, bibliometric analysis, patent landscape analysis, electrocatalysis, van der Waals materials

## Abstract

Two-dimensional (2D) materials beyond graphene have emerged as a diverse and rapidly expanding family of atomically thin structures with unique properties derived from quantum confinement, reduced dielectric screening, and high surface-to-volume ratios. This comprehensive data-driven analysis examines over 66 k publications from the CAS Content Collection (2016–2026), revealing the evolution, compositional diversity, and application landscape of nongraphene 2D materials. Publication volume increased nearly 10-fold during this period, with journal articles comprising 81% and patents 19%, reflecting strong academic momentum alongside commercial translation. Systematic classification identifies 12 major material families: MXenes (transition metal carbides/nitrides) dominate with metallic conductivity and tunable surface chemistry; Xenes (elemental 2D materials including phosphorene, silicene, borophene) offer semiconducting-to-metallic behavior; transition metal dichalcogenides (TMDs) provide tunable bandgaps for electronics and photonics; metal-free nitrides (h-BN, g-C_3_N_4_) serve specialized roles in dielectrics and photocatalysis; and emerging classes, including 2D perovskites, metal halides, organic frameworks (MOFs/COFs), layered double hydroxides, and clay minerals, expand functional diversity. Application mapping reveals clear structure–property–application relationships: redox-active systems excel in energy storage and catalysis; semiconductors with tunable bandgaps dominate electronics and photonics; layered structures provide barrier properties for coatings; specialized electronic configurations enable magnetic and photocatalytic functions. Growth trends indicate a transition from conventional systems toward compositionally engineered variants like Janus TMDs, ternary LDHs, mixed-halide perovskites, and functionalized MXenes. This highlights the field’s evolution toward application-driven materials design. This analysis provides strategic insights for rational material selection and identifies underexplored opportunities spanning flexible electronics, sustainable catalysis, and advanced energy technologies.

## Graphical abstract

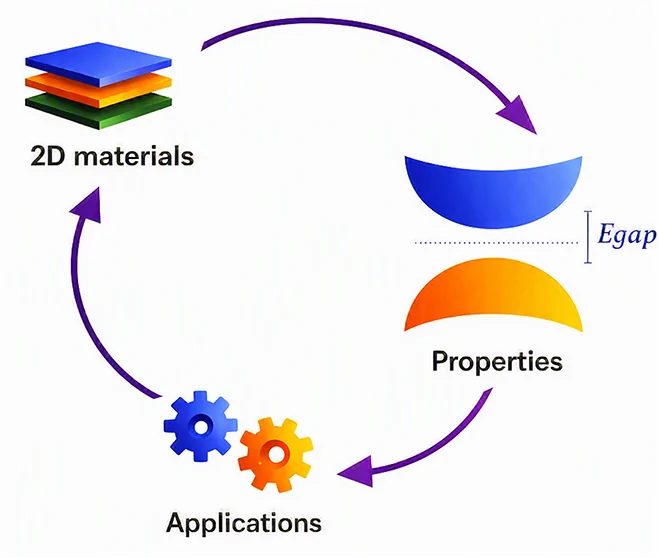

## 1 Introduction

Material size plays a crucial role in determining its physical, chemical, and electronic properties. Reducing dimensions often leads to size-dependent phenomena, such as changes in electronic behavior and an increased influence of surface atoms, which are absent in bulk systems. This fundamental understanding has driven the evolution of nanotechnology, where precise control at the nanoscale enables the tuning of material properties and enhances surface activity. Among low-dimensional nanomaterials, two-dimensional (2D) materials, defined by atomic-scale thickness and extended lateral dimensions occupy a unique position. Their properties are governed by quantum confinement, reduced dielectric screening, and inherently high surface-to-volume ratios.^1^ These characteristics have positioned 2D materials at the forefront of contemporary research in modern science and technology.

The isolation of graphene marked a pivotal milestone in the development of 2D materials, establishing the feasibility of stable atomically thin crystals and stimulating widespread interest in layered systems.^2^ Over the past two decades graphene has been intensively investigated, leading to a comprehensive understanding of its exceptional mechanical strength, high carrier mobility, thermal conductivity, and limitations associated with the absence of an intrinsic bandgap. While several nongraphene 2D materials are now well established, the broader nongraphene 2D-materials landscape remains chemically diverse and unevenly explored.^3^ This diverse class includes metallic and conductive MXenes, characterized by their rich surface chemistry and high electrical conductivity, have attracted significant attention for energy storage applications, including supercapacitors and various metal-ion batteries (Li-, Na-, and K-ion).^4^ 2D perovskites, featuring quantum-confined metal–halide layers separated by spacer cations, demonstrate tunable optoelectronic properties and improved stability, enabling their use in photovoltaics, light-emitting diodes, and photodetectors.^5^ Semiconducting transition metal dichalcogenides (TMDs) such as molybdenum disulfide (MoS_2_), tungsten disulfide (WS_2_), and tungsten diselenide (WSe_2_), possess intrinsic and tunable bandgaps, making them promising candidates for field-effect transistors, photodetectors, and optoelectronic devices.^6^ Insulating hexagonal boron nitride (h-BN), with its excellent dielectric properties and atomically smooth surface, is widely used as a substrate and gate dielectric in high-performance electronic devices.^7^ In addition, a growing range of layered oxides is being explored for applications spanning electronics, catalysis, and energy conversion.^8^ Collectively, these materials offer greater chemical diversity and functional versatility than graphene, enabling properties and device functionalities that are difficult to achieve with graphene alone.

As the family of nongraphene 2D materials continues to expand, the volume and diversity of reported experimental data have grown significantly, encompassing synthesis methods, physical properties, and device performance. However, much of this information remains dispersed across individual studies, limiting the ability to identify broader trends and establish quantitative structure–property relationships. In response, data-driven analysis provides a powerful complement to traditional narrative reviews by enabling systematic comparisons across material systems, synthesis approaches, properties, and application areas. Accordingly, in this report we adopt a data-driven approach to examine 2D materials beyond graphene, analyzing data from the CAS Content Collection^9^ to uncover trends, evaluate performance benchmarks, and generate insights that can guide future research and application-focused design.

Building on this framework, this study presents a comprehensive analysis of the CAS Content Collection on nongraphene 2D materials published from 2016 to March 2026, covering over 66 thousand documents. Publications containing 2D materials were identified using CAS IP Finder,^10^ powered by STN and CAS data indexed from these publications such as concepts,^11^ substances,^12^ and bibliometric information were extracted for analysis (see Methods in Supporting Information). We first examine publication trends, followed by classification and subclassification of material groups based on relevant parameters. Leveraging the robust indexing system of the CAS Content Collection, we further analyze the substances within each category and correlate them with their applications and associated properties. This approach highlights growth pattern within specific material classes and reveals emerging research patterns. Overall, the study provides a data-driven overview of nongraphene 2D materials, offering actionable insights to support future research and application-oriented design.

## 2 Analysis of CAS Data on 2D Materials

### 2.1 Bibliometric Analysis

To elucidate trends in the evolution of nongraphene two-dimensional (2D) material research, over 66 thousand documents from the CAS Content Collection spanning 2016–2026 were systematically analyzed (Figure 1A). Temporal assessment reveals a pronounced increase in journal publications on nongraphene 2D materials, with output rising nearly 10-fold between 2016 and 2025. This substantial growth reflects intensified scientific interest and expanding research activity in this domain. Concurrently, the steady rise in patent publications highlights a strengthening linkage between fundamental research and its translational and commercial potential. Overall, journal articles constitute the majority of the data set (81%), while patents account for the remaining 19%, underscoring both strong academic momentum and parallel progression toward technological development in this field. Figure 1B presents the distribution of journal and patent publications across the 10 countries and regions with the highest number of publications. China leads in terms of total number of publications followed by United States, India, South Korea, Germany, Iran, and Japan. While the United States, India, Germany, Iran, and Japan make substantial contributions to the scientific literature, their comparatively lower patent activity suggests a weaker translation of fundamental research into intellectual property. In contrast, South Korea and China demonstrates a notably high patent-to-journal publication ratio, indicative of a highly application-oriented innovation ecosystem.

**Figure 1:**
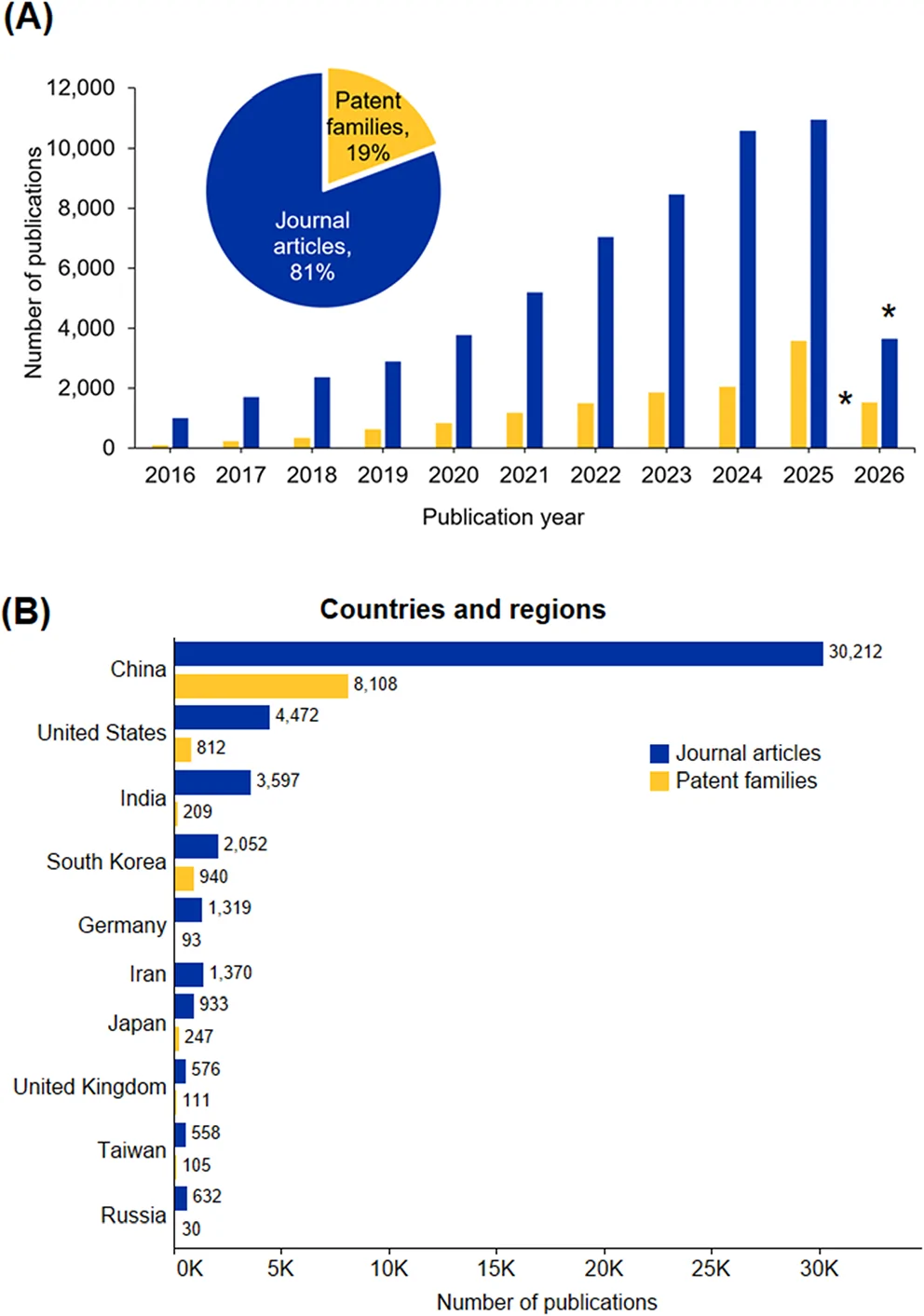
(A) Yearly trends of patent families and journal articles published on nongraphene 2D materials research for the period 2016–2026. *Data was extracted from CAS Content Collection and for 2026 data is partial and encompasses January to March.  (B) Number of journal and patent publications from the 10 countries and regions with the highest number of publications.

### 2.2 Material Map

Publications containing specific classes of 2D materials or applications were identified based on indexed concepts,^11^ substances,^12^ and keywords in the extracted data. The systematic organization of 2D materials presented in Figure 2 encompasses over 66 thousand publications, structured across 12 major material families using chemistry-driven hierarchical taxonomies.

**Figure 2:**
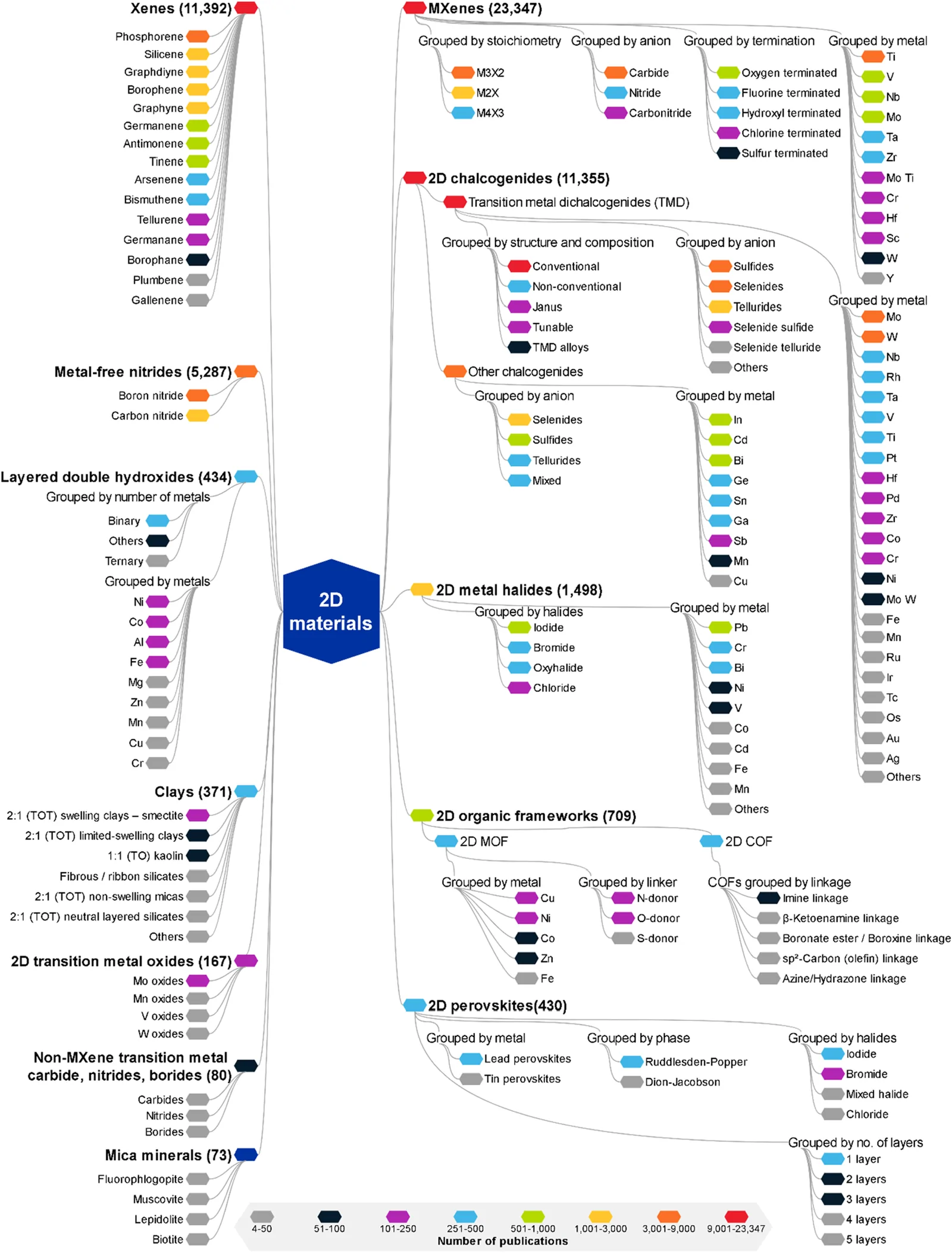
Map on 2D materials based on publications presenting the 2D materials classes and their number of publications. Some classes are further subdivided based on different parameters and their number of publications is presented. Data extracted from the CAS Content Collection and includes journal articles and patent publications between 2016 to March 2026.

MXenes constitute the most studied class, categorized according to stoichiometry (e.g., M_3_X_2_, M_2_X, M_4_X_3_), anionic composition (carbides, nitrides, carbonitrides), surface termination chemistries (O, F, OH, Cl, S), and metal center identity (e.g., V, Nb, Mo, Ti, Ta, Zr). Xenes represent a major family of elemental 2D materials derived from group IV and V elements, including phosphorene (black phosphorus), silicene, graphdiyne, borophene, germanene, arsenene, antimonene, and other metal analogues. Closely following, the 2D chalcogenide family comprises both transition metal dichalcogenides (TMDs) and their nontransition metal counterparts. TMDs are systematically classified by structural and compositional variants (conventional, nonconventional, Janus, tunable, and alloyed forms), chalcogen type (sulfides, selenides, tellurides, and mixed systems), and metal composition, including Mo, W, Nb, Re, Rh, Ir, V, Ti, and Pt. Metal-free nitrides, predominantly encompassing boron nitride and carbon nitride systems, form another significant category. Metal halides represent an additional category of layered materials with growing interest. Organic 2D frameworks are subdivided into metal–organic frameworks (MOFs) and covalent organic frameworks (COFs). MOFs are classified based on metal nodes (e.g., Cu, Ni, Co, Zn, Fe) and linker donor atoms, whereas COFs are categorized by linkage chemistry. Layered double hydroxides are organized based on cation composition, followed by 2D perovskites, which are classified according to metal identity, phase, layer number, and halide composition. Transition metal oxides and non-MXene carbides, borides, and nitrides constitute emerging yet comparatively underexplored categories of 2D materials. Naturally occurring layered materials such as clays and mica minerals complete the taxonomy. Collectively, this classification establishes a comprehensive and scalable framework for systematically mapping the rapidly expanding landscape of 2D materials.

### 2.3 Application Landscape

To elucidate the application landscape of 2D materials, we analyzed CAS data and mapped their distribution across diverse technological domains. In Figure 3, each cell shows publication counts for an application–2D material class pair. Each column (one material class) has its own independent color scale, enabling quick comparison of application focus within that class. The resulting heatmap reveals distinct, application-specific specialization patterns across 12 families of 2D materials spanning 15 technological domains.

**Figure 3:**
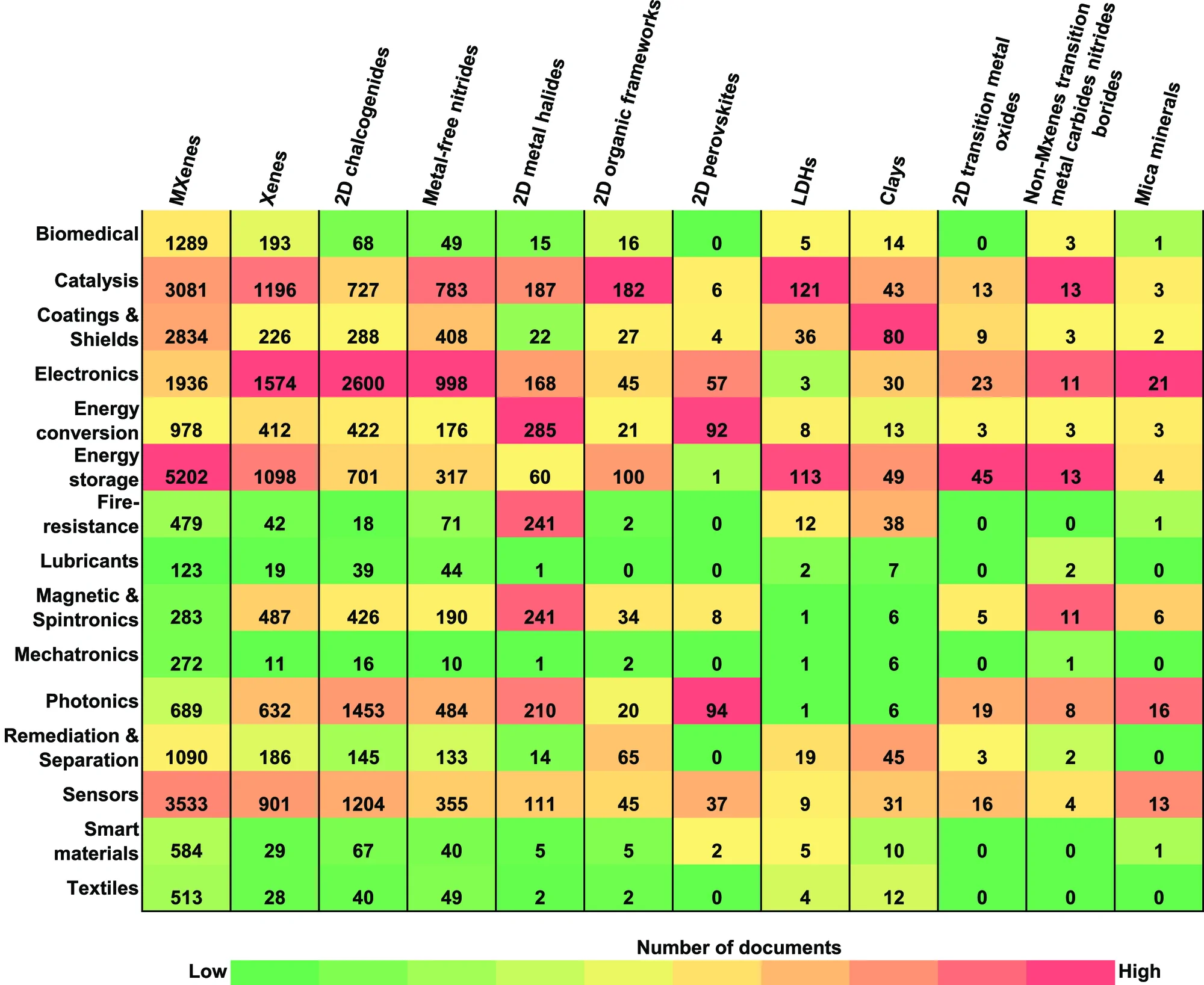
Heatmap presenting the distribution of each 2D material class in various applications. The cells in each column are color coded independent of other columns according to the number of publications in that column and the values in each cell correspond to the number of publications in which the material class and the application co-occur. Data extracted from the CAS Content Collection and includes journal articles and patent publications between 2016 to March 2026.

MXenes emerge as a dominant multifunctional platform, leading prominently in energy storage, sensing, catalysis, and coatings/shielding applications. 2D metal chalcogenides exhibit pronounced specialization in electronics, photonics, and sensing. Xenes demonstrate notable versatility, with substantial contributions to electronics, catalysis, and energy storage. Metal-free nitrides (h-BN and g-C_3_N_4_) show more focused activity in electronics, catalysis, and photonics. Similarly, 2D perovskites display a high degree of specialization in photonics and energy conversion. Several emerging trends are also evident: (1) 2D metal halides demonstrate marked prominence in magnetics/spintronics and fire-resistance applications; (2) 2D organic frameworks (MOFs and COFs) are gaining increasing attention in catalysis, energy storage, and remediation/separation; (3) Layered double hydroxides (LDHs) exhibit focused activity in catalysis and energy storage; (4) Clay-based 2D materials maintain niche yet important roles in coatings and remediation/separation.

Overall, this application–material landscape provides a strategic framework for rational material selection while simultaneously highlighting underexplored opportunities. Mica minerals, transition metal carbides/nitrides/borides and certain metal halides remain comparatively underdeveloped across multiple technological domains, representing promising directions for future research and innovation. In subsequent sections, we provide detailed discussions of individual material families and their application-specific correlations in the context of their physicochemical properties.

## 3 Analysis of Individual Classes of 2D Materials

### 3.1 MXenes

MXenes, a family of two-dimensional transition metal carbides, nitrides, and carbonitrides, have emerged as transformative materials since their discovery in 2011. Characterized by the general formula M_*n*+1_X*_n_* T_*x*_ (where M represents transition metals, X denotes carbon or nitrogen, n is an integer ranging from one to four, and T_*x*_ indicates surface terminations such as −O, −OH, −F, or −Cl), MXenes possess a rare combination of properties that distinguish them from other 2D materials: metallic conductivity paired with hydrophilicity, solution processability at room temperature, and tunable surface chemistry. Unlike graphene’s challenging functionalization, MXenes can be dispersed in water, modified with diverse surface groups, and integrated into composites through simple mixing, making them uniquely accessible for multifunctional applications spanning energy storage, catalysis, electronics, and environmental remediation.

MXenes demonstrate versatility across multiple application domains, with energy storage, sensors, catalysis, and coatings and shields representing the primary research focus. Substantial publication activity also exists in electronics, biomedical, energy conversion, and remediation and separation applications. To systematically investigate structure–property-application relationships, we categorized MXene materials based on four fundamental structural criteria: stoichiometry (M_2_X, M_3_X_2_, M_4_X_3_), transition metal composition, anion type (carbide, nitride, carbonitride), and surface termination chemistry. Analysis of application distributions across these classifications reveals how intrinsic material properties, including electronic structure, interlayer spacing, catalytic activity, and surface functionality, govern application suitability and research emphasis within each MXene subfamily (Figure 5).

#### 3.1.1 MXenes Grouped by Stoichiometry

The distribution of MXene research across different stoichiometries reveals M_3_X_2_ commanding 74% of publications, followed by M_2_X and M_4_X_3_. (Figure 2) Both M_3_X_2_ and M_2_X showed considerable growth in the last three years (Figure 4A).

**Figure 4:**
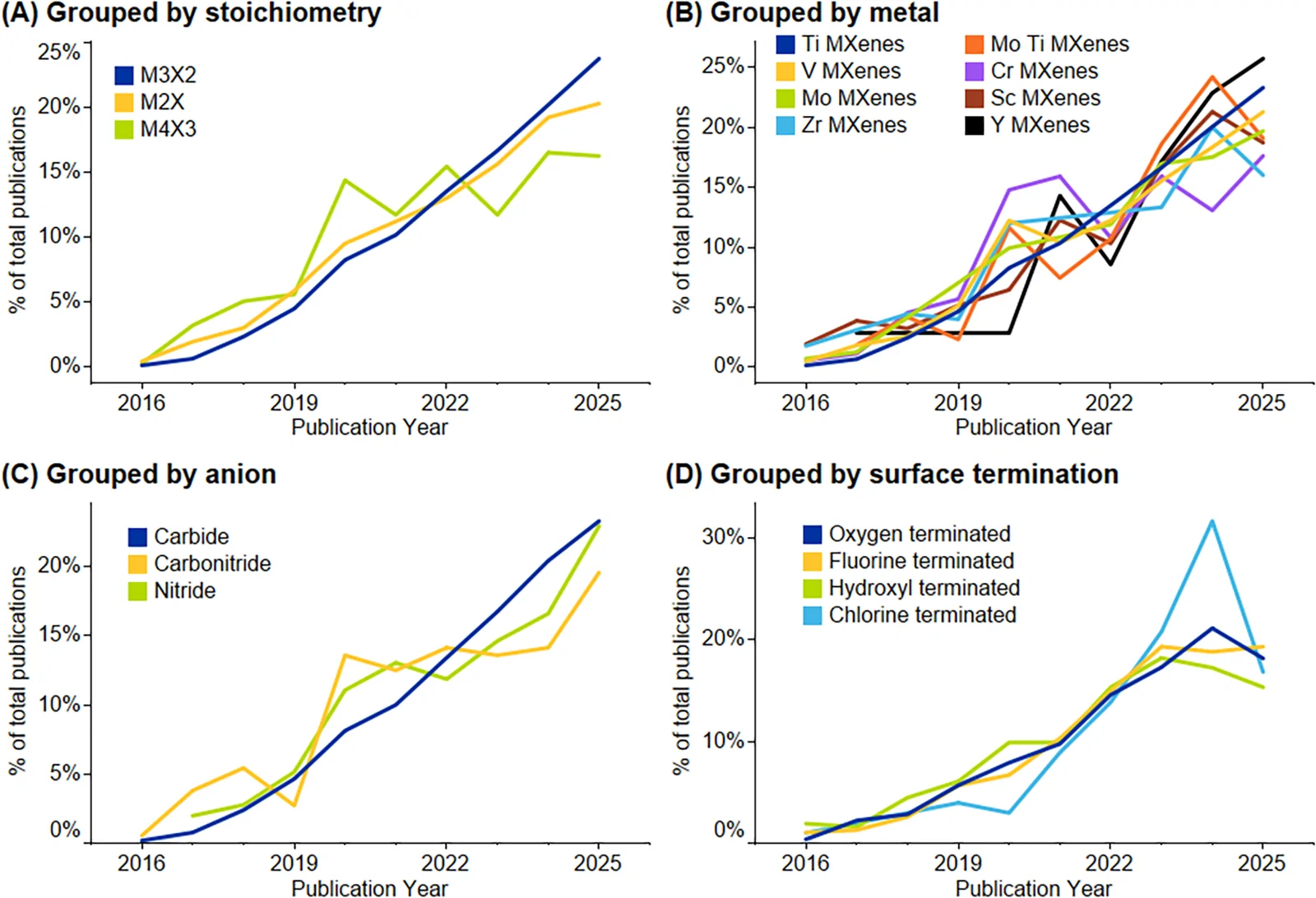
Yearly publication trends of MXene groups with high growth in recent years: (A) grouped by stoichiometry; (B) grouped by metal; (C) grouped by anion; (D) grouped by surface termination. Data is extracted from the CAS Content Collection and includes journal articles and patent publications between 2016 to March 2026.

M_3_X_2_(*n* = 2) dominates the literature, reflecting its superior stability and ease of synthesis compared to M_2_X­(*n* = 1) and M_4_X_3_(*n* = 3). MXene stoichiometry significantly influences application focus, as evidenced by publication distribution. Figure 5A shows the distribution of these MXenes groups in various applications. Energy storage leads across all stoichiometries but shows varying preference with layer thickness of individual MXene layers.^13^ Thicker MXenes (M_4_X_3_) provide more interlayer galleries for ion intercalation, enhancing battery capacity.^14^ Energy storage and catalysis jointly comprise nearly two-thirds of M_2_X publications, the greatest combined proportion across the stoichiometries. This trend is consistent with the shorter ion-transport distances and increased surface accessibility of the thinnest MXene layers.

**Figure 5:**
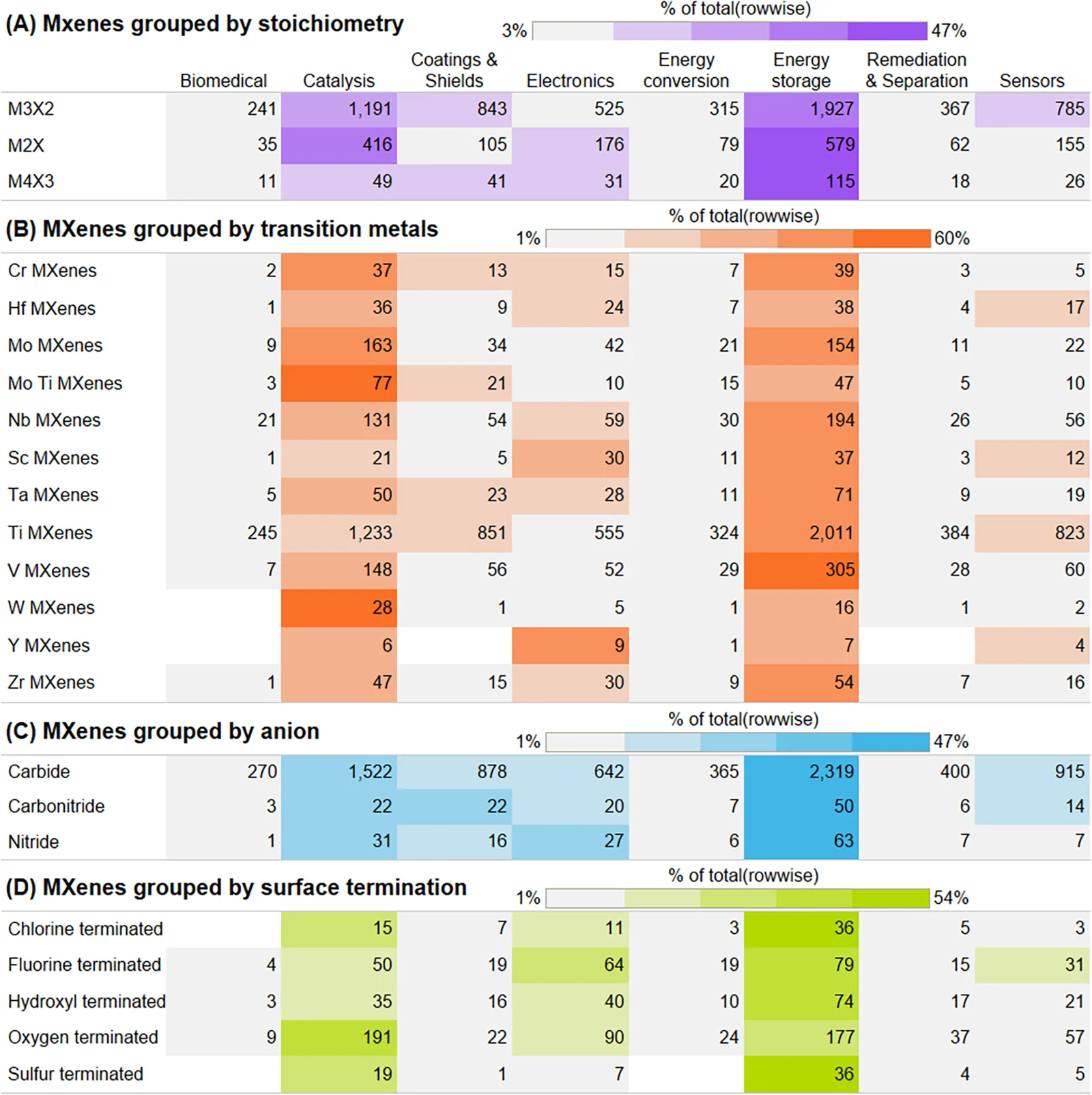
MXenes grouped using various parameters and their distribution in applications. The cells in each row are color coded independently according to the percentage of total publications in each row: (A) MXenes grouped by stoichiometry; (B) MXenes grouped by transition metals; (C) MXenes grouped by anion; (D) MXenes grouped by surface termination. Data is extracted from the CAS Content Collection and includes journal articles and patent publications between 2016 to March 2026.

Catalysis favors M_2_X over M_3_X_2_ and M_4_X_3_, as thinner structures offer higher surface-area-to-volume ratios and more exposed active edge sites for catalytic reactions. The larger surface area of M_2_X facilitates electron transfer and reactant accessibility.^15^ The observed association of M_4_X_3_ MXenes with coatings may reflect their thicker layer architecture and mechanical robustness.^16^ The rarity of M_4_X_3_ publications reflects challenging synthesis, requiring precise MAX phase etching control.^17^

#### 3.1.2 MXenes Grouped by Metals

The distribution of MXene research across transition metal compositions reveals profound insights into how electronic structure dictates application suitability. Ti-based MXenes dominate with 76% of total publications, while other transition metals show specialized application niches correlating with their intrinsic electronic and catalytic properties (Figure 2).

Ti-based MXenes dominate the field, primarily due to Ti_3_C_2_T*_x_*’s accessible synthesis from commercially available Ti_3_AlC_2_ MAX phases, excellent stability, and well-characterized properties. Ti, Y, V, and Mo MXenes showed consistent growth in recent years (Figure 4B) Ti MXenes show balanced application distribution across all fields (Figure 5B). Catalytic activity correlates strongly with metal identity.^18^ Mo and W MXenes exhibit exceptional catalytic focus, attributed to their optimal hydrogen binding energies and multiple oxidation states favoring hydrogen evolution reactions (HER).^15^ Mo–Ti double-metal MXenes achieve the highest catalytic representation, demonstrating synergistic effects from combining metals with different electronic structures.^19^

Energy storage preferences vary systematically: V and Sc MXenes show highest focus, reflecting their multiple accessible oxidation states enabling pseudocapacitive charge storage.^20^ Group V metals (V, Nb, Ta) consistently show 49–60% energy storage focus due to favorable redox activity and ionic intercalation properties. Early transition metals (Sc, Y, Zr, Hf) receive less attention due to challenging MAX phase synthesis and higher reactivity. However, Hf and Zr MXenes show strong catalytic applications, benefiting from high work functions and unique electronic structures.^21^ The scarcity of W and Y publications reflects difficult synthesis and limited MAX phase precursor availability, despite their promising catalytic performance.

#### 3.1.3 MXenes Grouped by Anions

While carbides dominate research volume by 97%, the application distributions expose subtle yet scientifically significant compositional effects (Figure 2). This disparity reflects the synthetic accessibility of carbide MXenes, particularly Ti_3_C_2_T*_x_*, which forms readily through selective etching of MAX phases. The number of publications reporting carbide MXenes has been growing consistently over the years whereas the carbonitride and nitride MXenes grew more recently (Figure 4C).

Application patterns reveal distinct anion-dependent preferences (Figure 5C). Nitride MXenes are often discussed as promising for energy storage owing to favorable electronic structures and ion-transport characteristics, although these properties depend strongly on composition and surface terminations. Nitride MXenes also demonstrate exceptional catalytic activity, attributed to their optimal d-band center positioning and nitrogen-induced electronic modifications that enhance surface reactivity.^22^ Carbonitrides occupy a unique middle ground, showing balanced performance across energy storage and electromagnetic shielding.^23^ Carbide MXenes exhibit the broadest application diversity, including significant representation in sensors, electromagnetic shielding, and biomedical applications, reflecting their versatile surface chemistry, hydrophilicity, and established functionalization protocols. The limited nitride research suggests substantial untapped potential in emerging applications.

#### 3.1.4 MXenes Grouped by Surface Terminations

Surface termination chemistry represents a critical design parameter in MXene applications, and different terminations show dramatically different application preferences (Figure 5D). These distributions reveal how terminal group electronegativity, size, and chemical reactivity fundamentally govern material performance across diverse applications. Surface terminations in MXenes are functional groups that bind to the transition metal surface during the etching of MAX phases. Surface terminations critically influence MXene properties and application suitability, as evidenced by distinct research patterns. Oxygen-terminated MXenes dominate the literature, demonstrating exceptional catalytic activity, the highest among all terminations (Figure 2). In practice, surface terminations are mixed and are influenced by the etchant, with fluoride-based routes preserving appreciable −F.^24^ Nevertheless, −O is prevalent in reported and computationally modeled compositions because it is considered the most thermodynamically stable termination and is the configuration most frequently adopted in computational studies, which constitute a substantial portion of the catalysis literature.^25^ Intentional annealing, alkaline processing, and oxidation under ambient conditions can further elevate the oxygen content.^22^ This reflects oxygen’s ability to create active redox sites and optimal binding energies for reaction intermediates, which is beneficial for electrocatalysis and photocatalysis.^26^ Fluorine terminations contribute to tunable electronic properties and can enhance interfacial stability in specific environments.^27^ Hydroxyl groups confer superior hydrophilicity and solution processability, enabling diverse applications across sensors and remediation.^28^ Their hydrogen-bonding capability facilitates interlayer spacing control and functional molecule anchoring. Notably, chlorine and sulfur terminations exhibit high energy storage focus, attributed to their weaker electronegativity compared to oxygen/fluorine, which enhances ion intercalation kinetics and pseudocapacitive behavior.^29^ However, their synthetic challenges and lower stability limit broader exploration, representing significant opportunities for emerging research in high-performance energy storage systems.

### 3.2 Xenes

The emerging field of 2D Xenes—single-element layered materials beyond graphene—demonstrates remarkable diversity in fundamental properties and technological applications. Analysis of indexed publication data reveals systematic correlations between intrinsic material characteristics and research focus, with distinct trajectories for different elemental families. Understanding these property-application relationships illuminates why certain Xenes dominate specific technological domains while remaining underexplored in others. Phosphorene leads the field with 3,023 publications, followed by silicene and graphdiyne, while heavier elements like gallenene and plumbene remain nascent (Figure 2). This distribution reflects synthetic accessibility and stability considerations, but more fundamentally indicates properties enabling application-focused research. Among the Xenes, graphdiyne, borophene, graphyne, tellurene and, plumbene showed high growth in recent years (Figure 6).

**Figure 6:**
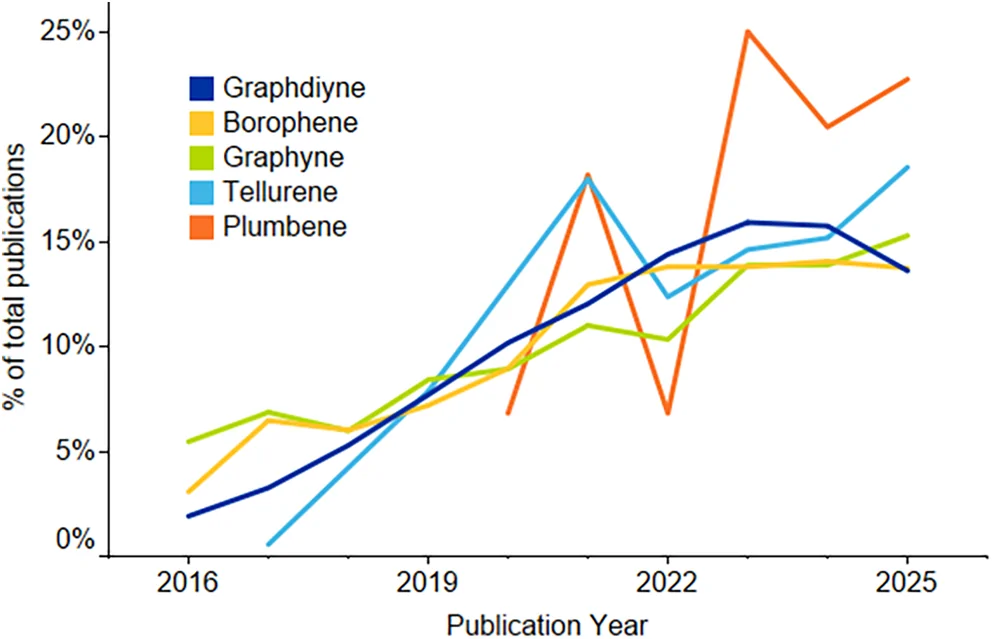
Yearly publication trends of Xenes with high growth in recent years. Data is extracted from the CAS Content Collection and includes journal articles and patent publications between 2016 to March 2026.

#### 3.2.1 Applications and Properties

Electronic property investigations dominate research in pnictogen and carbon group Xenes (Figure 7A). Band structure and band gap studies are present in 50–75% of publications involving arsenene, antimonene, germanene, tinene, silicene, and tellurene. This intensive electronic characterization is consistent with electronics applications where these Xenes are dominant (Figure 7B). These materials exhibit layer-dependent bandgaps transitioning from indirect (bulk) to direct (monolayer configurations), with values ranging from 0.3 to 2.5 eV, which is ideal for field-effect transistors^30^ and logic circuits.^31^ In electronics applications, these Xenes function as channel materials in transistors,^32^ active layers in photodetectors,^33^ and switching elements in neuromorphic computing.^34^

**Figure 7:**
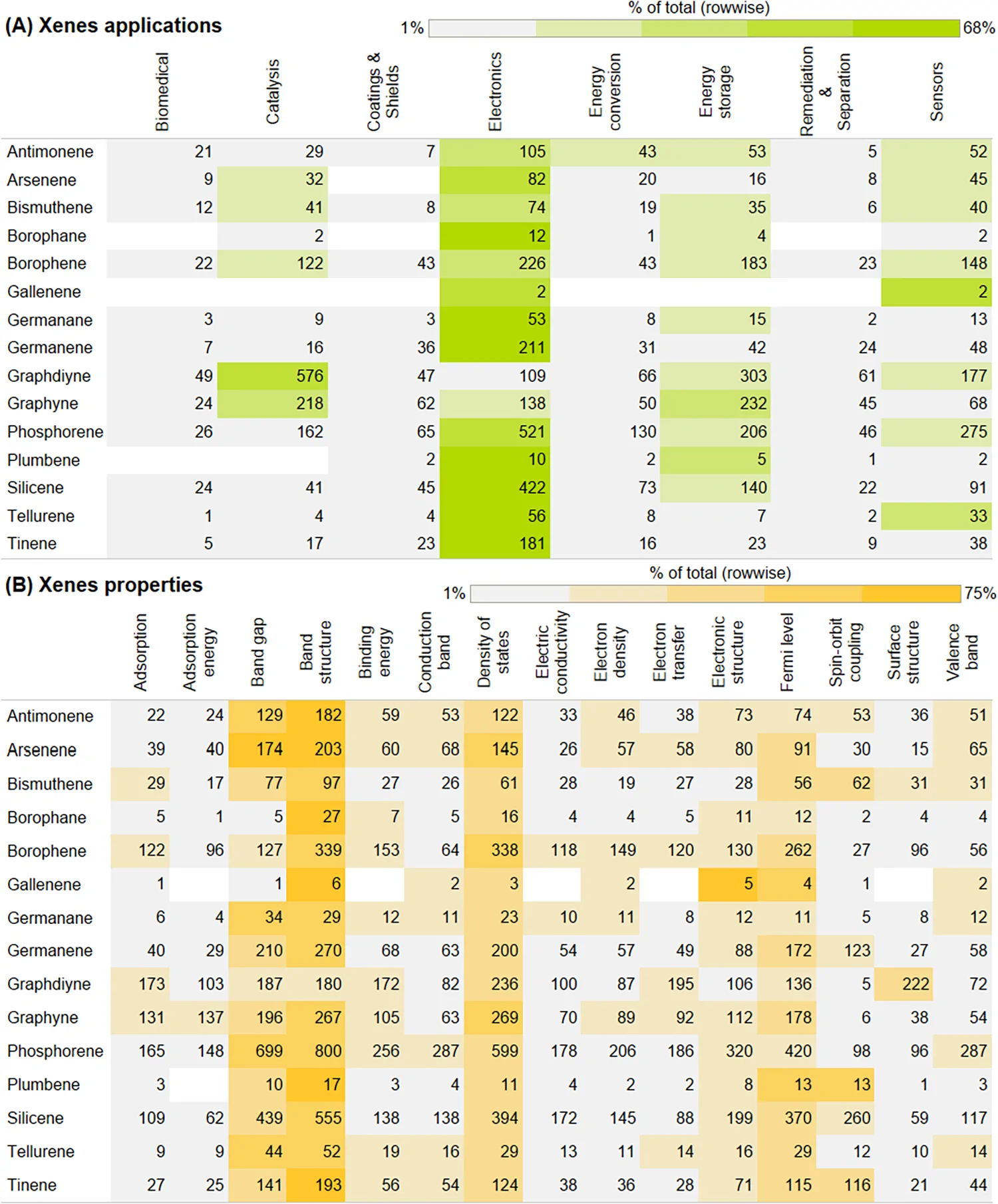
Distribution of Xenes in (A) applications; (B) properties. The cells in each row are color coded independently according to the number of publications. Data is extracted from the CAS Content Collection and includes journal articles and patent publications between 2016 to March 2026.

Borophane offers a fascinating case: electronics drive 60% of its applications, compared to just 33% for the naturally metallic borophene. Hydrogenation fundamentally alters electronic structure, opening a bandgap in otherwise metallic borophene.^35,36^ This chemical modification demonstrates how surface functionalization can redirect application potential. Borophene’s significant catalysis interest is due to its metallic nature and corrugated structure providing diverse adsorption sites with variable binding strengths, mimicking precious metal catalysts for various reactions.^37^ Borophene’s polymorphic, anisotropic metallic lattices and high theoretical ion-storage capacity promote energy-storage applications, although oxidation under ambient conditions continues to impede stable device integration.

Carbon-based Xenes prioritize surface interactions over electronic properties. Graphdiyne exhibits the field’s most extreme application specialization with 49% catalysis focus. This dominance correlates with property research emphasizing adsorption, surface structure, and electron transfer. Graphdiyne’s sp-sp^2^ hybridized framework creates uniformly distributed triangular nanopores (∼5 Å diameter) with surface areas exceeding 1000 m^2^/g.^36,38^ These structural features provide abundant catalytic active sites, molecular accessibility, and excellent electrical conductivity for electron transfer during electrochemical reactions. Graphdiyne functions as an electrocatalyst for oxygen reduction reactions (ORR) in fuel cells, hydrogen evolution for water splitting, and CO_2_ reduction to value-added chemicals.^39^ Its acetylenic linkages provide π-conjugated pathways facilitating electron transfer between electrodes and adsorbed reactants, while uniform pores enable size-selective catalysis. Graphyne with 31% of applications focused on catalysis offers similar advantages with different pore geometries, enabling complementary selectivity in molecular transformations. Energy storage applications show balanced distribution: graphyne, borophene, and graphdiyne lead through structural advantages. Intrinsic porosity and high surface area accommodate lithium/sodium intercalation sites, while mechanical flexibility prevents structural degradation during charge–discharge cycling.^40^

Pnictogen Xenes such as arsenene and phosphorene are mostly applied in sensing through combined electronic and surface properties. Arsenene, phosphorene, tellurene, and borophene demonstrate substantial sensor emphasis. Phosphorene’s puckered lattice imparts pronounced in-plane anisotropy, layer-tunable direct bandgaps, and high hole mobility, making it attractive for transistors and sensors, although degradation under ambient conditions often necessitates encapsulation. Tellurene is applied widely in sensors due to its narrow bandgap enabling high sensitivity to environmental perturbations, strong light absorption for optoelectronic sensing, and surface reactivity for chemical detection. Tellurene’s helical-chain structure further imparts environmental stability and pronounced spin–orbit coupling, facilitating infrared photodetection and differentiating it from the air-sensitive pnictogen Xenes. Antimonene has attracted biomedical interest because selected studies report photothermal activity and acceptable biocompatibility under specific experimental conditions.^41^

### 3.3 2D Metal Chalcogenides

2D metal chalcogenides represent a broad class of layered materials composed of chalcogens, sulfur (S), selenide (Se) or telluride (Te), combined with at least one electropositive metal element such as molybdenum (Mo), titanium (Ti), or tungsten (W). Owing to their compositional diversity and tunable physicochemical properties, these materials occupy a prominent position within the broader 2D materials landscape, as illustrated in the material map (Figure 2).

To gain further insights into the research and innovation landscape, this analysis focuses on key subclasses of 2D metal chalcogenides. These include transition metal dichalcogenides (TMDs), which are among the most studied systems, emerging variants such as Janus TMDs (characterized by asymmetric chalcogen layers) as well as TMD-based alloys, which enable compositional and structural tuning.

Beyond transition metals, this class also encompasses 2D chalcogenides based on p-block metals (e.g., Bi, Ga). These materials introduce additional chemical diversity and potentially novel functionalities, thereby enriching the overall landscape of 2D chalcogenide research.

#### 3.3.1 Transition Metal Dichalcogenides

TMDs represent a prominent class of 2D materials (Figure 2) characterized by a layered van der Waals structure with the general stoichiometric formula MX_2_, where M is a transition metal (e.g., Mo, W, Ti, V) and X is a chalcogen (S, Se, or Te). These materials consist of atomically thin layers with strong in-plane bonding and weak interlayer interactions, enabling easy exfoliation into two-dimensional sheets.^42^

We analyzed TMDs using a hierarchical grouping framework based on structural and compositional features. Materials are first classified into structural categories: conventional-, nonconventional-, Janus-, tunable-, and alloy TMDs reflecting increasing complexity and design flexibility. Within each group, they are further categorized by transition metal identity and anion (chalcogen) type, highlighting how compositional variation drives differences in research activity and functional focus. To connect these material classifications with practical applications, we further analyzed the co-occurrence of TMDs with key application domains (Figure 3 and Figure 8).

**Figure 8:**
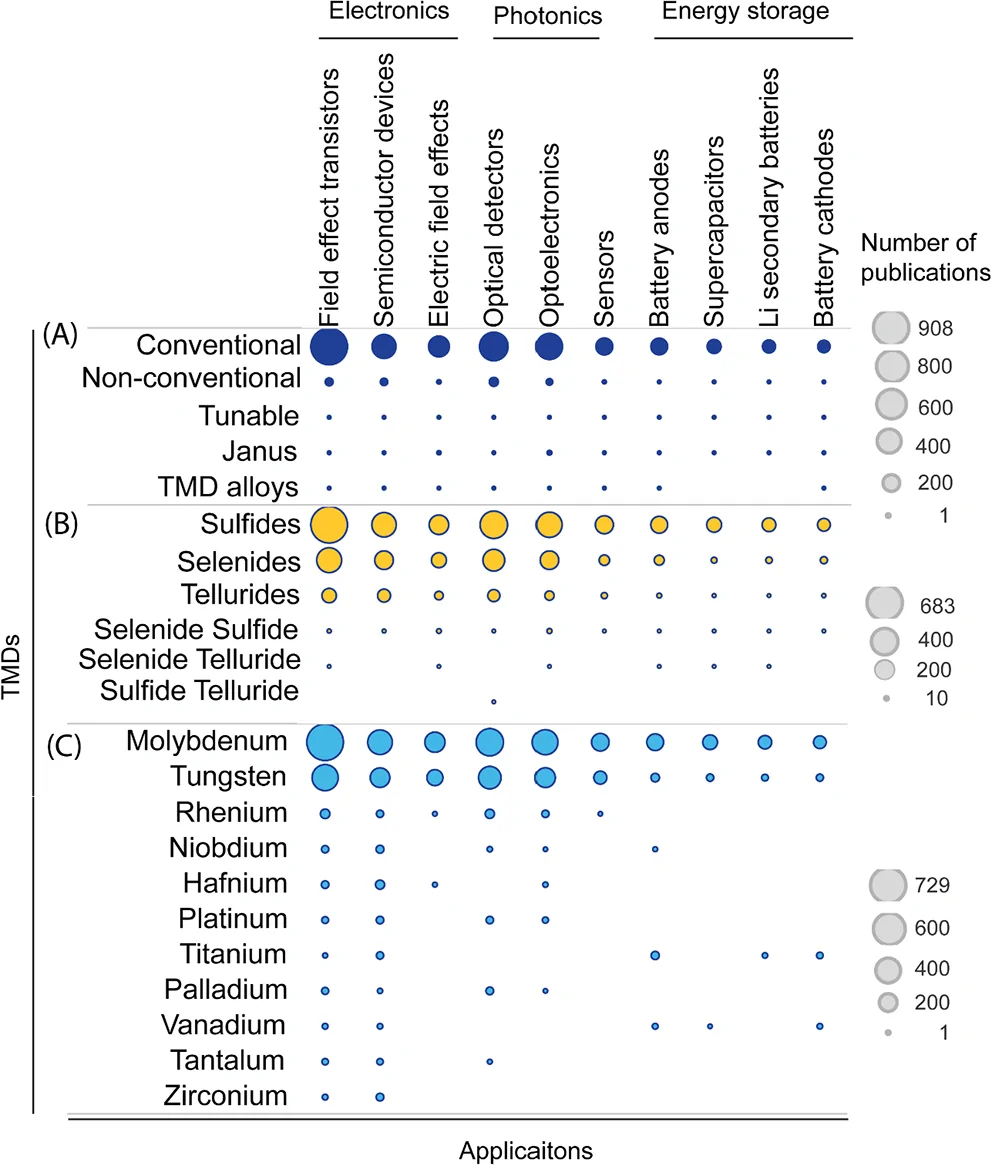
Co-occurrence of application concepts and TMDs: categorized (top to bottom) (A) by material group; (B) anion type; and (C) metal. Data is extracted from the CAS Content Collection and includes journal articles and patent publications between 2016 to March 2026.

In the following sections, these material subclasses and their corresponding applications are discussed together, enabling a direct mapping between structural/compositional features and functional outcomes across the TMD landscape.

##### 3.3.1.1 Structural and Compositional Group

TMD research spans conventional systems that form the foundation of 2D materials to nonconventional, alloyed, and Janus variants that expand the compositional and structural design space, enabling targeted tuning of electronic, catalytic, and functional properties.

Conventional TMDs, such as MoS_2_ and WS_2_, dominate the literature and serve as benchmark semiconducting systems. These are followed by TMDs of other d-block transition metals (TMs), primarily from groups IV–VII, which exhibit more metallic or structurally complex behavior compared to the widely studied group VI semiconducting systems such as MoS_2_ and WS_2_.^43^ These TMDs exhibit a strong coupling between composition and electronic structure, across different application domains (Figure 8).

Nonconventional TMDs typically involve noble or heavy TMs. They are typically highly conductive but also expensive and difficult to scale. These materials represent niche or emerging research spaces, often driven by specific high-performance applications rather than scalability.^44^ They are less prominent in electronics but show stronger relevance in specialized applications such as sensing, catalysis, and energy storage, where high conductivity and surface activity are advantageous.

Janus TMDs are structurally modified systems in which two chalcogen layers differ (MXY instead of MX_2_), for example, MoSSe and WSSe. These exhibit key characteristics including broken out-of-plane symmetry and a built-in electric dipole, which offers enhanced charge separation as well as piezoelectric properties.^45^

TMD alloys are systems in which composition is tuned by mixing elements. These alloys act as a bridge between conventional systems and advanced material design, as they enable continuous tuning of electronic, optical, and chemical properties through compositional variation while preserving the underlying crystal structure.^46^

##### 3.3.1.2 Anion-Based Group

Anion chemistry further influences functional specialization. Sulfide TMDs dominate all applications followed by selenides and tellurides. Sulfide-based TMDs (e.g., MoS_2_, WS_2_) are structurally stable, semiconducting 2D materials with strong excitonic behavior, making them foundational systems for electronics, photonics and energy storage applications.^47^

Selenide-based TMDs with moderate bandgaps and strong light–matter interaction, as well as pronounced spin–orbit and excitonic effects, making them suitable for optoelectronics and photonic applications.^48,49^ In particular, WSe_2_ and MoSe_2_ are widely explored for excitonic and valley polarization phenomena, while heterostructures based on these materials enable tunable band alignment and enhanced photoresponse.^50^

While telluride-based TMDs (e.g., MoTe_2_, WTe_2_) exhibit small bandgaps, strong spin–orbit coupling, and rich phase polymorphism. This enables tunable electronic properties and makes them promising for phase-change electronics, infrared optoelectronics, and topological quantum applications.^51^

Mixed chalcogenide TMDs (e.g., MoSSe, MoSeTe) exhibit broken out-of-plane symmetry and composition-dependent electronic structure, leading to intrinsic electric fields, tunable bandgaps, and improved carrier separation, which are advantageous for photocatalysis, photovoltaic, and next-generation electronic applications. These TMDs introduce compositional and structural asymmetry that enables bandgap engineering, intrinsic dipole formation, and efficient charge separation;^52,53^ however, their practical deployment is hindered by challenges in multicomponent synthesis, phase stability, and compositional uniformity.^54^ Also, thermodynamic limitations often lead to phase segregation and disorder,^55^ while incomplete understanding of alloy phase behavior and interface effects limits scalable device integration compared to conventional Mo- and W-based TMDs.

##### 3.3.1.3 Metal-Based Group

This perspective highlights that electronic behavior across the TMD family is largely governed by the choice of transition metal. Although Mo- and W-based TMDs are often highlighted due to their technological relevance, the broader TMD family is largely composed by metallic and semimetallic systems,^56^ particularly those based on Ti, Nb, Ta, and Pt, where partially filled d-orbitals give rise to high conductivity and correlated electronic phases such as charge density waves and superconductivity.^57^ While these materials are fundamentally important for studying quantum and transport phenomena, their lack of intrinsic bandgaps limit their applicability in conventional electronic and optoelectronic devices (Figure 8).^58^

In this predominantly metallic landscape, Mo- and W-based TMDs therefore represent a distinct and technologically critical subset, with semiconducting 2H phases and suitable bandgaps (∼1.2 eV). These properties underpin their widespread use in field-effect transistors, optoelectronic devices, and heterostructures, where efficient charge transport, gate modulation, and light–matter interactions are essential. Their sulfides and selenides dominate applications in electronics and photonics, supported by strong excitonic effects, high photoluminescence, and tunable band alignment.

In contrast, TMDs based on Ti, Nb, Ta, and related metals, which exhibit metallic or semimetallic character, are preferentially associated with sensing and energy storage applications in addition to electronic and photonics. Their high electrical conductivity, surface reactivity, and ability to facilitate charge transfer and ion intercalation make them well suited for sensors, supercapacitors, and battery electrodes. Thus, semiconducting group VI TMDs primarily enable electronic and optoelectronic functionalities, whereas metallic and defect-engineered systems drive electrochemical and surface-driven applications through enhanced conductivity and interfacial activity.^59,60^ Meanwhile, sensing and energy storage applications, including sensors, battery electrodes, and supercapacitors, are driven by high surface reactivity, electrochemical activity, and the ability of TMDs to facilitate ion intercalation and redox processes.^59^

This property–application relationship highlights that the observed application clusters are fundamentally dictated by the electronic structure, optical response, and surface chemistry of TMD materials. These applications are dominated by MoS_2_, VS_2_, TiS_2_, and NbSe_2_, where metallic or semimetallic character and high surface area enhance charge transfer and catalytic activity.^60^

##### 3.3.1.4 Emerging Material Groups

TMD research trend from 2016 to 2025 shows a clear shift toward novel structures and metal compositions. (Figure 9) Tunable TMDs exhibit the strongest growth, rising from ∼3% before 2021 to ∼26% by 2025, while Janus and mixed chalcogenide systems similarly expanded to ∼25%. In contrast, traditional sulfide-based TMDs have plateaued, indicating a transition away from conventional architectures.

**Figure 9:**
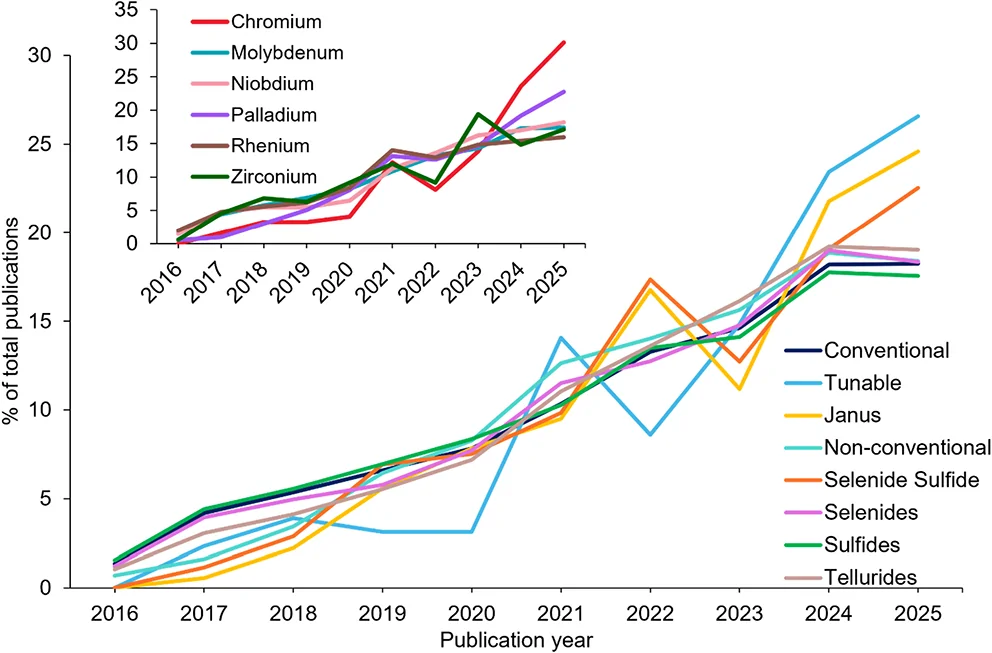
Relative growth rate of material groups in TMDs. Data is extracted from the CAS Content Collection and includes journal articles and patent publications between 2016 to March 2026.

On the metal side, chromium-based TMDs show the most rapid growth, increasing from ∼5% in 2022 to ∼30% in 2025, followed by a late surge in molybdenum-based systems. Meanwhile, earlier leaders such as zirconium, palladium, niobium, and rhenium have stabilized, reflecting maturity. Overall, these trends highlight a shift from conventional Mo/W-based sulfides toward structurally and compositionally diverse next-generation TMD systems.

#### 3.3.2 Other 2D Chalcogenides

In addition to TMDs, a broader class of 2D chalcogenides based on post-transition metals and metalloids (e.g., In, Bi, Sn, Ga, Ge, and Sb) was analyzed as a distinct subgroup. These materials (in Figure 10A) were separated from TMDs due to fundamental differences in their chemical composition, electronic structure, and crystal architecture. Unlike TMDs, which are characterized by MX_2_ stoichiometry and d-orbital-dominated electronic behavior, these systems exhibit diverse stoichiometries (e.g., MX, M_2_X_3_) and are governed by p-orbital interactions.^57,61^ This results in different physical properties, including topological insulation and anisotropic transport (Figure 10B). Furthermore, their crystal structures often deviate from the typical layered polymorphs observed in TMDs, encompassing puckered or quintuple-layered frameworks. As a result, this class of chalcogenides occupies a distinct materials space with unique property–application relationships.

**Figure 10:**
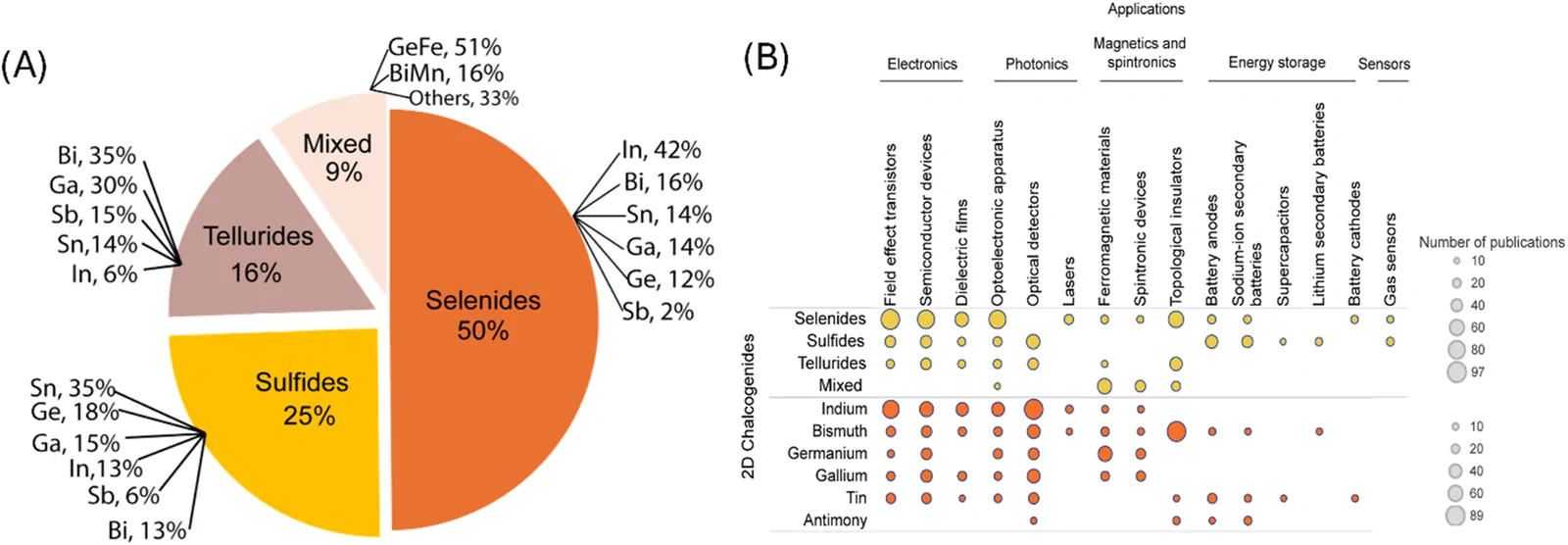
(A) Distribution of other 2D chalcogenides by anion and metal type; (B) Co-occurrence of application and material groups. Data is extracted from the CAS Content Collection and includes journal articles and patent publications between 2016 to March 2026.

##### 3.3.2.1 Material-Application Relationships

These chalcogenides are categorized based on their constituent anions and metal elements, as shown in Figure 10A. To further link material composition with application domains, we analyzed their co-occurrence with prominent applications (Figure 10B; see also Figure 3).

One distinct observation is that, unlike TMDs, which are dominated by sulfide compositions owing to their stability within the MX_2_ layered framework, non-TMD p-block chalcogenides show a clear compositional shift toward selenides (Figure 10A). This trend arises from the greater structural flexibility and enhanced orbital compatibility of Se with post-transition metals, enabling stabilization of varied stoichiometries and crystal structures beyond the conventional TMD phases.^62,63^ The high concentration of In-based compounds highlights their central role in 2D semiconductor applications,^64^ while the strong presence of Bi-based systems reflects interest in topological and optoelectronic functionalities (Figure 10B). This indicates that selenides form the core materials platform for p-block 2D chalcogenide research.

Sulfides, while representing a smaller share, exhibit a more compositionally diverse landscape, with tin sulfide (Sn_*x*_S_*x*_) dominating but significant contributions from germanium (Ge), gallium (Ga), bismuth (Bi), and indium systems. This suggests that sulfides act as a broader but less specialized materials space, often serving as alternatives where stability, cost, or synthesis considerations are important. They also exhibit less specialized usage, particularly in energy storage, where Sn-based systems bridge sulfides and selenides, highlighting their versatility across conventional and emerging applications.^65^

Tellurides, though the least represented, show targeted concentration around specific high-value systems, particularly Bi- and Ga-based compounds. Their presence points to niche but high-impact applications, likely associated with advanced functionalities such as topological behavior. Bismuth spans all three categories but is prominent in tellurides, indicating its role in specialized quantum and thermoelectric systems.^66^

Finally, the emergence of mixed-anion systems signals a transition in the field from material selection to material design, where hybrid compositions are increasingly explored to achieve multifunctional and tunable properties beyond those available in pure compounds. Across application domains, the emergence of Ge-containing mixed systems further highlights growing interest in hybrid compositions for tunable properties through alloying and bandgap engineering.^67^ Overall, these trends underscore a transition from material discovery toward application-specific design, where elemental roles and chalcogen chemistry are strategically aligned with device requirements.

### 3.4 Metal-Free Nitrides

Metal-free nitrides, particularly boron nitride (BN) and carbon nitride, have emerged as versatile materials with distinct property profiles that direct their application domains. Analysis of publication data reveals a clear divergence in how these materials are used, driven by their fundamental structural and electronic characteristics. The number of publications reporting both nitrides showed significant growth in recent years (Figure 11A). Figure 11B shows the distribution of these two materials in various applications.

**Figure 11:**
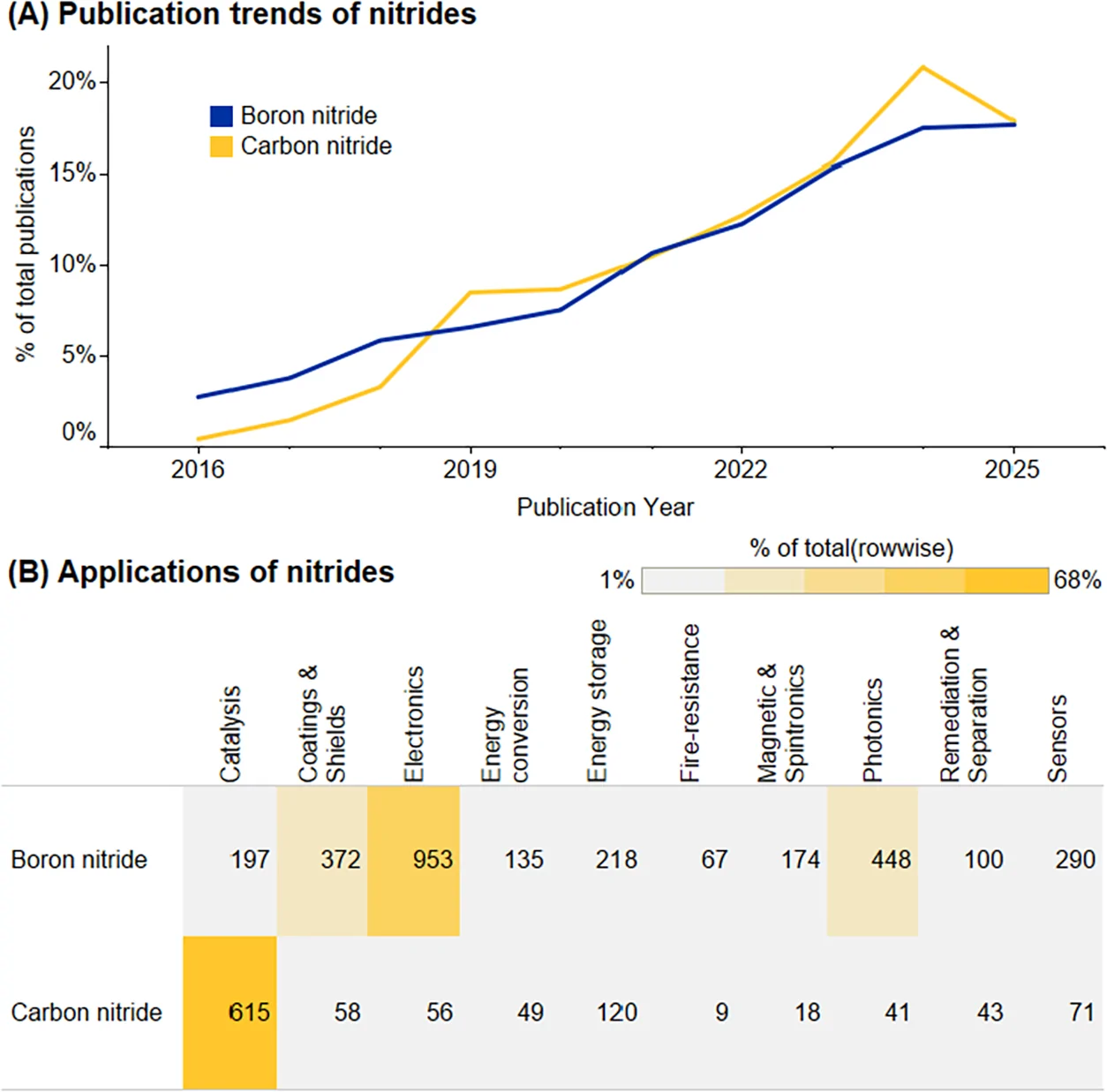
(A) Publication trends of metal-free nitrides during the period 2016–2025; (B) Heatmap showing the distribution of metal-free nitrides in various applications. The cells in each row are color-coded independently according to the number of publications. Data is extracted from the CAS Content Collection and includes journal articles and patent publications between 2016 to March 2026.

#### 3.4.1 Boron Nitride: Electronics and Photonics Focus

Boron nitride is prevalent in electronics applications, accounting for 44% of its total applications due to its exceptional dielectric properties and thermal stability. Its wide bandgap (approximately 5–6 eV), chemical inertness, and excellent electrical insulation make it indispensable for next-generation electronic devices such as gate dielectric in field effect transistors. Hexagonal BN (h-BN) has revolutionized electronics by serving as an ideal substrate and encapsulation layer for two-dimensional materials like graphene. Its structural similarity to graphene, combined with insulating properties, allows for enhanced carrier mobility in channel material of transistors while preventing electrical leakage.^68^ Photonics represents another major application area, driven by boron nitride’s unique optical properties. Recent discoveries of single-photon emitters in h-BN have catalyzed research in quantum photonics, reflected in publications on optoelectronics, electroluminescent devices, and lasers. The substantial presence in coatings and shields stems from boron nitride’s exceptional thermal conductivity combined with electrical insulation, which is a rare combination. With in-plane thermal conductivity rivaling that of metals while remaining electrically insulating, h-BN excels as a thermal conductor in applications requiring heat dissipation without electrical interference.^69,70^ Its chemical stability and high-temperature resistance (stable beyond 1000 °C in inert atmospheres) further support its use in electromagnetic shields^71^ and protective coatings.^72^ When mixed with conductive materials, h-BN provides the insulating matrix that scatters radiation waves and prevents short circuits. Simultaneously, its high thermal conductivity safely dissipates the trapped electromagnetic heat.

#### 3.4.2 Carbon Nitride: Applications in Catalysis

In stark contrast to boron nitride, carbon nitride shows overwhelming dominance in catalysis applications (68%), establishing it as the premier nonmetal photocatalyst. Graphitic carbon nitride (g-C_3_N_4_) possesses a moderate bandgap (approximately 2.7 eV) that enables visible light absorption—a critical advantage over traditional UV-active photocatalysts like titanium dioxide. This property correlates with publications on photocatalysts. The material’s nitrogen-rich framework provides built-in active sites for catalytic reactions, while its electronic structure facilitates charge separation necessary for photocatalytic processes. The moderate presence in energy storage and sensors demonstrates carbon nitride’s versatility beyond catalysis. Its high nitrogen content and layered structure enable lithium-ion intercalation in batteries, while the nitrogen sites provide chemical sensitivity for sensing applications.

### 3.5 Two-Dimensional Metal Halides

The landscape in Figure 2 identifies metal halides as a significant contributing class. 2D metal halides are an emerging class of layered materials composed of metal cations (e.g., Pb, Sn, Bi) coordinated with halide anions (Cl^–^, Br^–^, I^–^), forming extended inorganic sheets or networks. Unlike their 3D counterparts, 2D metal halides and oxyhalides often exhibit strong in-plane bonding with weak interlayer interactions, leading to anisotropic charge transport, tunable band structures, and enhanced surface reactivity.^73^

The diversity in 2D metal halides largely arises from how metal–halide units are arranged into layers, which can be grouped into MX_2_ (e.g., CdI_2_-type), MX_3_ (e.g., BiI_3_, CrI_3_-type), and oxyhalide (MOX, e.g., BiOCl). These structural types govern exfoliation behavior, electronic properties, and applications. In their two-dimensional form, these materials typically consist of metal–halide coordination layers separated by weak van der Waals gaps, enabling exfoliation into ultrathin nanosheets.^74^ Notably, they also serve as the structural and chemical foundation of metal halide perovskites, one of the most dominant and rapidly growing material families.

The material landscape in Figure 12 reflects a combination of material performance, ease of synthesis, application relevance, and historical momentum in 2D materials research. The dominance of certain compounds reveals how the field prioritizes materials that offer exceptional properties for high-impact applications.

**Figure 12:**
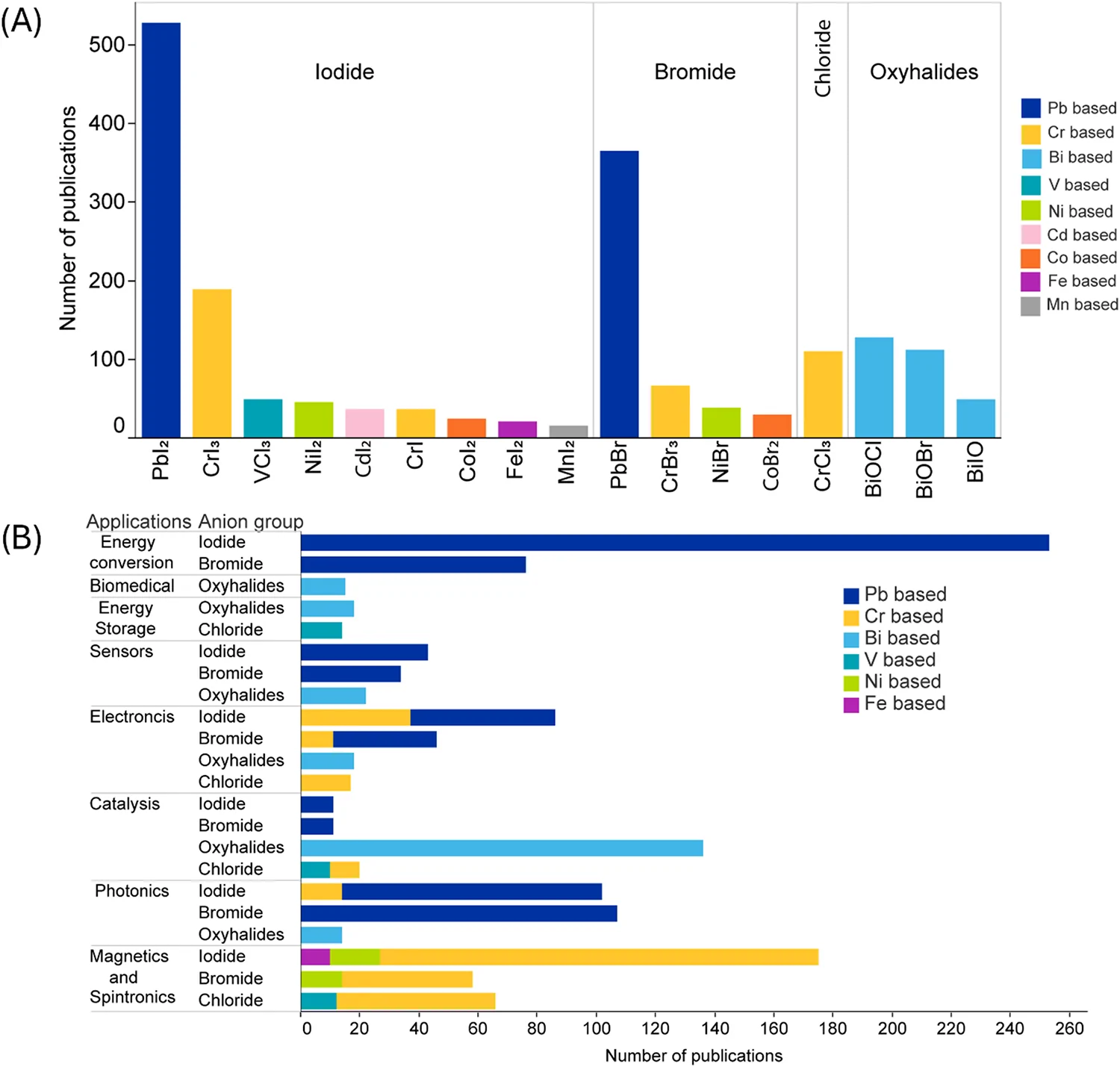
Publication distribution of 2D metal halides: (A) by halide- and metal-type; (B) co-occurrence of application and halide groups color coded by metal type (minimum limit is 10 documents per co-occurrence). Data is extracted from the CAS Content Collection and includes journal articles and patent publications between 2016 to March 2026.

#### 3.5.1 Material and Application Landscape

The publication volume across 2D metal halides varies across by structural class (Figure 12A): MX_2_ systems dominate due to their ideal van der Waals layered structure and optoelectronic functionality, MX_3_ systems gain attention for emergent magnetic phenomena, while oxyhalides represent a growing class leveraging layered heteroanionic structures for stability and catalytic activity.

Iodide-based halides dominate the field due to their larger atomic radius and lower electronegativity, which results in bonds with greater polarizability. This results in narrower bandgaps, better charge carrier mobility, and stronger light absorption suitable for various energy conversion and photonic applications.^75,76^

Whereas metal bromides possess intermediate properties between iodides and chlorides, bromides (bandgaps wider than iodides) are sometimes used to tune bandgaps of iodide-based halides systems as it offers better stability.^77^ While metal chloride exhibits the widest bandgaps among halide systems, their smaller ionic radii and higher electronegativity reduce orbital overlap between metal and halide ions, leading to more localized electronic states and limited optical absorption.^78^ This trend (I → Br → Cl) results in a systematic increase in bandgap and reduced light-harvesting efficiency.^79^ Additionally, the stronger ionic bonding and lower polarizability of chlorides contribute to higher chemical and thermal stability, which, while advantageous for durability, often limits their applicability in optoelectronic and energy-conversion applications.^80^

The role of metal-based groups is more clearly understood when considered alongside their applications as shown in Figure 12B. Pb-based halides (e.g., PbI_2_, PbBr_2_) frequently appear in energy conversion research as they are key precursors and intermediate phases in perovskite solar cells. They react with organic/inorganic cations to form the photoactive lead halide perovskites (e.g., MAPbI_3_, MAPbBr_3_).^81^ In addition, small residual PbI_2_/PbBr_2_ can passivate defects, improving efficiency and stability.^82^ Their layered structures also help control film formation and crystallization, enabling high-quality perovskite layers and better device performance.^83^ Whereas Cr-based halides (CrI, CrI_*x*_, CrBr_3_, CrCl*_x_*) show a distinct application profile due to their intrinsic magnetic properties. For example, CrBr_3_ and CrI_3_, widely studied for 2D spintronic devices and magnetic heterostructures, while CrCl_2_ and CrCl_3_ exhibit antiferromagnetic or tunable magnetic behavior, useful for studying low-dimensional magnetism and phase transitions.^84,85^ Their moderate presence in electronics stems from this magnetic functionality.^86^ In addition, CrCl_3_ also shows emerging potential in photocatalysis, where its 2D structure enables interaction with water, influencing band gaps and band edge positions. These interfacial effects can enhance charge transfer and catalytic activity, making it a candidate for photo­(electro)­catalytic applications.^87^ Overall, this highlights how electronic structure and magnetism dictate application domains: Cr halides for magnetic technologies, versus Pb halides for photovoltaics.

Another prominent halide system in Figure 12B is the Bi-based oxyhalides (BiOBr, BiOCl), especially within catalysis application. The halides Br^–^ and Cl^–^ act as tuning elements, controlling band gap and light absorption (BiOBr is more visible-light active, while BiOCl is more UV-responsive), influence band edge positions and redox ability, and contribute to an internal electric field that promotes efficient electron–hole separation.^88,89^ They also affect surface reactivity and adsorption behavior, further enhancing photocatalytic efficiency.^90^

Bismuth oxyhalides are important for photocatalysis due to their layered 2D structures and suitable band alignments, enabling applications in pollutant degradation, CO_2_ reduction, and chemical production. They are also used in biomedical applications, driven by their ability to generate reactive oxygen species (ROS) under light or ultrasound, enabling effective broad-spectrum antibacterial activity.^91^ Their 2D layered structure, tunable defects, and heterostructures enhance charge separation and therapeutic efficiency, making them suitable for wound healing, implant coatings, and antimicrobial water purification. Combined photothermal and photodynamic effects further strengthen their role as versatile, biocompatible materials for infection control.^92–94^

Vanadium halides (VCl_3_) are primarily studied for correlated magnetism and spintronics, showing antiferromagnetism, Mott insulating behavior, and topological spin textures.^95^ Nickel halides play a dual role, contributing to magnetic materials and acting as dopants to enhance optoelectronic properties (e.g., perovskites).^96^ Iron halides are key 2D ferromagnets with strong potential for high Curie temperature spintronic devices.^97^

Overall, metal halides show a clear functional divergence based on their electronic structure—materials with suitable band structures are used in optoelectronics and energy conversion, while those with strong magnetic interactions are directed toward magnetics and spintronics. Meanwhile, systems with layered structures and tunable defects are increasingly applied in catalysis and biomedical fields, where interfacial reactivity and charge dynamics are critical.

### 3.6 2D Organic Frameworks

2D organic frameworks constitute an important class of materials, as illustrated in Figure 2, including metal–organic frameworks (MOFs) and covalent organic frameworks (COFs). These materials are classified as 2D systems due to their layered structures, ability to form ultrathin nanosheets, high surface area, and tunable chemical architectures, which collectively enable unique physicochemical properties and broad application potential.

#### 3.6.1 2D MOFs

Within organic frameworks, 2D MOFs have emerged as a significant subclass, distinguished by the presence of metal nodes coordinated with organic linkers, which enable precise chemical programmability.^98^ Owing to their accessible coordination environments and modular framework design, 2D MOFs have attracted sustained interest for applications in catalysis, sensing, electronics, and energy storage.^99^ Furthermore, their modular nature allows targeted tuning of structural and functional properties through variation of metal centers and linkers, enabling fine control over framework performance.

To systematically assess how metal centers and donor-atom-defined organic linkers influence the prevalence and functional relevance of 2D-MOFs, we analyzed substance data indexed in CAS databases, focusing on frameworks explicitly described as two-dimensional or layered. This analysis indicates that Cu- and Ni-containing frameworks dominate the reported 2D-MOF landscape, followed by Co, Zn, and Fe to a lesser extent (Figure 2). The prevalence of Cu and Ni in 2D MOFs can be rationalized by their d^9^ and d^8^ electronic configurations, which often favor square-planar or near-planar coordination environments that are conducive to stabilizing layered framework architectures.^100^ Beyond geometric considerations, both metals form relatively strong coordination bonds with heteroatom-containing organic linkers, which can contribute to improved thermal stability and, in some cases, enhanced resistance to hydrolysis, depending on the framework structure and ligand environment.^101,102^ From a practical standpoint, Cu and Ni precursors are inexpensive, earth-abundant, and readily crystallize under mild synthetic conditions using well-established coordination chemistry principles.^103^

Among the organic linkers, N-donor and O-donor linkers exhibit comparable prevalence, while S-donor linkers are represented to a much lesser extent (Figure 2). The near parity between N-donor and O-donor linkers marks a fundamental departure from the broader MOF literature, where carboxylate-based O-donors dominate. This shift reflects the functional demands of 2D-MOFs, particularly electrical conductivity and electrocatalytic activity, which favor conjugated N-heterocyclic linkers (e.g., imidazolates, polyimines, porphyrins, and phthalocyanines) capable of π–d orbital overlap and charge delocalization—properties inaccessible to insulating carboxylate σ-bonded frameworks.^104,105^ The substantial O-donor contribution underscores the continued relevance of mature carboxylate chemistry for catalysis and separations, as well as the growing role of π-conjugated catecholate-type linkers such as 2,3,6,7,10,11-(Hexahydroxytriphenylene) (HHTP) and 2,5-Dihydroxy-1,4-benzenedicarboxylic acid (DHBDC) that bridge traditional O-coordination with electronic functionality.^106^ In contrast, S-donor systems represent a numerically minor yet functionally significant class, achieving record conductivities (e.g., Cu_3_(BHT)_2_, 1580 S cm^–1^); their limited prevalence reflects synthetic complexity and handling difficulty rather than intrinsic performance limitations.^107^

The observed linker–metal distribution reveals a chemically coherent pattern governed by fundamental coordination principles operating under application-driven constraints (Figure 13A). Hard and Soft Acid and Base (HSAB) preferences set the baseline (hard Fe^3+^ favoring O-donors, borderline metals showing balance), d-electron configurations dictate accessible geometries (square-planar Ni/Cu favoring N-donors in 2D conjugated systems), and functional demands such as conductivity systematically enrich N-donor linkers across metals.

**Figure 13:**
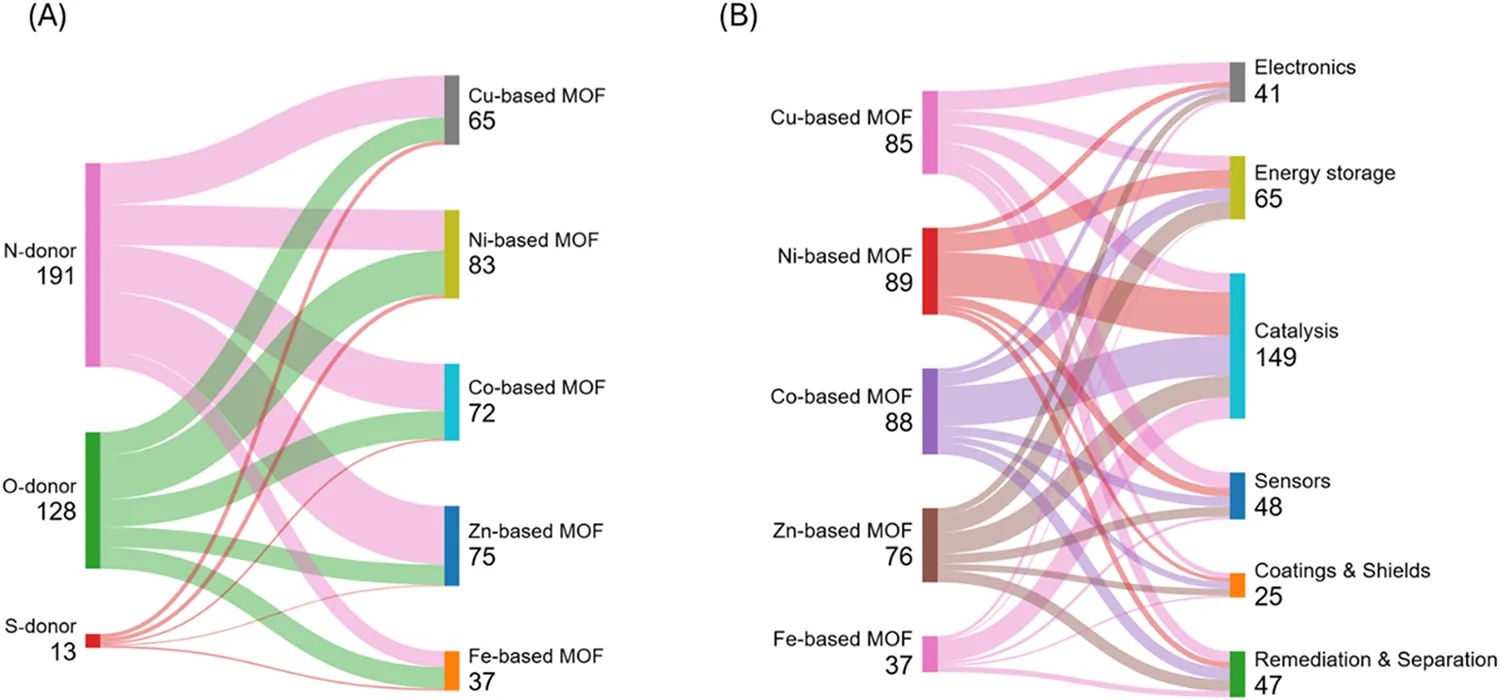
(A) Co-occurrence of linker types with various metal-based MOFs and (B) distribution of MOFs across various application areas with flow thickness indicating research volume. Data is extracted from the CAS Content Collection and includes journal articles and patent publications between 2016 to March 2026.

The predominance of catalysis as the primary application domain for 2D-MOFs, largely independent of the specific metal center, arises from the unique convergence of 2D architecture and MOF chemistry (Figure 13B).^108^ The ultrathin, layered nature of 2D-MOFs maximizes exposure of metal nodes and organic linkers, resulting in a high density of accessible active sites compared to bulk frameworks. Combined with intrinsically high surface areas (typically 1000–7000 m^2^ g^–1^)^109^ and well-defined, tunable pore environments,^110^ MOFs provide an optimal platform for heterogeneous catalysis. Their crystalline and modular nature enables precise control over coordination environments through reticular chemistry, allowing catalytic activity to be engineered at the molecular level via open metal sites, redox-active metal centers, functionalized linkers, or metal–ligand cooperativity.^111^ As a result, 2D-MOFs have been broadly applied across diverse catalytic regimes, including Lewis acid catalysis, redox catalysis,^112^ electrocatalysis,^113^ photocatalysis,^114^ and enzyme-mimetic^115^ transformations. Importantly, the reduced thickness of 2D-MOFs shortens charge and mass transport pathways, enhancing reaction kinetics and catalytic efficiency, particularly in electro- and photocatalytic processes, while ordered porosity facilitates substrate diffusion and size-selective catalysis reminiscent of enzymatic microenvironments. From a practical standpoint, catalytic performance of 2D-MOFs can be readily evaluated using established electrochemical and reactor-based methodologies, lowering experimental barriers relative to more application-specific uses such as gas storage or membrane separation. Coupled with strong societal and funding drivers in energy conversion, sustainable synthesis, and environmental remediation, these attributes collectively position catalysis as the most pervasive and universally explored functional domain for 2D-MOFs across diverse metal.^108^

Energy storage represents another major application domain for 2D MOFs, driven by the convergence of structural advantages, such as short diffusion lengths, high surface areas, and exposed redox-active sites—with their functional versatility. These materials can exhibit tunable redox chemistry and support multiple charge storage mechanisms, making them promising candidates for addressing the limitations of conventional battery materials. However, their performance is highly dependent on framework design, particularly in terms of conductivity and stability.^116^ Consequently, 2D-MOFs are consistently explored as electrode materials and functional components across a broad range of battery and supercapacitor systems.^117,118^

2D Cu-based MOFs exhibit comparable relevance in electronic applications because Cu centers possess favorable d^9^ electronic configurations and accessible Cu^+^/Cu^2^ redox couples that enable intrinsic charge carrier generation and enhanced conductivity. Strong π–d orbital overlap between copper nodes and planar conjugated ligands gives rise to extended 2D π-conjugated networks with semiconducting to near metallic behavior, reaching conductivities up to 1,580^3^ S cm^–1^ (e.g., Cu_3_(C_6_S_6_)_2_).^107^ For the same reason, Cu-based 2D MOFs function effectively as sensors that translate analyte adsorption directly into measurable electrical or optical signals.^119^ The distinct mechanisms that operate here are charge-transfer transitions, fluorescence modulation, and redox-induced spectral changes at the Cu centers.

Co- and Zn-based MOFs are frequently investigated for separation and remediation applications due to their excellent molecular transport characteristics, well-defined coordination geometries and tunable frameworks, which can contribute to favorable stability and pore architectures. However, these properties are highly system-dependent and influenced by metal–ligand chemistry and overall framework design.^120,121^

#### 3.6.2 2D COF

2D covalent organic frameworks (COFs) represent a rapidly expanding class of crystalline porous materials constructed through reversible covalent bond formation between organic building blocks.^122^ The choice of linkage type in 2D COFs is crucial, as it governs crystallinity, stability, π-conjugation, and overall framework functionality, thereby influencing their physicochemical properties and applications. Our analysis reveals a clear dominance of imine-linked COFs which have become the cornerstone of COF chemistry due to their straightforward synthesis via Schiff-base condensation between aldehydes and amines, alongside their excellent chemical stability and crystallinity (Figure 2).^123^ The reversibility of imine bond formation under solvothermal conditions enables error correction during synthesis, facilitating the formation of highly ordered layered structures with permanent porosity.^124^ Beyond imine COFs, the field has expanded to β-ketoenamine frameworks with enhanced hydrolytic stability,^125^ boronate ester/boroxine COFs characterized by high crystallinity,^126^ fully conjugated sp^2^-carbon (olefin) COFs offering exceptional chemical robustness,^127^ and azine/hydrazone COFs providing nitrogen-rich, metal-coordinating alternatives.^128,129^

The application landscape of imine-COFs reflects their structural and functional versatility (Table 1). Catalysis emerges as the most prominent application area, leveraging the high surface areas, tunable pore environments, and ease of incorporating catalytically active sites through metalation or functional group modification.^130^ Energy storage applications exploit the redox-active frameworks and ordered ion-transport channels for batteries and supercapacitors.^131^ In electronics, the π-conjugated structures and semiconducting properties of COFs are being harnessed for optoelectronic devices and chemiresistive sensors, enabling advancements in organic field-effect transistors and gas detection platforms.^132–134^ Remediation and separation applications capitalize on precise pore engineering and functional group decoration to achieve selective capture of pollutants, toxic metals, and molecular separations.^135^ Emerging applications span coatings,^136^ energy conversion^137,138^ and sensors,^134^ collectively demonstrating the remarkable adaptability of COF platforms. While ongoing efforts to diversify COF linkage chemistry aim to overcome limitations such as hydrolytic instability and limited electronic conductivity, emerging strategies, such as mixed-linkage frameworks and fully irreversible yet crystalline bonds, represent key frontiers for expanding the functional scope of 2D COFs.

**Table 1:** Distribution of 2D Imine-COF across Various Application Areas^a^

Application areas	# Docs (Imine-COF)
Catalysis	25
Energy storage	11
Electronics	5
Remediation & Separation	5
Coatings & Shields	3
Energy conversion	3
Sensors	2

a Data is extracted from the CAS Content Collection and includes journal articles and patent publications between 2016 to March 2026.

In general, 2D organic frameworks exemplify how controlled framework design can bridge porosity and functionality to unlock advanced applications, with catalysis emerging as a major application area owing to their tunable active sites, high surface areas, and precisely engineered frameworks.

### 3.7 2D Perovskites

Two-dimensional perovskites represent a structurally engineered class of layered materials where quantum-confined inorganic metal-halide octahedral sheets alternate with organic spacer cations. This architecture combines tunable optoelectronic properties with enhanced environmental stability compared to bulk counterparts, enabling applications spanning photovoltaics, light-emitting devices, transistors, and sensors through systematic compositional and structural modifications.

#### 3.7.1 Perovskites Grouped by Metal

The research landscape of 2D perovskites reveals striking differences between lead and tin-based systems, with lead perovskites dominating the field (Figure 2). However, the application distribution patterns expose fundamental property-application relationships driven by metal cation chemistry. Lead perovskites show their strongest presence in energy conversion applications, closely followed by photonics (Figure 14A). This trend correlates with lead’s superior optoelectronic properties: high absorption coefficients, optimal bandgaps (1.5–2.2 eV), long charge carrier lifetimes, and exceptional defect tolerance due to lead’s ns^2^ electronic configuration. These characteristics make Pb-based 2D perovskites ideal for photovoltaics and photodetectors, where efficient light harvesting and charge transport are paramount.^139^ Tin perovskites exhibit a dramatically different profile, with electronics and photonics leading their applications—a stark contrast to lead’s energy conversion focus. This exception reflects tin’s higher hole mobility and narrower bandgaps, making Sn-based perovskites attractive for transistor applications and near-infrared optoelectronics.^140^ The reduced energy conversion presence stems from tin’s critical weakness: the facile oxidation of Sn^2+^ to Sn^4+^, which severely compromises device stability in photovoltaic applications where long-term performance is essential.

**Figure 14:**
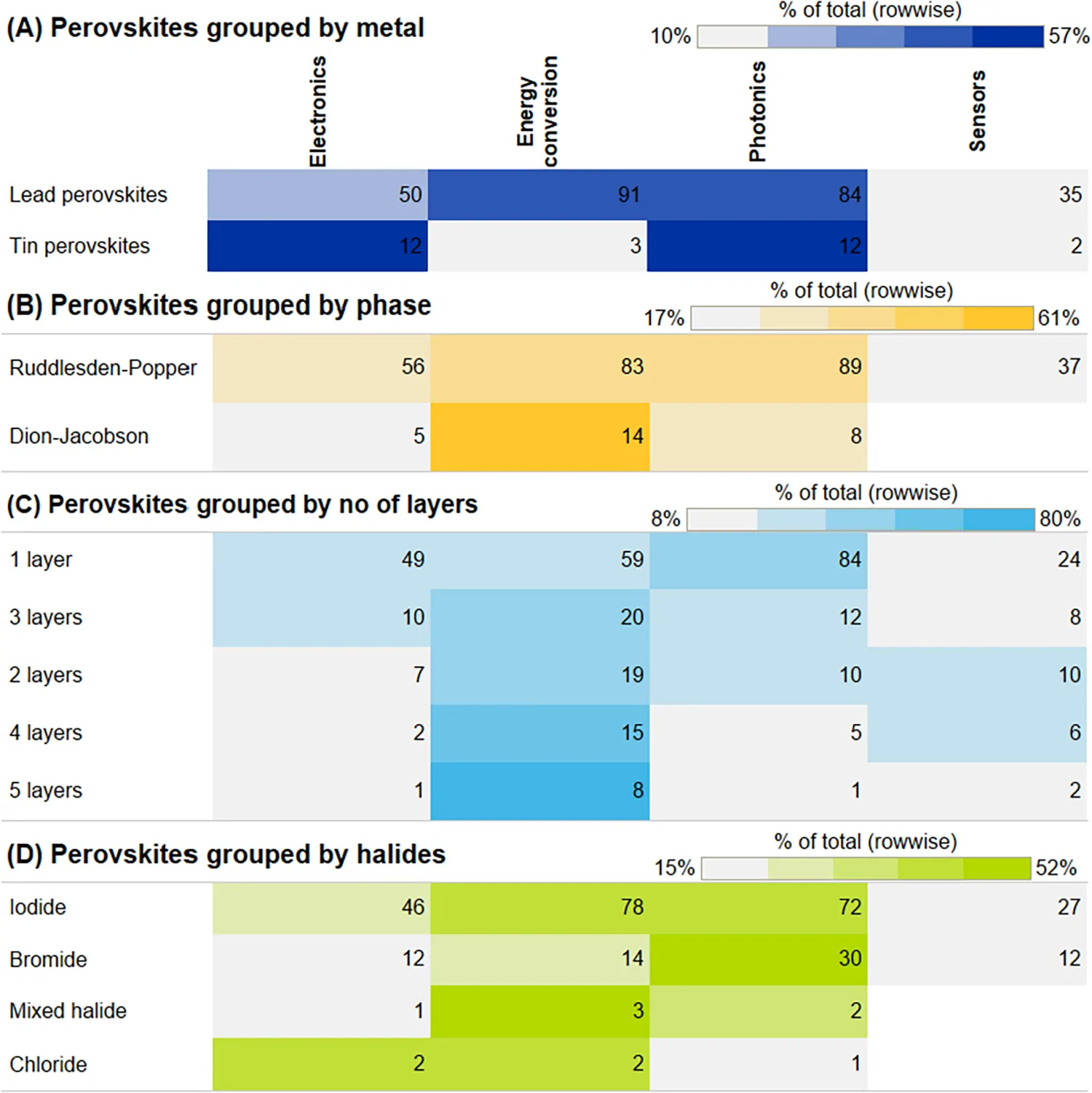
Distribution of 2D perovskite grouped using various parameters in applications: (A) grouped by metal; (B) grouped by phase; (C) grouped by number of layers; (D) grouped by halides. The cells in each row are color-coded independently according to the number of publications. Data is extracted from the CAS Content Collection and includes journal articles and patent publications between 2016 to March 2026.

#### 3.7.2 Perovskites Grouped by Crystal Phase

The structural phase of 2D perovskites profoundly influences their application focus, as evidenced by distinct trends between Ruddlesden–Popper (RP) and Dion-Jacobson (DJ) phases. While RP phases dominate the literature, the application distributions reveal critical structure–property-application relationships (Figure 14B). RP perovskites exhibit nearly equal emphasis on photonics and energy conversion, reflecting their structural advantages. The bilayer spacer cations (typically long-chain alkylammonium) in RP phases create van der Waals gaps that provide moisture resistance while maintaining reasonable charge transport perpendicular to layers. The moderate dielectric confinement from double organic layers produces strong exciton binding energies beneficial for LEDs while still allowing adequate carrier extraction for solar cells.^139^ DJ phases are focused on energy conversion compared to RP phase. This trend correlates with DJ’s unique structural feature: shorter diammonium spacers that bridge adjacent inorganic layers without intervening van der Waals gaps.^141^ This architecture produces closer layer stacking, enhancing out-of-plane charge transport crucial for photovoltaic efficiency. The improved electronic coupling in DJ phases reduces charge recombination losses, making them superior for energy conversion despite smaller publication volume.

#### 3.7.3 Perovskites Grouped by Number of Layers

The number of inorganic layers in 2D perovskites fundamentally dictates their optoelectronic properties and resulting applications, revealing a clear transition from photonics-dominated to energy conversion-focused research as layer count increases (Figure 14C). Single-layer perovskites (*n* = 1) dominate the field and show the strongest photonics emphasis. This trend reflects maximum quantum confinement effects: monolayers exhibit the largest bandgaps (>2.4 eV), strongest exciton binding energies (>300 meV), and most pronounced dielectric confinement. These properties create highly luminescent materials with narrow emission line widths ideal for LEDs, and lasers.^142^ As layer thickness increases, energy conversion applications systematically rise from 35% (*n* = 1) to 80% (*n* = 5). This progression correlates with reduced quantum confinement, reduced exciton binding energies, and improved charge carrier mobility. These characteristics favor photovoltaic applications where efficient free carrier generation and transport are critical, unlike photonics which benefit from bound excitons.

#### 3.7.4 Perovskites Grouped by Halide

Halide composition determines the optoelectronic properties of 2D perovskites, with publication trends revealing distinct application preferences driven by bandgap tuning and structural stability considerations (Figure 14D). Iodide-based perovskites show strong dual emphasis on energy conversion and photonics, reflecting their narrow bandgaps compared to other halides.^143^ Bromide perovskites exhibit a remarkable shift toward photonics with significantly reduced energy conversion focus compared to iodide. This trend correlates with bromide’s wider bandgap compared to iodides suitable for display technologies and LEDs.^140^ Mixed halide systems show elevated energy conversion emphasis, reflecting researchers’ attempts to tune bandgaps by combining halides (I/Br or Br/Cl ratios) for optimal solar cell performance.^144^ Chloride perovskites dominate in electronics, attributable to their widest bandgaps and highest formation energies, which improve thermal stability and reduce ionic migration—critical for transistor reliability despite limited optical applications due to UV-range absorption.^145^

### 3.8 Layered Double Hydroxides (LDHs)

Layered Double Hydroxides (LDH) are positively charged, sheet-like materials composed of mixed metal hydroxides, with exchangeable interlayer anions. Their general stoichiometric formula is [M_1–*x*_^2+^M_*x*_^3+^(OH)_2_]^*x*+^(A^*n*–^)_*x*/*n*_·mH_2_O. Their tunable composition, high anion exchange capacity, and unique “memory effect”, enable reconstruction of the layered structure after calcination. This makes them highly versatile for applications in catalysis, environmental remediation, and energy storage. LDHs also act as ideal precursors for layered double oxides (LDOs) because their layered structure enables atomic-level mixing of metal cations, which upon calcination yields highly uniform, porous mixed metal oxides with tunable composition and functionality.

As guided by the materials map in Figure 2, LDHs represent a significant share of 2D materials, with dominant applications in catalysis, energy storage, remediation and separation, as well as coatings and shielding (Figure 3). This section provides a closer examination of the substances contributing to the LDH category and their respective applications. The LDH category is further classified based on the number of metals present (Figure 15A), distinguishing binary, ternary, and more complex systems.

**Figure 15:**
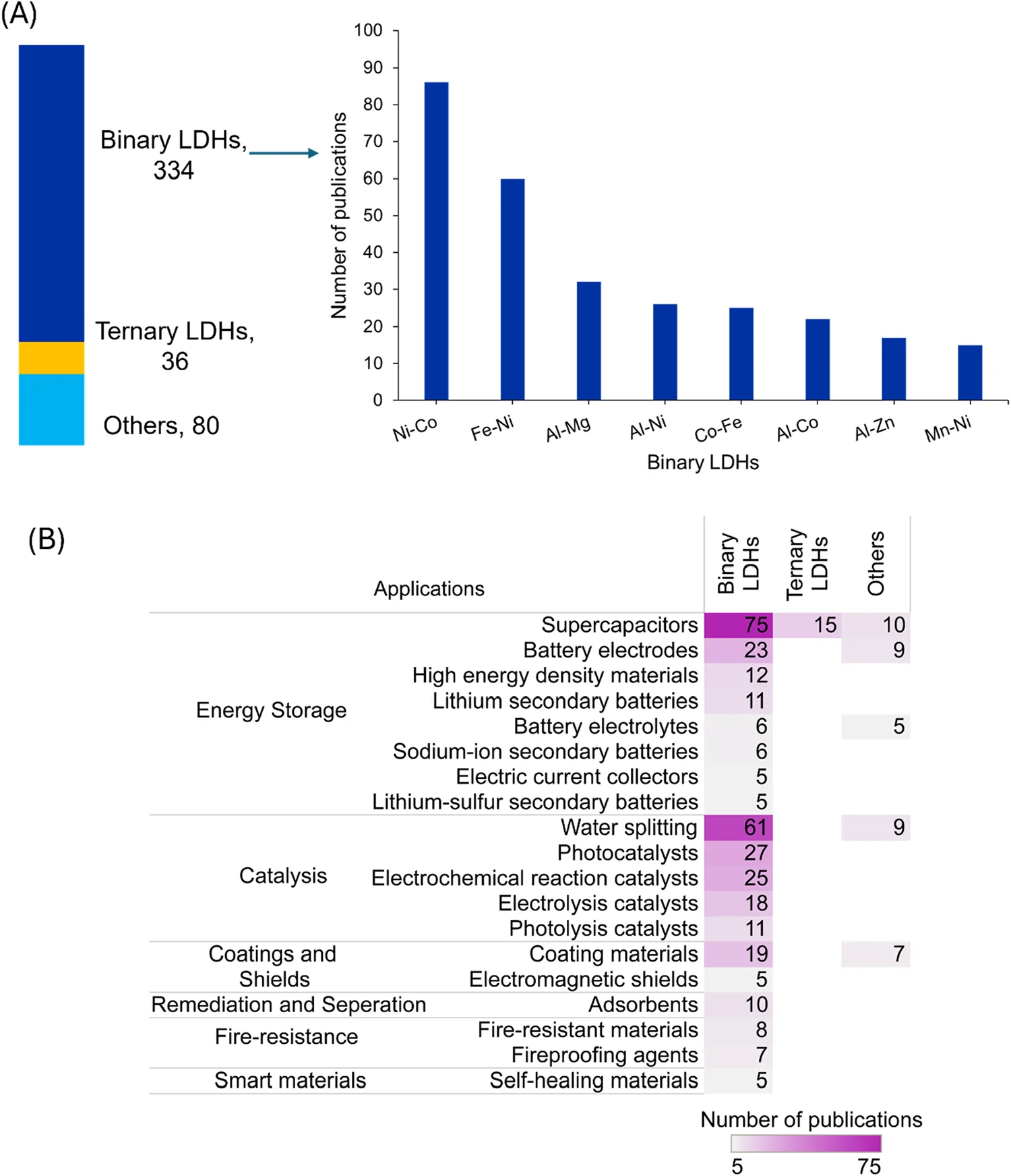
Distribution of LDHs: (A) by number of metals and binary LDHs with documents more than 10; (B) Application and LDH groups co-occurrence. Data is extracted from the CAS Content Collection and includes journal articles and patent publications between 2016 to March 2026.

Binary LDHs, dominate due to their ease of synthesis, cost-effectiveness, and well-established structure–property relationships. They serve as reliable workhorse materials across catalysis, energy storage, and coatings, but their performance has begun to saturate due to limited compositional flexibility.^146^ The most reported systems are Ni–Co, Fe–Ni LDHs, which are widely used in electrochemical energy storage and oxygen evolution reactions due to their strong redox activity.^147,148^ However, their performance is increasingly limited by restricted compositional tunability. Secondary systems such as Al–Mg, Al–Ni, and Co–Fe shows moderate representation, play roles in structural stabilization and functional tuning. Al-based LDHs are additionally used in coatings as corrosion inhibitors and in flame-retardant applications due to controlled release and thermal decomposition behavior.^149,150^ In contrast, systems like Al–Zn and Mn–Ni appear less frequently, suggesting more niche or emerging roles in LDH research.

The distribution of metals in binary LDH compositions highlights a property-driven hierarchy. Ni and Co dominate due to their multiple oxidation states and rich redox activity, which enhance carrier transfer and catalytic performance.^151,152^ Ni shows particularly stable and reversible redox transitions, while Co contributes higher electronic conductivity and facilitates faster charge transport due to its more delocalized electronic structure.

Al and Fe form another dominant group but play fundamentally different roles.^153^ Al stabilizes the LDH lattice due to its small ionic radius and strong metal–oxygen bonding, which tightens the structure and improves robustness.^146,154^ Fe is used to modify the electronic structure of neighboring metals and stabilize higher oxidation states through strong metal–metal interactions within the lattice.^155^

The remaining metals contribute more specialized properties. Mg enhances structural stability and interlayer spacing, while Zn provides moderate redox activity and stable coordination. Mn, with multiple oxidation states, can improve charge storage but may introduce structural instability. Cu offers distinct electronic characteristics for selective electron transfer.^155,156^

Ternary LDHs introduce a third metal, enabling synergistic tuning of activity, conductivity, and stability, often resulting in improved performance. Their adoption, however, is constrained by synthesis complexity, phase control challenges, and higher costs. Despite these barriers, ternary LDHs are rapidly gaining attention as they offer a pathway to overcome the intrinsic limitations of binary systems.^157^ More complex systems (others group), including multimetal and modified LDHs, reflect growing efforts toward engineered structures with enhanced functionality. Increased attention is directed toward ternary, quaternary, and multicomponent LDHs, where the incorporation of additional metals (e.g., Fe, Mn) introduces synergistic effects, improves electronic structure, and increases active site density, thereby enhancing catalytic and electrochemical performance.^158,159^

Figure 15B shows the LDHs type across different applications areas, showing a clear dominance of binary LDHs, particularly in energy storage and catalytic processes. Binary systems like Ni–Co-based LDHs dominate energy-driven applications, especially electrochemical energy storage and conversion.^146,160,161^ In comparison, ternary LDHs appear in smaller but significant numbers, mainly in advanced applications like supercapacitors and catalysis, where additional metal components are introduced to fine-tune the electronic structure and improve performance.^157,162^ The others category, which includes multimetal (>3) systems, modified LDHs, and LDH-derived materials, shows a more distributed presence across applications. These systems are more common in areas such as coatings and specialty energy systems, reflecting their broader compositional diversity and structural modifications.^163^

### 3.9 Layered Clay Minerals

Clays are naturally occurring layered aluminosilicate minerals belonging to the broader class of 2D materials, characterized by stacked sheet-like structures held together by weak interlayer forces. These layers can be exfoliated into nanosheets, imparting high surface area and unique interfacial properties.^164^

As shown in Figure 2, clays form a distinct class within the 2D material landscape. Despite a relatively smaller publication volume, they play important roles in applications such as adsorption, catalysis, nanocomposites, and environmental remediation, driven by their swelling behavior, ion-exchange capacity, and surface reactivity.

#### 3.9.1 Structural Classification and Applications

The structural grouping in Figure 16A classifies clay minerals based on the arrangement of their tetrahedral (T) and octahedral (O) sheets, which governs properties such as swelling, ion exchange, and adsorption. 2:1 (TOT) clays (e.g., montmorillonite, nontronite, hectorite, and vermiculite) consist of an octahedral sheet between two tetrahedral layers. Variations in layer charge determine their behavior, with low charge leading to swelling clays (montmorillonite) and higher charge resulting in limited-swelling structures (vermiculite). In contrast, 1:1 (TO) clays (kaolinite and halloysite) are nonswelling and structurally stable, while fibrous silicates (e.g., palygorskite and sepiolite) possess chain-like architectures that result in distinct porosity and adsorption characteristics.^164^ These structural differences underpin their functional roles in catalysis, adsorption, and nanocomposites.

**Figure 16:**
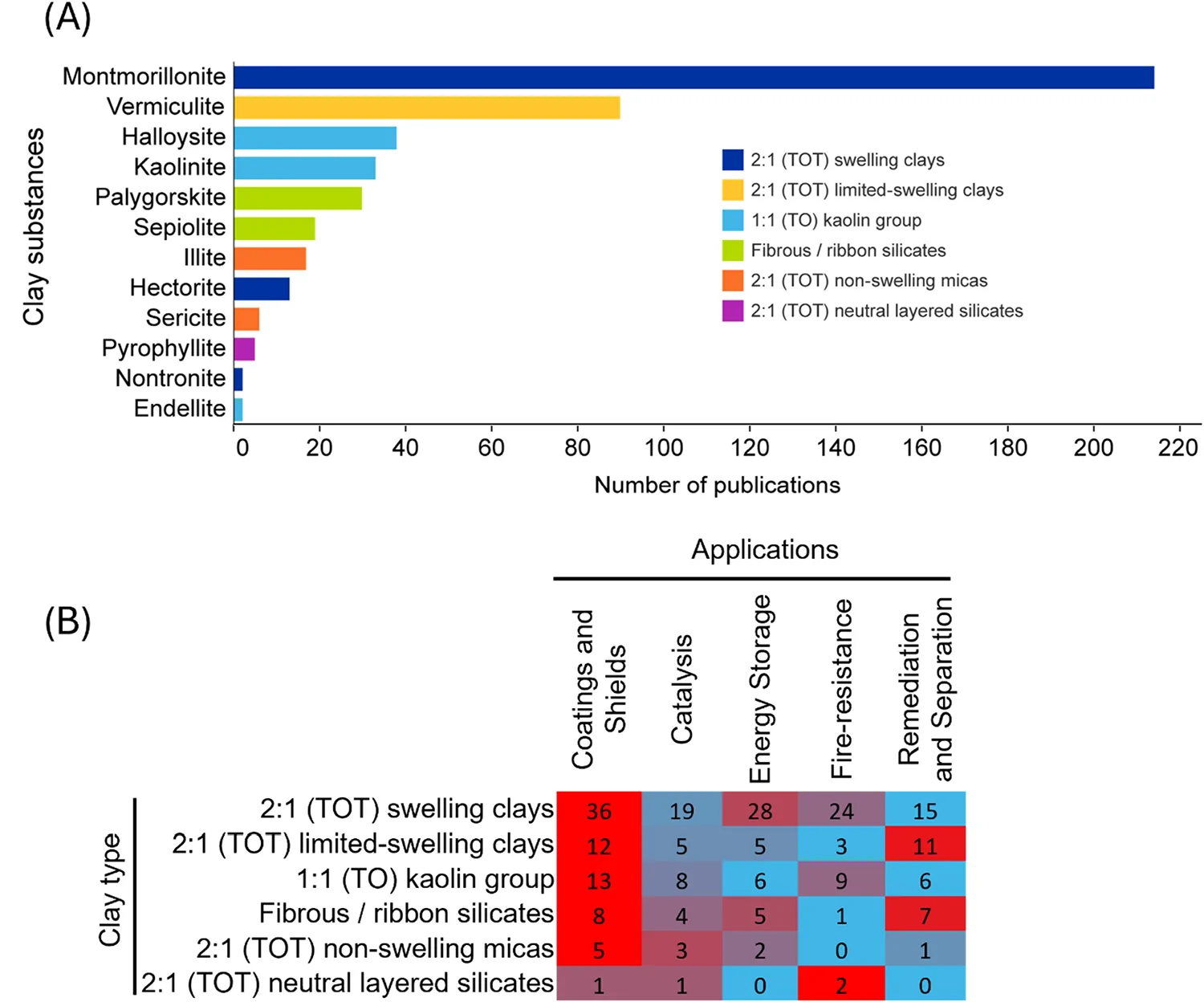
(A) Publication volume of clay mineral substances color coded based on their structural group; (B) Co-occurrence of applications and clay group. Data is extracted from the CAS Content Collection and includes journal articles and patent publications between 2016 to March 2026.

Figure 16B shows the application distribution of the clay groups, closely aligned with their structural functionality. 2:1 (TOT) swelling clays dominate both research and applications due to their expandable interlayers, which enable efficient ion exchange, high-surface accessibility, and tunable interactions. Montmorillonite leads due to its high swelling capacity, large surface area, and strong ion-exchange capability, enabling efficient delamination and functionalization.^165^ Vermiculite offers similar but more controlled swelling behavior, allowing tunable interlayer spacing and enhanced structural stability for applications in membranes, catalysis, and energy storage applications.

These properties directly translate into application trends, with swelling clays dominating catalysis, energy storage, coatings, and fire resistance, where interlayer accessibility supports charge transfer, ion transport, and barrier formation.^166^ Coatings and shielding represent the most mature application domain, as layered structures provide effective barriers against heat, gases, and moisture,^167–169^ while limited-swelling clays such as vermiculite offer additional advantages in maintaining structural integrity in protective systems.^170^

In contrast, 1:1­(TO) clays (kaolin group) exhibit moderate research interest due to their strong interlayer bonding, which limits swelling, although halloysite provides niche advantages through its tubular morphology.^164,171,172^ Fibrous clays such as palygorskite and sepiolite occupy specialized roles driven by their high surface area, porosity, and adsorption capacity rather than true 2D layer exfoliation, leading to more application-specific but limited research activity.^173^

Meanwhile, non- or limited-swelling clay types exhibit minimal application, as the absence of interlayer expansion restricts ion mobility and surface accessibility, limiting their effectiveness in most advanced functional applications.^174^ Overall, the dataset reveals that swelling ability acts as the primary determinant of research intensity, creating a strong bias toward smectite-type clays while leaving nonswelling and structurally rigid clays relatively underexplored, despite their potential for future innovation through modification or hybridization.

### 3.10 2D Transition Metal Oxides

2D transition metal oxides­(TMO) represent a critical subset of layered inorganic materials that bridge the gap between traditional metal oxide chemistry and emerging 2D nanomaterials.^8,175^ The literature analysis reveals distinct research priorities across different metal oxide systems, with Mo-based oxides dominating the field (∼60%), followed by Mn-based, V-based, and W-based oxides (Figure 2). This distribution reflects not only the intrinsic properties of these materials but also their technological readiness and application potential across energy storage, catalysis, and electronic devices.

Molybdenum oxides—especially MoO_3_—are the most studied 2D metal oxides due to the layered α-MoO_3_ structure, which enables facile exfoliation into ultrathin nanosheets.^176^ Their prominence is driven by well-established synthesis routes (liquid-phase exfoliation, CVD, hydrothermal methods), multiple redox states, and strong optoelectronic and electrochromic properties, with research largely focused on photonics and electronics.^177,178^ Sensing is another key area, where 2D MoO_3_ nanosheets enable sensitive gas detection (e.g., NH_3_, NO_2_, volatile organic compounds) via surface charge transfer.^179^ Energy storage and catalysis, though smaller fields, demonstrate their versatility through supercapacitor performance^180^ and hydrogen and oxygen evolution reactivity^181^ and emerging work in coatings highlights barrier performance.^182^

Mn-based oxides form an important segment of 2D metal oxide research, mainly driven by their strong performance in energy storage. Among their polymorphs, layered δ-MnO_2_ (birnessite) is most studied due to its natural layered structure, which enables easy exfoliation into nanosheets.^183^ Their appeal lies in high pseudocapacitance from reversible redox reactions, low cost and toxicity, multiple oxidation states (Mn^2+^/Mn^3+^/Mn^4+^) enabling versatile chemistry, and effective catalytic activity in oxygen reduction reaction^184^ and pollutant degradation.^185^ Compared to Mo-oxides, their lower research volume reflects synthesis challenges and a narrower focus on supercapacitors and batteries. However, advances in chemical exfoliation methods are improving scalability and may broaden their application scope.

V-based oxides occupy a niche in 2D metal oxide research, dominated by electronics applications. This is driven by VO_2_’s metal–insulator transition (MIT) with large resistivity changes (10^3^–10^5^ fold),^186^ enabling smart windows,^187^ optical switches, and neuromorphic devices.^188^ V_2_O_5_ nanosheets serve as p-type semiconductors,^189^ hole transport layers in solar cells,^190^ and active materials in memristors.^191^ They also exhibit electrochromic behavior and high photoresponsivity.^192^ Although used in energy storage, their unique MIT and optoelectronic features distinguish them from Mn-based (energy-focused) and Mo-based (photonics-focused) oxides.

W-based oxides are the least explored 2D TMOs, limited by higher mass, synthesis challenges, cost, and overlap with MoO_3_. However, W-based oxides demonstrate notable conductivity along with applications in 2D photodetectors, visible-light photocatalysis, and gas sensing.^193^ Their low research volume reflects underexplored potential rather than limited capability.

Overall, 2D transition metal oxides demonstrate diverse yet complementary functionalities, where their structure–property relationships govern distinct application niches across electronics, energy storage, catalysis, and sensing, highlighting both their technological maturity and untapped potential.

### 3.11 Transition-Metal Carbides, Nitrides, and Borides (non-MXenes)

While MXenes have dominated the field of layered transition-metal compounds, parallel efforts have established non-MXene 2D transition-metal carbides (TMCs), nitrides (TMNs), and borides (TMBs) with diverse stoichiometries (MX, M_2_X, MX_2_: X = C/N/B) that extend beyond the MXenes (M_*n*+1_X*_n_* T_*x*_) framework.^194^ This compositional diversity enables electronic, catalytic, and mechanical properties inaccessible to conventional MXenes.^194^ Unlike carbide MXenes, which rely on hazardous HF etching and often carry surface terminations that modify intrinsic properties,^25^ non-MXene 2D TMCs, TMNs and TMBs can be synthesized via chemical exfoliation of layered precursors,^195^ bottom-up chemical vapor deposition (CVD),^196^ plasma-enhanced atomic layer deposition (PEALD),^197^ or salt-templated routes,^198^ yielding termination-free or compositionally tunable phases inaccessible within the MXene framework.

Our data set reveals the presence of non-MXene transition metal (TM)–based carbides, nitrides, and borides as presented in Figure 2. Electrocatalysis dominates non-MXene 2D TMCs, TMNs and TMBs, with Mo_2_C/W_2_C enabling Pt-like hydrogen evolution reaction,^199^ Fe_3_C/Co_3_C and metal nitrides driving oxygen reduction reaction,^200,201^ and Mo–C/B promoting CO_2_ reduction.^201,202^ Beyond catalysis, VN and Mo_2_N deliver high pseudocapacitance (up to 1500 F g^–1^),^203^ Fe-based carbides/nitrides provide effective EMI shielding (>40 dB),^204,205^ and ultrahigh-κ boride monolayers support advanced thermal management,^206^ collectively highlighting their multifunctional energy and electronic utility.

These carbides (TMCs), nitrides (TMNs), and borides (TMBs) face key challenges, including polymorph control, air and moisture sensitivity, limited scalability beyond laboratory synthesis, which collectively restrict their presence in current data sets. Nevertheless, their exceptional compositional and structural diversity, pristine surfaces, and tunable stoichiometries enable multifunctional properties spanning catalysis, energy storage, and electronic applications, positioning them as promising platforms for next-generation integrated 2D material technologies.

### 3.12 2D Mica Minerals

Mica minerals belong to the phyllosilicate group, characterized by layered crystal structures comprising strong in-plane covalent bonding within tetrahedral–octahedral–tetrahedral (TOT) sheets and weak interlayer van der Waals interactions. Crystal Structures of Clay Minerals and their X-ray Identification^207^ This anisotropic bonding facilitates facile cleavage and exfoliation into atomically thin layers (1 nm–<10 nm), making mica an attractive candidate for 2D materials research.^208,209^ Analysis of our data set indicates that fluorophlogopite (KMg_3_(AlSi_3_O_10_)­F_2_)^210^ and muscovite (KAl_2_(AlSi_3_O_10_)­(OH)_2_) are the most prevalent mica-based 2D materials, primarily owing to their enhanced structural stability, ease of exfoliation, and availability in high-purity synthetic or natural forms (Figure 2).

The appeal of 2D mica arises from its unique combination of physicochemical properties. Phyllosilicate micas exhibit ultrasmooth, atomically flat surfaces with root-mean-square roughness below ∼0.2 nm, making them ideal substrates for assembling defect-free van der Waals heterostructures.^208^ They possess wide bandgaps (>7 eV), enabling high optical transparency (>90% for monolayers), and exhibit excellent dielectric properties, with relative permittivity (ε_r_ ∼ 6–7 for muscovite) and high breakdown strengths (>10 MV/cm), supporting dielectric scaling in nanoelectronics. In addition, mica nanosheets demonstrate exceptional thermal stability (up to ∼800–900 °C for muscovite and ∼1000 °C for fluorophlogopite) and strong chemical inertness toward acids, bases, and organic solvents.^210,211^

Despite the intrinsic brittleness of bulk mica, ultrathin sheets exhibit sufficient flexibility to conform to substrates without fracture, enabling integration into flexible and stretchable electronic systems. These combined attributes position 2D mica as a versatile platform for advanced applications in nanoelectronics and optoelectronics, where ultrathin mica layers have been demonstrated as effective gate dielectrics in field-effect transistors, offering high breakdown strength and low leakage currents.^212,213^

Beyond electronics, 2D mica also shows promise in coatings and composites. Exfoliated mica fillers and nanosheets act as high-aspect-ratio platelets that create tortuous diffusion pathways, significantly reducing permeability.^214,215^ While this mechanism is well established for layered silicates and mica flakes, it is expected to be further enhanced in fully exfoliated 2D mica nanosheets due to improved dispersion and higher aspect ratio.^216,217^

Furthermore, emerging tribological applications highlight the potential of 2D mica as a lubricant additive. Incorporation of 0.2 wt % 2D mica into engine oil has been shown to significantly reduce wear rate (∼72%) and lower the coefficient of friction (∼28%) under pure sliding conditions.^218^

Despite these advantages, 2D mica faces limitations that hinder broader adoption. Its wide bandgap restricts it to passive roles (substrates, dielectrics), while scalable exfoliation methods yield submicron flakes, limiting large-area integration. Additional challenges include compositional variability, surface charging effects, and strong anisotropy, which complicate device performance and processing.

Ongoing research aims to address these issues through surface functionalization,^219,220^ heterostructure integration with conductive 2D materials,^212^ and hybrid composites incorporating nanoparticles.^221^ Advances in scalable exfoliation^222^ and computational design are further enabling controlled synthesis and property tuning.^223^

Collectively, while 2D mica lacks the electronic versatility of graphene or TMDs, its stability, transparency, chemical inertness, and low-cost, earth-abundant nature position it as a promising material for flexible electronics, dielectrics, coatings, and barrier applications, particularly where scalability and durability are critical.

## 4 Strengths and Limitations of the Data-Driven Approach

The data-driven approach used in this review complements traditional expert reviews by enabling quantitative comparisons across 2D-material classes, revealing broad shifts in research activity and application focus, and highlighting emerging or comparatively less explored areas. The inclusion of both journal and patent literature provides perspectives on scientific research and application-oriented activity. Publication frequency and material–application co-occurrence provide robust measures of patterns within the indexed literature, but represent research attention and documented associations rather than direct measures of material performance, technological readiness, or causality. These patterns are therefore interpreted alongside expert discussion of material composition, structure, properties, and relevant scientific evidence. As with any literature-based analysis, the results depend on the defined search strategy, classification framework, indexing practices, and analysis period, while trends in small categories may be sensitive to limited document counts. Accordingly, the taxonomy and interpretations presented here were developed with domain expertise. The resulting landscape is intended to identify and prioritize research directions, while focused experimental and theoretical studies provide the evidence needed to evaluate mechanisms and performance.

## 5 Summary and Outlook

This comprehensive analysis of over 66 thousand publications published between 2016 and 2026 reveals the rapidly expanding landscape of nongraphene 2D materials, dominated by MXenes (metallic conductors with hydrophilicity), followed by Xenes (elemental variants like phosphorene), metal chalcogenides, and diverse emerging families including metal-free nitrides, metal halides, organic frameworks (MOFs/COFs), perovskites, and layered materials (LDHs, clays). Publication volume surged nearly 10-fold, with journal articles comprising 81% and patents 19%, reflecting strong academic momentum alongside commercial translation.

This data-driven analysis establishes that the nongraphene 2D materials landscape is governed by fundamental structure–property-application relationships, where electronic structure (bandgap, conductivity, magnetic properties), surface chemistry (terminations, active sites, porosity), and structural architecture (layer thickness, stoichiometry, phase) systematically dictate functional specialization. The field is transitioning from materials discovery to application-driven compositional engineering, evidenced by explosive growth in hybrid systems (Janus TMDs, ternary LDHs, mixed-halide perovskites, tunable MXene terminations) that overcome single-phase limitations through synergistic effects. Clear application clusters emerge: redox-active transition metal systems (MXenes, Ni/Co-LDHs, TMD alloys) are mostly applied in energy storage and catalysis leveraging multiple oxidation states and high surface areas; semiconducting materials with tunable bandgaps (Mo/W-TMDs, 2D perovskites, Xenes) find applications in electronics and photonics; layered barrier properties (clays, h-BN, Al-LDHs) are exploited in coatings and shielding; and specialized functions like magnetism (Cr-halides, Fe-TMDs) and photocatalysis (g-C_3_N_4_, BiO_X_) depend on intrinsic electronic/optical characteristics.

Opportunities include underexplored families (mica minerals for large-area flexible substrates, non-MXene carbides for termination-free catalysis, p-block chalcogenides for topological applications), scalability challenges in air-sensitive synthesis (Sn-perovskites, S-terminated MXenes) and phase control (TMD polymorphs), and the need for computational guidance in high-dimensional compositional spaces (high-entropy systems, multicomponent alloys). Composite and heterostructure literature represents a promising avenue for future data-driven investigations, as much application-oriented progress now occurs in this area. Stratified statistical assessment by matrix type, filler role, and hybrid architecture would capture this expanding design space and more accurately elucidate trends for individual 2D materials. The convergence of catalysis, energy storage, and flexible electronics—driven by decarbonization, electrification, and wearable technology—positions nongraphene 2D materials as indispensable platforms where rational design, guided by the quantitative trends revealed here, will accelerate translation from laboratory discovery to technological deployment.

## Supplementary Material



## Notes

Brief description of the methods used, including the search strategy, data scope, and data analysis; List of CAS indexed concepts grouped under the application categories is provided in Table S1; List of substances under various substance groups in the individual sections is provided in Tables S2–S11 (PDF)
