# Supplementary material for: Non-Graphene Two-Dimensional Materials: Structure–Property–Application Relationships from a Curated Data Analysis
Source: ACS Mater Au. 2026 Sep 29;6(6):1247–72. doi: 10.1021/acsmaterialsau.6c00190 (PMC13760663; doi:10.1021/acsmaterialsau.6c00190)
Supplement: Supplementary file 1 [file mg6c00190_si_001.pdf]

Supporting information for

Non-Graphene Two-Dimensional Materials: Structure–Property–Application Relationships from a Curated Data Analysis

Magesh Ganesan[1†], Bhuvaneshwari S Baranwal[1†], Suparna Roy[1†], Qiongqiong Angela Zhou*[2]

[1]ACS International India, No 23, Baner One, Near Baner-Pancard Club Road, Pune, 411045, Maharashtra, India

[2]CAS, a division of the American Chemical Society, 2540 Olentangy River Road, Columbus, Ohio 43202, United States

[†]These authors contributed equally to this article

Methods:

Search query: A search query was developed using CAS-indexed Concepts related to 2D materials alongside a curated list of 2D material substances. CAS Concepts are controlled vocabulary terms that represent standardized scientific terminology indexed from the scientific literature. All documents containing these concepts and/or substances were identified and further refined to include only patent families and journal articles published from 2016 onward.

Data extraction: Data was extracted from the CAS Content Collection for all documents identified through the search query. The extracted data included titles, abstracts, keywords, publication years, patent claims, CAS-indexed Concepts, CAS-indexed Substances, and substance roles.

Data analysis: Following data extraction, the documents were classified into various 2D material classes using multiple approaches, including substance-name matching and keyword/phrase searches within titles, abstracts, keywords, CAS Concepts, and patent claims. A similar strategy was used to group the 2D material classes into finer subcategories to enable deeper analysis. Applications reported in the documents were identified using CAS-indexed Concepts, which were then grouped into major application categories.

Data visualization: Data visualization was carried out using Tableau, Microsoft Excel, and Adobe Illustrator.

Table S1: List of CAS Lexicon concepts grouped into application

**Application**	**Concept**
Electronics	Field effect transistors
	Semiconductor devices
	Electronics

	Wearable devices
	Transistors
	Capacitors
	Superconductors
	Integrated circuits
	Electric apparatus
	Nanoelectronic devices
	Electronic devices
	Dielectric films
	Topological insulators
	Quantum well devices
	Memory devices
	Memristors
	CMOS devices
	MOSFET (transistors)
	Diodes
	Thin film transistors
	Nonvolatile memory devices
	Integrated circuits, gates
	Quantum devices
	Printed circuit boards
	Logic circuits
Textiles	Textiles
	Cotton textiles
	Nonwoven fabrics
Mechatronics	Actuators
	Robotics
Lubricants	Lubricants
Magnetic & spintronics	Spintronics
	Spintronic devices
	Ferromagnetic materials
	Two-dimensional magnetic materials
	Magnetic materials
	Antiferromagnetic materials
	Magnets
	Magnetic semiconductor materials
	Multiferroic materials
	Ferromagnetic semiconductor materials
	Magnetic nanoparticles
Catalysis	Photocatalysts
	Hydrogen evolution reaction catalysts
	Electrochemical reaction catalysts
	Catalysts

	Oxygen evolution reaction catalysts
	Water splitting catalysts
	Electrochemical reduction catalysts
	Photolysis catalysts
	Electrolysis catalysts
	Catalyst supports
	Single-atom catalysts
	Polymerization catalysts
	Nanocatalysts
	Electrochemical oxidation catalysts
	Photochemical reduction catalysts
	Nanozymes
	Reduction catalysts
	Oxidation catalysts
	Bifunctional catalysts
Remediation & Separation	Membranes, nonbiological
	Adsorbents
	Composite membranes
	Nanofiltration membranes
	Gas separation membranes
	Membrane filters
	Desalination membranes
Energy storage	Battery anodes
	Supercapacitors
	Lithium secondary batteries
	Battery cathodes
	Supercapacitor electrodes
	Sodium-ion secondary batteries
	Energy storage systems
	Lithium-ion secondary batteries
	Battery electrodes
	High energy density materials
	Batteries
	Secondary batteries
	Lithium-sulfur secondary batteries
	Phase change materials
	Hydrogen storage materials
	Potassium-ion secondary batteries
	Zinc-ion secondary batteries
	Battery electrolytes
	Electric current collectors
	Solid electrolytes
	Secondary battery separators

Coatings & Shields	Electromagnetic shields
	Coating materials
	Absorbents
	Anticorrosive coating materials
	Thermal conductors
	Thermal insulators
	Inks
	Antifoaming agents
	Antifouling agents
Energy conversion	Solar cells
	Perovskite-type solar cells
	Photoelectric devices
	Thermoelectric materials
	Triboelectric nanogenerators
	Fuel cells
	Thermoelectric devices
	Photoanodes
	Proton exchange membrane fuel cells
	Evaporators
Biomedical	Photothermal agents
	Antibacterial agents
	Photothermal therapy
	Antitumor agents
	Pharmaceutical hydrogels
	Pharmaceutical nanoparticles
	Antimicrobial agents
	Drug delivery systems
	Pharmaceutical nanocomposites
	Anti-inflammatory agents
	Tissue engineering
	Antibacterial materials
Sensors	Sensors
	Optical detectors
	Gas sensors
	Biosensors
	Electrochemical sensors
	Strain sensors
	Pressure sensors
	Wearable sensors
	Electrochemical biosensors
	Electronic skin
	Optical sensors
	IR detectors

	Temperature sensors
	Hygrometers
	UV detectors
	Motion sensors
	Immunosensors
	Aptasensors
	Electrochemical gas sensors
Photonics	Optoelectronics
	Optoelectronic apparatus
	Lasers
	Electroluminescent devices
	Photonics
	Photonic devices
	Phototransistors
	Saturable optical absorbers
	Femtosecond lasers
	Photodiodes
	Plasmon devices
	Optoelectronic semiconductor devices
	Optical resonators
	Organic electroluminescent devices
	Quantum dot devices
	Electroluminescent display devices
Fire-resistance	Fire-resistant materials
	Fireproofing agents
Smart materials	Self-healing materials
	Biomimetics
	Smart materials
	Piezoelectric materials

Table S2: **List of MXenes substances grouped by various parameters**

Table S2A: **MXenes grouped by stoichiometry**

**MXene stoichiometry group**	**Substance Name**
M2X	Chromium carbide (Cr2C)
M2X	Chromium carbide fluoride (Cr2CF2)
M2X	Chromium carbide oxide (Cr2CO2)
M2X	Chromium nitride (Cr2N)
M2X	Chromium nitride oxide (Cr2NO2)
M2X	Hafnium carbide (Hf2C)
M2X	Hafnium carbide oxide (Hf2CO2)
M2X	Hafnium nitride (Hf2N)
M2X	Molybdenum carbide (Mo2C)

M2X	Molybdenum carbide fluoride (Mo2CF2)
M2X	Molybdenum carbide oxide (Mo2CO2)
M2X	Molybdenum carbide sulfide (Mo2CS2)
M2X	Molybdenum nitride (Mo2N)
M2X	Molybdenum nitride oxide (Mo2NO2)
M2X	Niobium carbide (Nb2C)
M2X	Niobium carbide fluoride (Nb2CF2)
M2X	Niobium carbide hydroxide (Nb2C(OH)2)
M2X	Niobium carbide oxide (Nb2CO2)
M2X	Niobium carbide sulfide (Nb2CS2)
M2X	Niobium nitride (Nb2N)
M2X	Scandium carbide (Sc2C)
M2X	Scandium carbide fluoride (Sc2CF2)
M2X	Scandium carbide hydroxide (Sc2C(OH)2)
M2X	Scandium carbide oxide (Sc2CO2)
M2X	Scandium nitride (Sc2N)
M2X	Tantalum carbide (Ta2C)
M2X	Tantalum carbide oxide (Ta2CO2)
M2X	Titanium carbide (Ti2C)
M2X	Titanium carbide chloride (Ti2CCl2)
M2X	Titanium carbide fluoride (Ti2CF2)
M2X	Titanium carbide hydroxide (Ti2C(OH)2)
M2X	Titanium carbide oxide (Ti2CO2)
M2X	Titanium nitride (Ti2N)
M2X	Titanium nitride oxide (Ti2NO2)
M2X	Tungsten carbide (W2C)
M2X	Tungsten carbide oxide (W2CO2)
M2X	Vanadium carbide (V2C)
M2X	Vanadium carbide fluoride (V2CF2)
M2X	Vanadium carbide hydroxide (V2C(OH)2)
M2X	Vanadium carbide oxide (V2CO2)
M2X	Vanadium nitride (V2N)
M2X	Vanadium nitride oxide (V2NO2)
M2X	Yttrium carbide fluoride (Y2CF2)
M2X	Yttrium carbide oxide (Y2CO2)
M2X	Zirconium carbide (Zr2C)
M2X	Zirconium carbide fluoride (Zr2CF2)
M2X	Zirconium carbide hydroxide (Zr2C(OH)2)
M2X	Zirconium carbide oxide (Zr2CO2)
M2X	Zirconium nitride (Zr2N)
M3X2	Chromium titanium carbide (Cr2TiC2)
M3X2	Chromium vanadium carbide (Cr1.5V1.5C2)
M3X2	Hafnium carbide (Hf3C2)

M3X2	Hafnium carbide oxide (Hf3C2O2)
M3X2	Molybdenum carbide (Mo3C2)
M3X2	Molybdenum scandium carbide (Mo2ScC2)
M3X2	Molybdenum titanium carbide (Mo2TiC2)
M3X2	Molybdenum titanium carbide oxide (Mo2TiC2O2)
M3X2	Molybdenum vanadium carbide (Mo2VC2)
M3X2	Niobium carbide (Nb3C2)
M3X2	Niobium carbide oxide (Nb3C2O2)
M3X2	Tantalum carbide (Ta3C2)
M3X2	Titanium bromide carbide (Ti3Br2C2)
M3X2	Titanium carbide (Ti3C2)
M3X2	Titanium carbide chloride (Ti3C2Cl2)
M3X2	Titanium carbide fluoride (Ti3C2F2)
M3X2	Titanium carbide fluoride (Ti3C2F)
M3X2	Titanium carbide hydroxide (Ti3C2(OH)2)
M3X2	Titanium carbide hydroxide (Ti3C2(OH))
M3X2	Titanium carbide iodide (Ti3C2I2)
M3X2	Titanium carbide nitride (Ti3CN)
M3X2	Titanium carbide oxide (Ti3C2O2)
M3X2	Titanium carbide oxide (Ti3C2O)
M3X2	Titanium carbide sulfide (Ti3C2S2)
M3X2	Titanium nitride (Ti3N2)
M3X2	Vanadium carbide (V3C2)
M3X2	Vanadium carbide oxide (V3C2O2)
M3X2	Zirconium carbide (Zr3C2)
M3X2	Zirconium carbide oxide (Zr3C2O2)
M4X3	Molybdenum carbide (Mo4C3)
M4X3	Molybdenum titanium carbide (Mo2Ti2C3)
M4X3	Niobium carbide (Nb4C3)
M4X3	Tantalum carbide (Ta4C3)
M4X3	Titanium carbide oxide (Ti4C3O2)
M4X3	Vanadium carbide (V4C3)
**Table S2B: MXenes grouped by transition metals**	
**MXene transition metal group**	**Substance Name**
Cr MXenes	Chromium carbide (Cr2C)
Cr MXenes	Chromium carbide fluoride (Cr2CF2)
Cr MXenes	Chromium carbide oxide (Cr2CO2)
Cr MXenes	Chromium nitride (Cr2N)
Cr MXenes	Chromium nitride oxide (Cr2NO2)
Hf MXenes	Hafnium carbide (Hf2C)
Hf MXenes	Hafnium carbide (Hf3C2)
Hf MXenes	Hafnium carbide oxide (Hf2CO2)

Hf MXenes	Hafnium carbide oxide (Hf3C2O2)
Hf MXenes	Hafnium nitride (Hf2N)
Mo MXenes	Molybdenum carbide (Mo2C)
Mo MXenes	Molybdenum carbide (Mo3C2)
Mo MXenes	Molybdenum carbide (Mo4C3)
Mo MXenes	Molybdenum carbide fluoride (Mo2CF2)
Mo MXenes	Molybdenum carbide oxide (Mo2CO2)
Mo MXenes	Molybdenum carbide sulfide (Mo2CS2)
Mo MXenes	Molybdenum nitride (Mo2N)
Mo MXenes	Molybdenum nitride oxide (Mo2NO2)
Mo Ti MXenes	Molybdenum titanium carbide (Mo2Ti2C3)
Mo Ti MXenes	Molybdenum titanium carbide (Mo2TiC2)
Mo Ti MXenes	Molybdenum titanium carbide oxide (Mo2TiC2O2)
Nb MXenes	Niobium carbide (Nb2C)
Nb MXenes	Niobium carbide (Nb3C2)
Nb MXenes	Niobium carbide (Nb4C3)
Nb MXenes	Niobium carbide fluoride (Nb2CF2)
Nb MXenes	Niobium carbide hydroxide (Nb2C(OH)2)
Nb MXenes	Niobium carbide oxide (Nb2CO2)
Nb MXenes	Niobium carbide oxide (Nb3C2O2)
Nb MXenes	Niobium carbide sulfide (Nb2CS2)
Nb MXenes	Niobium nitride (Nb2N)
Sc MXenes	Scandium carbide (Sc2C)
Sc MXenes	Scandium carbide fluoride (Sc2CF2)
Sc MXenes	Scandium carbide hydroxide (Sc2C(OH)2)
Sc MXenes	Scandium carbide oxide (Sc2CO2)
Sc MXenes	Scandium nitride (Sc2N)
Ta MXenes	Tantalum carbide (Ta2C)
Ta MXenes	Tantalum carbide (Ta3C2)
Ta MXenes	Tantalum carbide (Ta4C3)
Ta MXenes	Tantalum carbide oxide (Ta2CO2)
Ti MXenes	Titanium bromide carbide (Ti3Br2C2)
Ti MXenes	Titanium carbide (Ti2C)
Ti MXenes	Titanium carbide (Ti3C2)
Ti MXenes	Titanium carbide chloride (Ti2CCl2)
Ti MXenes	Titanium carbide chloride (Ti3C2Cl2)
Ti MXenes	Titanium carbide fluoride (Ti2CF2)
Ti MXenes	Titanium carbide fluoride (Ti3C2F2)
Ti MXenes	Titanium carbide fluoride (Ti3C2F)
Ti MXenes	Titanium carbide hydroxide (Ti2C(OH)2)
Ti MXenes	Titanium carbide hydroxide (Ti3C2(OH)2)
Ti MXenes	Titanium carbide hydroxide (Ti3C2(OH))

Ti MXenes	Titanium carbide iodide (Ti3C2I2)
Ti MXenes	Titanium carbide nitride (Ti3CN)
Ti MXenes	Titanium carbide oxide (Ti2CO2)
Ti MXenes	Titanium carbide oxide (Ti3C2O2)
Ti MXenes	Titanium carbide oxide (Ti3C2O)
Ti MXenes	Titanium carbide oxide (Ti4C3O2)
Ti MXenes	Titanium carbide sulfide (Ti3C2S2)
Ti MXenes	Titanium nitride (Ti2N)
Ti MXenes	Titanium nitride (Ti3N2)
Ti MXenes	Titanium nitride oxide (Ti2NO2)
V MXenes	Vanadium carbide (V2C)
V MXenes	Vanadium carbide (V3C2)
V MXenes	Vanadium carbide (V4C3)
V MXenes	Vanadium carbide fluoride (V2CF2)
V MXenes	Vanadium carbide hydroxide (V2C(OH)2)
V MXenes	Vanadium carbide oxide (V2CO2)
V MXenes	Vanadium carbide oxide (V3C2O2)
V MXenes	Vanadium nitride (V2N)
V MXenes	Vanadium nitride oxide (V2NO2)
W MXenes	Tungsten carbide (W2C)
W MXenes	Tungsten carbide oxide (W2CO2)
Y MXenes	Yttrium carbide fluoride (Y2CF2)
Y MXenes	Yttrium carbide oxide (Y2CO2)
Zr MXenes	Zirconium carbide (Zr2C)
Zr MXenes	Zirconium carbide (Zr3C2)
Zr MXenes	Zirconium carbide fluoride (Zr2CF2)
Zr MXenes	Zirconium carbide hydroxide (Zr2C(OH)2)
Zr MXenes	Zirconium carbide oxide (Zr2CO2)
Zr MXenes	Zirconium carbide oxide (Zr3C2O2)
Zr MXenes	Zirconium nitride (Zr2N)

Table S2C: MXenes grouped by anion

**MXene anion group**	**Substance Name**
Carbide	Chromium carbide (Cr2C)
Carbide	Chromium carbide fluoride (Cr2CF2)
Carbide	Chromium carbide oxide (Cr2CO2)
Carbide	Chromium titanium carbide (Cr2TiC2)
Carbide	Chromium vanadium carbide (Cr1.5V1.5C2)
Carbide	Hafnium carbide (Hf2C)
Carbide	Hafnium carbide (Hf3C2)
Carbide	Hafnium carbide oxide (Hf2CO2)
Carbide	Hafnium carbide oxide (Hf3C2O2)
Carbide	Molybdenum carbide (Mo2C)
Carbide	Molybdenum carbide (Mo3C2)

Carbide	Molybdenum carbide (Mo4C3)
Carbide	Molybdenum carbide fluoride (Mo2CF2)
Carbide	Molybdenum carbide oxide (Mo2CO2)
Carbide	Molybdenum carbide sulfide (Mo2CS2)
Carbide	Molybdenum scandium carbide (Mo2ScC2)
Carbide	Molybdenum titanium carbide (Mo2Ti2C3)
Carbide	Molybdenum titanium carbide (Mo2TiC2)
Carbide	Molybdenum titanium carbide oxide (Mo2TiC2O2)
Carbide	Molybdenum vanadium carbide (Mo2VC2)
Carbide	Molybdenum vanadium carbide (Mo4VC4)
Carbide	Niobium carbide (Nb2C)
Carbide	Niobium carbide (Nb3C2)
Carbide	Niobium carbide (Nb4C3)
Carbide	Niobium carbide fluoride (Nb2CF2)
Carbide	Niobium carbide hydroxide (Nb2C(OH)2)
Carbide	Niobium carbide oxide (Nb2CO2)
Carbide	Niobium carbide oxide (Nb3C2O2)
Carbide	Niobium carbide sulfide (Nb2CS2)
Carbide	Scandium carbide (Sc2C)
Carbide	Scandium carbide fluoride (Sc2CF2)
Carbide	Scandium carbide hydroxide (Sc2C(OH)2)
Carbide	Scandium carbide oxide (Sc2CO2)
Carbide	Tantalum carbide (Ta2C)
Carbide	Tantalum carbide (Ta3C2)
Carbide	Tantalum carbide (Ta4C3)
Carbide	Tantalum carbide oxide (Ta2CO2)
Carbide	Titanium bromide carbide (Ti3Br2C2)
Carbide	Titanium carbide (Ti2C)
Carbide	Titanium carbide (Ti3C2)
Carbide	Titanium carbide chloride (Ti2CCl2)
Carbide	Titanium carbide chloride (Ti3C2Cl2)
Carbide	Titanium carbide fluoride (Ti2CF2)
Carbide	Titanium carbide fluoride (Ti3C2F2)
Carbide	Titanium carbide fluoride (Ti3C2F)
Carbide	Titanium carbide hydroxide (Ti2C(OH)2)
Carbide	Titanium carbide hydroxide (Ti3C2(OH)2)
Carbide	Titanium carbide hydroxide (Ti3C2(OH))
Carbide	Titanium carbide iodide (Ti3C2I2)
Carbide	Titanium carbide oxide (Ti2CO2)
Carbide	Titanium carbide oxide (Ti3C2O2)
Carbide	Titanium carbide oxide (Ti3C2O)
Carbide	Titanium carbide oxide (Ti4C3O2)

Carbide	Titanium carbide sulfide (Ti2CS2)
Carbide	Titanium carbide sulfide (Ti3C2S2)
Carbide	Tungsten carbide (W2C)
Carbide	Tungsten carbide oxide (W2CO2)
Carbide	Vanadium carbide (V2C)
Carbide	Vanadium carbide (V3C2)
Carbide	Vanadium carbide (V4C3)
Carbide	Vanadium carbide fluoride (V2CF2)
Carbide	Vanadium carbide hydroxide (V2C(OH)2)
Carbide	Vanadium carbide oxide (V2CO2)
Carbide	Vanadium carbide oxide (V3C2O2)
Carbide	Yttrium carbide fluoride (Y2CF2)
Carbide	Yttrium carbide oxide (Y2CO2)
Carbide	Zirconium carbide (Zr2C)
Carbide	Zirconium carbide (Zr3C2)
Carbide	Zirconium carbide fluoride (Zr2CF2)
Carbide	Zirconium carbide hydroxide (Zr2C(OH)2)
Carbide	Zirconium carbide oxide (Zr2CO2)
Carbide	Zirconium carbide oxide (Zr3C2O2)
Carbonitride	Titanium carbide nitride (Ti3CN)
Nitride	Chromium nitride (Cr2N)
Nitride	Chromium nitride oxide (Cr2NO2)
Nitride	Hafnium nitride (Hf2N)
Nitride	Molybdenum nitride (Mo2N)
Nitride	Molybdenum nitride oxide (Mo2NO2)
Nitride	Niobium nitride (Nb2N)
Nitride	Scandium nitride (Sc2N)
Nitride	Titanium nitride (Ti2N)
Nitride	Titanium nitride (Ti3N2)
Nitride	Titanium nitride oxide (Ti2NO2)
Nitride	Vanadium nitride (V2N)
Nitride	Vanadium nitride oxide (V2NO2)
Nitride	Zirconium nitride (Zr2N)
**Table S2D: MXenes grouped by surface termination**	
**MXenes surface termination group**	**Substance Name**
Chlorine terminated	Titanium carbide chloride (Ti2CCl2)
Chlorine terminated	Titanium carbide chloride (Ti3C2Cl2)
Fluorine terminated	Chromium carbide fluoride (Cr2CF2)
Fluorine terminated	Molybdenum carbide fluoride (Mo2CF2)
Fluorine terminated	Niobium carbide fluoride (Nb2CF2)
Fluorine terminated	Scandium carbide fluoride (Sc2CF2)
Fluorine terminated	Titanium carbide fluoride (Ti2CF2)
Fluorine terminated	Titanium carbide fluoride (Ti3C2F2)

Fluorine terminated	Titanium carbide fluoride (Ti3C2F)
Fluorine terminated	Vanadium carbide fluoride (V2CF2)
Fluorine terminated	Yttrium carbide fluoride (Y2CF2)
Fluorine terminated	Zirconium carbide fluoride (Zr2CF2)
Hydroxyl terminated	Niobium carbide hydroxide (Nb2C(OH)2)
Hydroxyl terminated	Scandium carbide hydroxide (Sc2C(OH)2)
Hydroxyl terminated	Titanium carbide hydroxide (Ti2C(OH)2)
Hydroxyl terminated	Titanium carbide hydroxide (Ti3C2(OH)2)
Hydroxyl terminated	Titanium carbide hydroxide (Ti3C2(OH))
Hydroxyl terminated	Vanadium carbide hydroxide (V2C(OH)2)
Hydroxyl terminated	Zirconium carbide hydroxide (Zr2C(OH)2)
Oxygen terminated	Chromium carbide oxide (Cr2CO2)
Oxygen terminated	Chromium nitride oxide (Cr2NO2)
Oxygen terminated	Hafnium carbide oxide (Hf2CO2)
Oxygen terminated	Hafnium carbide oxide (Hf3C2O2)
Oxygen terminated	Molybdenum carbide oxide (Mo2CO2)
Oxygen terminated	Molybdenum nitride oxide (Mo2NO2)
Oxygen terminated	Molybdenum titanium carbide oxide (Mo2TiC2O2)
Oxygen terminated	Niobium carbide oxide (Nb2CO2)
Oxygen terminated	Niobium carbide oxide (Nb3C2O2)
Oxygen terminated	Scandium carbide oxide (Sc2CO2)
Oxygen terminated	Tantalum carbide oxide (Ta2CO2)
Oxygen terminated	Titanium carbide oxide (Ti2CO2)
Oxygen terminated	Titanium carbide oxide (Ti3C2O2)
Oxygen terminated	Titanium carbide oxide (Ti3C2O)
Oxygen terminated	Titanium carbide oxide (Ti4C3O2)
Oxygen terminated	Titanium nitride oxide (Ti2NO2)
Oxygen terminated	Tungsten carbide oxide (W2CO2)
Oxygen terminated	Vanadium carbide oxide (V2CO2)
Oxygen terminated	Vanadium carbide oxide (V3C2O2)
Oxygen terminated	Vanadium nitride oxide (V2NO2)
Oxygen terminated	Yttrium carbide oxide (Y2CO2)
Oxygen terminated	Zirconium carbide oxide (Zr2CO2)
Oxygen terminated	Zirconium carbide oxide (Zr3C2O2)
Sulfur terminated	Molybdenum carbide sulfide (Mo2CS2)
Sulfur terminated	Niobium carbide sulfide (Nb2CS2)
Sulfur terminated	Titanium carbide sulfide (Ti2CS2)
Sulfur terminated	Titanium carbide sulfide (Ti3C2S2)

Table S3 List of 2D metal chalcogenides grouped by various parameters
Table S3.1 List of TMDs grouped by various parameters

Table S3.1A TMDs grouped by structure and composition	
Conventional	Hafnium selenide (HfSe2)
Conventional	Hafnium sulfide (HfS2)
Conventional	Hafnium telluride (HfTe2)
Conventional	Molybdenum disulfide (MOS 2)
Conventional	Molybdenum selenide (MoSe2)
Conventional	Molybdenum telluride (MoTe2)
Conventional	Niobium selenide (NbSe2)
Conventional	Niobium sulfide (NbS2)
Conventional	Niobium telluride (NbTe2)
Conventional	Rhenium selenide (ReSe2)
Conventional	Rhenium sulfide (ReS2)
Conventional	Rhenium telluride (ReTe2)
Conventional	Tantalum selenide (TaSe2)
Conventional	Tantalum sulfide (TaS2)
Conventional	Tantalum telluride (TaTe2)
Conventional	Titanium disulfide
Conventional	Titanium selenide (TiSe2)
Conventional	Titanium telluride (TiTe2)
Conventional	Tungsten disulfide
Conventional	Tungsten selenide (WSe2)
Conventional	Tungsten telluride (WTe2)
Conventional	Vanadium selenide (VSe2)
Conventional	Vanadium sulfide (VS2)
Conventional	Vanadium telluride (VTe2)
Conventional	Zirconium selenide
Conventional	Zirconium sulfide (ZrS2)
Conventional	Zirconium telluride (ZrTe2)
Non-conventional	Iridium selenide (IrSe2)
Non-conventional	Iridium sulfide (IrS2)
Non-conventional	Iridium telluride (IrTe2)
Non-conventional	Osmium selenide (OsSe2)
Non-conventional	Osmium sulfide (OsS2)
Non-conventional	Osmium telluride (OsTe2)
Non-conventional	Palladium selenide (PdSe2)
Non-conventional	Palladium sulfide (PdS2)
Non-conventional	Palladium telluride (PdTe2)
Non-conventional	Platinum selenide (PtSe2)
Non-conventional	Platinum sulfide (PtS2)
Non-conventional	Platinum telluride
Non-conventional	Ruthenium disulfide
Non-conventional	Ruthenium selenide (RuSe2)

Non-conventional	Ruthenium telluride (RuTe) (RuTe2)
Others	Cobalt selenide (CoSe2)
Others	Cobalt sulfide (CoS2)
Others	Cobalt telluride (CoTe2)
Others	Iron selenide (FeSe2)
Others	Iron telluride (FeTe2)
Others	Manganese selenide (MnSe2)
Others	Manganese telluride (MnTe2)
Others	Nickel selenide (NiSe2)
Others	Nickel telluride (NiTe2)
Others	Tellurium selenide (TeSe2)
Others	Tellurium sulfide (TeS2)
Others	Yttrium sulfide (YS2)
Janus	Chromium selenide sulfide
Janus	Chromium selenide sulfide (CrSeS)
Janus	Chromium selenide telluride
Janus	Chromium selenide telluride (CrSeTe)
Janus	Manganese selenide sulfide (Mn(Se,S))
Janus	Molybdenum selenide sulfide (MoSeS)
Janus	Molybdenum selenide telluride
Janus	Molybdenum sulfide telluride
Janus	Tungsten selenide sulfide (WSeS)
Janus	Tungsten selenide telluride (WSeTe)
Janus	Vanadium selenide sulfide (VSeS)
Tunable	Chromium selenide (CrSe2)
Tunable	Chromium sulfide (CrS2)
Tunable	Chromium telluride (CrTe2)
Tunable	Technetium selenide (99TcSe2)
Tunable	Technetium sulfide (99TcS2)
Tunable	Technetium telluride (99TcTe2)
TMD alloys	Molybdenum selenide sulfide (Mo(Se,S)2)
TMD alloys	Molybdenum sulfide telluride (Mo(S,Te)2)
TMD alloys	Molybdenum tungsten selenide ((Mo,W)Se2)
TMD alloys	Molybdenum tungsten sulfide ((Mo,W)S2)
TMD alloys	Molybdenum tungsten telluride ((Mo,W)Te2)
TMD alloys	Tungsten selenide sulfide (W(Se,S)2)
TMD alloys	Vanadium selenide telluride (VSeTe)
Table S3.1B: TMDs grouped by anions	
Sulfides	Molybdenum disulfide MOS 2
Sulfides	Tungsten disulfide
Sulfides	Rhenium sulfide (ReS2)
Sulfides	Tantalum sulfide (TaS2)

Sulfides	Titanium disulfide
Sulfides	Hafnium sulfide (HfS2)
Sulfides	Niobium sulfide (NbS2)
Sulfides	Vanadium sulfide (VS2)
Sulfides	Zirconium sulfide (ZrS2)
Sulfides	Platinum sulfide (PtS2)
Sulfides	Palladium sulfide (PdS2)
Sulfides	Chromium sulfide (CrS2)
Sulfides	Molybdenum tungsten sulfide ((Mo,W)S2)
Sulfides	Molybdenum sulfide
Sulfides	Technetium sulfide (99TcS2)
Sulfides	Ruthenium disulfide
Sulfides	Osmium sulfide (OsS2)
Sulfides	Rhodium sulfide (RhS2)
Sulfides	Iridium sulfide (IrS2)
Sulfides	Titanium sulfide
Sulfides	Rhodium sulfide (RhS3)
Selenides	Tungsten selenide (WSe2) & WSE 2
Selenides	Molybdenum selenide (MoSe2)
Selenides	Niobium selenide (NbSe2)
Selenides	Platinum selenide (PtSe2)
Selenides	Vanadium selenide (VSe2)
Selenides	Titanium selenide (TiSe2)
Selenides	Rhenium selenide (ReSe2)
Selenides	Palladium selenide (PdSe2)
Selenides	Tantalum selenide (TaSe2)
Selenides	Hafnium selenide (HfSe2)
Selenides	Tungsten selenide
Selenides	Chromium selenide (CrSe2)
Selenides	Molybdenum selenide
Selenides	Molybdenum tungsten selenide ((Mo,W)Se2)
Selenides	Technetium selenide (99TcSe2)
Selenides	Zirconium selenide
Selenides	Ruthenium selenide (RuSe2)
Selenides	Iridium selenide (IrSe2)
Selenides	Osmium selenide (OsSe2)
Selenides	Rhodium selenide (RhSe2)
Selenides	Platinum selenide (PtSe)
Selenides	Ruthenium selenide (RuSe)
Selenides	Palladium selenide (PdSe)
Tellurides	Molybdenum telluride (MoTe2)
Tellurides	Tungsten telluride (WTe2)

Tellurides	Niobium telluride (NbTe2)
Tellurides	Tantalum telluride (TaTe2)
Tellurides	Palladium telluride (PdTe2)
Tellurides	Hafnium telluride (HfTe2)
Tellurides	Chromium telluride (CrTe2)
Tellurides	Titanium telluride (TiTe2)
Tellurides	Zirconium telluride (ZrTe2)
Tellurides	Vanadium telluride (VTe2)
Tellurides	Rhenium telluride (ReTe2)
Tellurides	Chromium telluride
Tellurides	Iridium telluride (IrTe2)
Tellurides	Ruthenium telluride (RuTe) & Ruthenium telluride (RuTe2)
Tellurides	Platinum telluride
Tellurides	Technetium telluride (99TcTe2)
Tellurides	Molybdenum tungsten telluride ((Mo,W)Te2)
Tellurides	Osmium telluride (OsTe2)
Selenide Sulfide	Molybdenum selenide sulfide (MoSeS)
Selenide Sulfide	Tungsten selenide sulfide (WSeS)
Selenide Sulfide	Vanadium selenide sulfide (VSeS)
Selenide Sulfide	Chromium selenide sulfide (CrSeS)
Selenide Sulfide	Tungsten selenide sulfide (W(Se,S)2)
Selenide Sulfide	Manganese selenide sulfide (Mn(Se,S))
Selenide Sulfide	Chromium selenide sulfide
Selenide Telluride	Tungsten selenide telluride (WSeTe)
Selenide Telluride	Vanadium selenide telluride (VSeTe)
Selenide Telluride	Chromium selenide telluride (CrSeTe)
Selenide Telluride	Chromium selenide telluride
Selenide Telluride	Molybdenum selenide telluride
Sulfide Telluride	Molybdenum sulfide telluride (Mo(S,Te)2)
Sulfide Telluride	Molybdenum sulfide telluride
Table S3.1C TMDs grouped by metal	
Molybdenum	Molybdenum disulfide
Molybdenum	Molybdenum selenide (MoSe2)
Molybdenum	Molybdenum telluride (MoTe2)
Molybdenum	Molybdenum selenide sulfide (MoSeS)
Molybdenum	Molybdenum sulfide
Molybdenum	Molybdenum selenide
Molybdenum	Molybdenum sulfide telluride
Molybdenum	Molybdenum selenide telluride
Tungsten	Tungsten disulfide
Tungsten	Tungsten selenide (WSe2)
Tungsten	Tungsten telluride (WTe2)

Tungsten	Tungsten selenide
Tungsten	Tungsten selenide sulfide (WSeS)
Tungsten	Tungsten selenide telluride (WSeTe)
Niobdium	Niobium selenide (NbSe2)
Niobdium	Niobium sulfide (NbS2)
Niobdium	Niobium telluride (NbTe2)
Rhenium	Rhenium sulfide (ReS2)
Rhenium	Rhenium selenide (ReSe2)
Rhenium	Rhenium telluride (ReTe2)
Tantalum	Tantalum sulfide (TaS2)
Tantalum	Tantalum selenide (TaSe2)
Tantalum	Tantalum telluride (TaTe2)
Vanadium	Vanadium selenide (VSe2)
Vanadium	Vanadium sulfide (VS2)
Vanadium	Vanadium telluride (VTe2)
Vanadium	Vanadium selenide sulfide (VSeS)
Titanium	Titanium disulfide
Titanium	Titanium selenide (TiSe2)
Titanium	Titanium telluride (TiTe2)
Titanium	Titanium sulfide
Platinum	Platinum selenide (PtSe2)
Platinum	Platinum sulfide (PtS2)
Platinum	Platinum telluride
Platinum	Platinum selenide (PtSe)
Hafnium	Hafnium sulfide (HfS2)
Hafnium	Hafnium selenide (HfSe2)
Hafnium	Hafnium telluride (HfTe2)
Palladium	Palladium selenide (PdSe2)
Palladium	Palladium telluride (PdTe2)
Palladium	Palladium sulfide (PdS2)
Palladium	Palladium selenide (PdSe)
Zirconium	Zirconium sulfide (ZrS2)
Zirconium	Zirconium telluride (ZrTe2)
Zirconium	Zirconium selenide
Chromium	Chromium telluride (CrTe2)
Chromium	Chromium selenide (CrSe2)
Chromium	Chromium sulfide (CrS2)
Chromium	Chromium telluride
Chromium	Chromium selenide sulfide (CrSeS)
Chromium	Chromium selenide telluride (CrSeTe)
Chromium	Chromium selenide telluride
Chromium	Chromium selenide sulfide

Molybdenum Tungsten	Molybdenum tungsten sulfide ((Mo,W)S2)
Molybdenum Tungsten	Molybdenum tungsten selenide ((Mo,W)Se2)
Molybdenum Tungsten	Molybdenum selenide sulfide (Mo(Se,S)2)
Molybdenum Tungsten	Molybdenum tungsten telluride ((Mo,W)Te2)
Other	Vanadium selenide telluride (VSeTe)
Other	Tungsten selenide sulfide (W(Se,S)2)
Other	Molybdenum sulfide telluride (Mo(S,Te)2)
Other	Tungsten selenide sulfide (W(Se,S))
Ruthenium	Ruthenium telluride (RuTe) & Ruthenium telluride (RuTe2)
Ruthenium	Ruthenium disulfide
Ruthenium	Ruthenium selenide (RuSe2)
Ruthenium	Ruthenium selenide (RuSe)
Iridium	Iridium telluride (IrTe2)
Iridium	Iridium selenide (IrSe2)
Iridium	Iridium sulfide (IrS2)
Technetium	Technetium sulfide (99TcS2)
Technetium	Technetium selenide (99TcSe2)
Technetium	Technetium telluride (99TcTe2)
Osmium	Osmium sulfide (OsS2)
Osmium	Osmium telluride (OsTe2)
Osmium	Osmium selenide (OsSe2)
Rhodium	Rhodium sulfide (RhS2)
Rhodium	Rhodium selenide (RhSe2)
Rhodium	Rhodium sulfide (RhS3)
Manganese	Manganese selenide sulfide (Mn(Se,S))

Table S3.2: List of Other 2D metal chalcogenides

Table S3.2A: Grouped by Anions

Selenides	Indium selenide
Selenides	Bismuth selenide (Bi2Se3)
Selenides	Tin selenide
Selenides	Gallium selenide (GaSe)
Selenides	Germanium diselenide,
Selenides	Antimony selenide (Sb2Se3)
Sulfides	Tin sulfide (SnS)
Sulfides	Germanium sulfide (GeS)
Sulfides	Gallium sulfide (GaS)
Sulfides	Indium sulfide (In2S3), Indium sulfide (InS)
Sulfides	Bismuth sulfide (Bi2S3)
Sulfides	Antimony sulfide (Sb2S3)
Tellurides	Bismuth telluride, Bismuth telluride (Bi2Te), Bismuth telluride (Bi4Te5)
Tellurides	Gallium telluride
Tellurides	Antimony telluride (Sb2Te3)

Tellurides	Tin telluride (SnTe)
Tellurides	Indium telluride (InTe)
Mixed	Germanium iron telluride (GeFe3Te2), Germanium iron telluride (GeFe4Te2), Germanium iron telluride (GeFe5Te2)
Mixed	Bismuth manganese telluride (Bi2MnTe4)
Mixed	Gallium indium sulfide (GaInS2) & Gallium indium sulfide (GaInS3)
Mixed	Indium selenide sulfide & Indium selenide sulfide (InSe0.5S0.5)
Mixed	Antimony selenide telluride (Sb2Se2Te) & Antimony selenide telluride (Sb2SeTe2)
Mixed	Bismuth selenide telluride, Bismuth selenide telluride (Bi2Se0.3Te2.7), Bismuth selenide telluride (Bi4Se3Te3)
Mixed	Antimony germanium telluride
Mixed	Germanium iron telluride (GeFe0-5Te2), Germanium iron telluride (GeFe2.84Te2.1), Germanium iron telluride (GeFe2.8Te2) and 4 more
Mixed	Gallium indium selenide, Gallium indium selenide ((Ga,In)Se), Gallium indium selenide (GaInSe1.88) and 1 more
Mixed	Bismuth sulfide telluride (Bi2STe2)
Mixed	Bismuth sulfide telluride (Bi2S2Te) & Bismuth sulfide telluride (Bi4S1.5Te1.5)
Mixed	Bismuth selenide sulfide, Bismuth selenide sulfide (Bi2Se2S), Bismuth selenide sulfide (Bi2SeS2)
Mixed	Germanium iron telluride
Mixed	Indium selenide telluride (In2SeTe2) & Indium selenide telluride (InSe0.9Te0.1)
Mixed	Indium selenide sulfide (In2Se0.94S0.94), Indium selenide sulfide (In2Se2S), Indium selenide sulfide (InSe0.8S0.2) and 1 more
Mixed	Germanium selenide telluride (Ge(Se,Te)) & Germanium selenide telluride (GeSe0.75Te0.25)
Mixed	Germanium selenide sulfide (Ge(Se,S)), Germanium selenide sulfide (GeSe0.25S0.75), Germanium selenide sulfide (GeSe0.27S0.73) and 5 more
Mixed	Germanium iron nickel telluride (Ge(Fe,Ni)5Te2), Germanium iron nickel telluride (GeFe2NiTe2), Germanium iron nickel telluride (GeFe3.5Ni1.5Te2) and 3 more
Mixed	Gallium indium selenide telluride, Gallium indium selenide telluride (GaInSe2Te), Gallium indium selenide telluride (GaInSeTe2) and 4 more
Mixed	Antimony bismuth telluride (SbBiTe3)
Mixed	Indium tin sulfide (InSnS2) & Indium tin sulfide (InSnS3)
Mixed	Germanium iron nickel telluride (GeFe4.25Ni0.75Te2)
Mixed	Gallium indium selenide sulfide, Gallium indium selenide sulfide (GaInSe2S), Gallium indium selenide sulfide (GaInSeS2)
Mixed	Indium tin selenide (In0.95Sn0.05Se), Indium tin selenide (In0.99Sn0.01Se), Indium tin selenide (In0.9Sn0.1Se)
Table S3.2B: Other 2D chalcogenides grouped by metal	
Indium	Indium selenide (InSe)
Indium	Indium selenide (In2Se3)
Indium	Indium sulfide (In2S3)

Indium	Indium sulfide (InS)
Indium	Indium telluride (InTe)
Indium	Indium selenide
Indium	Indium selenide (In4Se3)
Indium	Indium selenide sulfide (InSe0.5S0.5)
Indium	Indium selenide telluride (In2SeTe2)
Indium	Indium selenide sulfide
Indium	Indium tin sulfide (InSnS3)
Indium	Indium tin sulfide (InSnS2)
Indium	Indium tin selenide (In0.9Sn0.1Se)
Indium	Indium tin selenide (In0.99Sn0.01Se)
Indium	Indium tin selenide (In0.95Sn0.05Se)
Indium	Indium selenide telluride (InSe0.9Te0.1)
Indium	Indium selenide sulfide (InSe0.9S0.1)
Indium	Indium selenide sulfide (InSe0.8S0.2)
Indium	Indium selenide sulfide (In2Se2S)
Indium	Indium selenide sulfide (In2Se0.94S0.94)
Bismuth	Bismuth selenide (Bi2Se3)
Bismuth	Bismuth telluride
Bismuth	Bismuth sulfide (Bi2S3)
Bismuth	Bismuth manganese telluride (Bi2MnTe4)
Bismuth	Bismuth selenide (BiSe)
Bismuth	Bismuth telluride (BiTe)
Bismuth	Bismuth sulfide telluride (Bi2STe2)
Bismuth	Bismuth sulfide telluride (Bi2S2Te)
Bismuth	Bismuth telluride (Bi4Te5)
Bismuth	Bismuth telluride (Bi2Te)
Bismuth	Bismuth sulfide telluride (Bi4S1.5Te1.5)
Bismuth	Bismuth selenide telluride (Bi4Se3Te3)
Bismuth	Bismuth selenide telluride (Bi2Se0.3Te2.7)
Bismuth	Bismuth selenide telluride
Bismuth	Bismuth selenide sulfide (Bi2SeS2)
Bismuth	Bismuth selenide sulfide (Bi2Se2S)
Bismuth	Bismuth selenide sulfide
Bismuth	Bismuth tin telluride (Bi6SnTe10)
Bismuth	Bismuth tin telluride (Bi2SnTe4)
Bismuth	Bismuth tin selenide telluride (Bi2SnSeTe3)
Germanium	Germanium selenide (GeSe)
Germanium	Germanium iron telluride (GeFe3Te2)
Germanium	Germanium sulfide (GeS)
Germanium	Germanium telluride (GeTe)

Germanium	Germanium selenide
Germanium	Germanium sulfide
Germanium	Germanium iron telluride (GeFe5Te2)
Germanium	Germanium iron telluride (GeFe4Te2)
Germanium	Germanium diselenide
Germanium	Germanium iron telluride
Germanium	Germanium selenide telluride (Ge(Se,Te))
Germanium	Germanium iron nickel telluride (GeFe4.25Ni0.75Te2)
Germanium	Germanium selenide telluride (GeSe0.75Te0.25)
Germanium	Germanium selenide sulfide (GeSeS)
Germanium	Germanium selenide sulfide (GeSe0.75S0.25)
Germanium	Germanium selenide sulfide (GeSe0.71S0.29)
Germanium	Germanium selenide sulfide (GeSe0.58S0.42)
Germanium	Germanium selenide sulfide (GeSe0.54S0.46)
Germanium	Germanium selenide sulfide (GeSe0.27S0.73)
Germanium	Germanium selenide sulfide (GeSe0.25S0.75)
Germanium	Germanium selenide sulfide (Ge(Se,S))
Germanium	Germanium iron telluride (GeFe4.8Te2)
Germanium	Germanium iron telluride (GeFe4.87Te2)
Germanium	Germanium iron telluride (GeFe2.9Te2)
Germanium	Germanium iron telluride (GeFe2.94Te2.12)
Germanium	Germanium iron telluride (GeFe2.8Te2)
Germanium	Germanium iron telluride (GeFe2.84Te2.1)
Germanium	Germanium iron telluride (GeFe0-5Te2)
Germanium	Germanium iron nickel telluride (GeFe4.75Ni0.25Te2)
Germanium	Germanium iron nickel telluride (GeFe4.5Ni0.5Te2)
Germanium	Germanium iron nickel telluride (GeFe4.01Ni0.68Te2)
Germanium	Germanium iron nickel telluride (GeFe3.5Ni1.5Te2)
Germanium	Germanium iron nickel telluride (GeFe2NiTe2)
Germanium	Germanium iron nickel telluride (Ge(Fe,Ni)5Te2)
Tin	Tin sulfide (SnS)
Tin	Tin selenide (SnSe)
Tin	Tin sulfide
Tin	Tin selenide
Tin	Tin telluride (SnTe)
Gallium	Gallium selenide (GaSe)
Gallium	Gallium sulfide (GaS)
Gallium	Gallium telluride (GaTe)
Gallium	Gallium sulfide
Gallium	Gallium telluride
Gallium	Gallium selenide

Gallium	Gallium indium sulfide (GaInS3)
Gallium	Gallium indium sulfide (GaInS2)
Gallium	Gallium indium selenide
Gallium	Gallium indium sulfide telluride (GaInSTe2)
Gallium	Gallium indium sulfide telluride (GaInS2Te2)
Gallium	Gallium indium sulfide telluride (GaInS2Te)
Gallium	Gallium indium sulfide telluride
Gallium	Gallium indium selenide telluride (GaInSeTe2)
Gallium	Gallium indium selenide telluride (GaInSe2Te)
Gallium	Gallium indium selenide telluride
Gallium	Gallium indium selenide sulfide (GaInSeS2)
Gallium	Gallium indium selenide sulfide (GaInSe2S)
Gallium	Gallium indium selenide sulfide
Gallium	Gallium indium selenide (GaInSe3)
Gallium	Gallium indium selenide (GaInSe1.88)
Gallium	Gallium indium selenide ((Ga,In)Se)
Antimony	Antimony telluride (Sb2Te3)
Antimony	Antimony sulfide (Sb2S3)
Antimony	Antimony selenide (Sb2Se3)
Antimony	Antimony selenide telluride (Sb2SeTe2)
Antimony	Antimony germanium telluride
Antimony	Antimony selenide telluride (Sb2Se2Te)
Antimony	Antimony bismuth telluride (SbBiTe3)
Antimony	Antimony selenide telluride (Sb2(Se,Te)3)
Antimony	Antimony selenide telluride
Antimony	Antimony selenide sulfide (Sb2SeS2)
Antimony	Antimony selenide sulfide (Sb2(Se,S)3)
Antimony	Antimony germanium telluride (SbGeTe3)
Antimony	Antimony germanium telluride (Sb3Ge2Te5)
Antimony	Antimony germanium telluride (Sb2GeTe4)
Antimony	Antimony bismuth telluride (Sb1.74Bi0.26Te3)
Antimony	Antimony bismuth telluride (Sb1.6Bi0.4Te3)
Antimony	Antimony bismuth telluride (Sb0.8Bi1.2Te3)
Antimony	Antimony bismuth telluride (Sb0.6Bi1.4Te3)
Antimony	Antimony bismuth telluride (Sb0.4Bi1.6Te3)
Antimony	Antimony bismuth telluride (Sb0.2Bi1.8Te3)
Antimony	Antimony bismuth telluride ((Sb,Bi)2Te3)
Antimony	Antimony bismuth telluride

**Table S4: List of substances grouped in metal-free nitride**	
**Meta-free nitride group**	**Substance name**
Boron nitride	Boron nitride
Boron nitride	Boronitrene
Carbon nitride	Carbon nitride
Carbon nitride	Carbon nitride (C3N4)

TableS5 List of Metal halides grouped by various parameters	
Table S5A Metal halides grouped by anions	
Iodide	Lead iodide (PbI2)
Iodide	Chromium iodide (CrI3) & (CrI)
Iodide	Nickel iodide (NiI2)
Iodide	Cadmium iodide
Iodide	Cobalt iodide (CoI2)
Iodide	Iron iodide (FeI2)
Iodide	Manganese iodide (MnI2)
Bromide	Lead dibromide
Bromide	Nickel bromide
Bromide	Chromium bromide (CrBr3)
Bromide	Cobalt dibromide
Oxyhalides	Bismuth oxychloride
Oxyhalides	Bismuth oxybromide
Oxyhalides	Bismuth oxyiodide
Chloride	Chromium chloride (CrCl3)
Chloride	Vanadium trichloride
Table S5B Metal halides grouped by metal	
Lead	Lead dibromide
Lead	Lead iodide (PbI2)
Chromium	Chromium iodide (CrI3) & (CrI)
Chromium	Chromium bromide (CrBr3)
Chromium	Chromium chloride (CrCl3)
Nickel	Nickel bromide
Nickel	Nickel iodide (NiI2)
Manganese	Manganese iodide (MnI2)
Vanadium	Vanadium trichloride
Bismuth	Bismuth oxychloride
Bismuth	Bismuth oxybromide
Bismuth	Bismuth oxyiodide
Iron	Iron iodide (FeI2)
Cadmium	Cadmium iodide

Cobalt	Cobalt iodide (CoI2)
Cobalt	Cobalt dibromide

**Table S6: Organic frameworks grouped by different parameters**	
**Table S6A: MOFs grouped by metal types**	
**MOF types**	**MOF substance names**
Cu-based	Copper
Cu-based	Triaqua[μ-[1,3,5-benzenetricarboxylato(3-)-κO1:κO′1]][μ3-[1,3,5-benzenetricarboxylato(3-)-κO1:κO3:κO′1]]tricopper
Cu-based	Bis[μ-[2,3,6,7,10,11-triphenylenehexolato(6-)-κO2,κO3:κO6,κO7]]tricopper
Cu-based	Stereoisomer of bis[μ-[1,2,3,4,5,6-benzenehexaminato(6-)-κN1,κN2:κN3,κN4]]tricopper
Cu-based	(SP-4-1)-[29H,31H-Phthalocyanine-2,3,9,10,16,17,23,24-octolato(2-)-κN29,κN30,κN31,κN32]copper
Ni-based	Nickel
Ni-based	stereoisomer of Bis[μ-[2,3,6,7,10,11-triphenylenehexaminato(4-)-κN2,κN3:κN6,κN7]]trinickel
Ni-based	Stereoisomer of bis[μ-[1,2,3,4,5,6-benzenehexaminato(6-)-κN1,κN2:κN3,κN4]]trinickel
Co-based	Cobalt
Co-based	ZIF 67
Co-based	Cobalt, bis[μ-[2,3,6,7,10,11-triphenylenehexaminato(4-)-κN2,κN3:κN6,κN7]]tri-, stereoisomer
Co-based	Cobalt 5,10,15,20-tetrakis(4-carboxyphenyl)porphyrin
Zn-based	Zinc
Zn-based	Basolite Z 1200
Zn-based	ZnTBAP
Zn-based	Zinc(2+)
Zn-based	1H-Imidazole, 2-methyl-, zinc salt, hydrate (5:2:3)
Zn-based	ZIF 7
Zn-based	ZIF 8
Zn-based	ZIF 11
Fe-based	Iron
**Table S6B: MOFs grouped by linker types**	
**MOF types**	**MOF substance names**
N-donor	2-Methylimidazole
N-donor	Benzimidazole
N-donor	Pyridine
N-donor	Pyrazine
N-donor	4,4′-Bipyridine
N-donor	1H-Pyrrole
N-donor	Dabco

N-donor	Tris(4-pyridyl)-1,3,5-triazine
N-donor	Piperazine
N-donor	Imidazole
N-donor	Hydrazine hydrate (1:1)
N-donor	4-Vinylpyridine
N-donor	4-Aminopyridine
N-donor	3,5-Di-1H-imidazol-1-ylpyridine
N-donor	2-Aminobenzimidazole
N-donor	1H-Benzimidazole, cobalt(2+) salt (2:1)
N-donor	1,1′-(1,4-Butanediyl)bis(imidazole)
N-donor	Vinylimidazole
N-donor	2,3,6,7,10,11-Triphenylenehexamine
N-donor	Melamine
N-donor	Hexaaminobenzene
N-donor	Ethylenediamine
N-donor	Triethanolamine
N-donor	Triethylenetetramine
N-donor	Diethylenetriamine
N-donor	Stereoisomer of bis[μ-[1,2,3,4,5,6-benzenehexaminato(6-)-κN1,κN2:κN3,κN4]]trinickel
N-donor	Stereoisomer of bis[μ-[1,2,3,4,5,6-benzenehexaminato(6-)-κN1,κN2:κN3,κN4]]tricopper
N-donor	stereoisomer of Bis[μ-[2,3,6,7,10,11-triphenylenehexaminato(4-)-κN2,κN3:κN6,κN7]]trinickel
N-donor	Tetrakis(4-carboxyphenyl)porphyrin
N-donor	Tetra(4-pyridyl)porphyrin
N-donor	Porphyrin
N-donor	Iron(II) phthalocyanine
N-donor	Copper phthalocyanine
N-donor	Cobalt 5,10,15,20-tetrakis(4-carboxyphenyl)porphyrin
N-donor	Palladium, [μ3-[[4,4′-[10,20-bis(4-carboxyphenyl)-21H,23H-porphine-5,15-diyl-κN21,κN22,κN23,κN24]bis[benzoato-κO]](6-)]]bis(zinc)-
N-donor	meso-(Tetrakis(4-carboxyphenyl)porphyrinato)copper
N-donor	(SP-4-1)-[29H,31H-Phthalocyanine-2,3,9,10,16,17,23,24-octolato(2-)-κN29,κN30,κN31,κN32]nickel
N-donor	(SP-4-1)-[29H,31H-Phthalocyanine-2,3,9,10,16,17,23,24-octolato(2-)-κN29,κN30,κN31,κN32]iron
N-donor	(SP-4-1)-[29H,31H-Phthalocyanine-2,3,9,10,16,17,23,24-octolato(2-)-κN29,κN30,κN31,κN32]cobalt
N-donor	(SP-4-1)-[2,3,9,10,16,17,23,24-Octamethoxy-29H,31H-phthalocyaninato(2-)-κN29,κN30,κN31,κN32]copper
N-donor	Zinc phthalocyanine
O-donor	Terephthalic acid

O-donor	2-Aminoterephthalic acid
O-donor	1,3,5-Benzenetricarboxylic acid
O-donor	Benzenedicarboxylic acid
O-donor	1,3,5-Tris(4-carboxyphenyl)benzene
O-donor	2,5-Dihydroxyterephthalic acid
O-donor	Isophthalic acid
O-donor	2,5-Thiophenedicarboxylic acid
O-donor	[1,1′-Biphenyl]-4,4′-dicarboxylic acid
O-donor	Succinic acid
O-donor	Oxalic acid
O-donor	Fumaric acid
O-donor	Citric acid
O-donor	Chloranilic acid
O-donor	4,4′,4″,4‴-(1,3,6,8-Pyrenetetrayl)tetrakis[benzoic acid]
O-donor	2,6-Naphthalenedicarboxylic acid
O-donor	2-Pyrazinecarboxylic acid
O-donor	α9,α10-Dicarboxy-9,10-anthracenedipropanoic acid
O-donor	Trimellitic acid
O-donor	Hexaaquabis[μ-[2,3,6,7,10,11-triphenylenehexolato(6-)-κO2,κO3:κO6,κO7]]trinickel
O-donor	Bis[μ-[2,3,6,7,10,11-triphenylenehexolato(6-)-κO2,κO3:κO6,κO7]]trinickel
O-donor	Triquinoyl
O-donor	3,4,9,10-Perylenetetracarboxylic dianhydride
O-donor	2,3,7,8,12,13-Tricycloquinazolinehexol
S-donor	1,2,3,4,5,6-Benzenehexathiol
S-donor	2,3,6,7,10,11-Triphenylenehexathiol
S-donor	[μ3-[1,2,3,4,5,6-Benzenehexathiolato(6-)-κS1,κS2:κS3,κS4:κS5,κS6]]trinickel
S-donor	Bis[μ-[1,2,3,4,5,6-benzenehexathiolato(6-)-κS1,κS2:κS3,κS4]]tricobalt

Table S6c: COFs grouped by linkage types

**COF types**	**COF substance names**
Imine-linked	1,4-Benzenedicarboxaldehyde, 2,5-dimethoxy-, polymer with 5′-(4-aminophenyl)[1,1′:3′,1″-terphenyl]-4,4″-diamine
Imine-linked	1,4-Benzenedicarboxaldehyde, polymer with 5′-(4-aminophenyl)[1,1′:3′,1″-terphenyl]-4,4″-diamine
Imine-linked	1,3,5-Benzenetricarboxaldehyde, polymer with 1,4-benzenediamine
Imine-linked	1,4-Benzenedicarboxaldehyde, polymer with 4,4′,4″,4‴-(1,3,6,8-pyrenetetrayl)tetrakis[benzenamine]
Imine-linked	1,4-Benzenedicarboxaldehyde, polymer with 4,4′,4″,4‴-(21H,23H-porphine-5,10,15,20-tetrayl)tetrakis[benzenamine]
Imine-linked	[1,1′:3′,1″-Terphenyl]-4,4″-dicarboxaldehyde, 5′-(4-formylphenyl)-, polymer with 5′-(4-aminophenyl)[1,1′:3′,1″-terphenyl]-4,4″-diamine
Imine-linked	Benzaldehyde, 4,4′,4″-(1,3,5-triazine-2,4,6-triyl)tris-, polymer with 1,4-benzenediamine
Imine-linked	Benzaldehyde, 4,4′-[3,8-bis(4-aminophenyl)-1,6-pyrenediyl]bis-, homopolymer
Imine-linked	Benzaldehyde, 4,4′,4″-(1,3,5-triazine-2,4,6-triyl)tris-, polymer with 4,4′,4″-(1,3,5-triazine-2,4,6-triyl)tris[benzenamine]

Imine-linked	Benzaldehyde, 4,4′,4″-(1,3,5-triazine-2,4,6-triyl)tris-, polymer with [2,2′-bipyridine]-5,5′-diamine
Imine-linked	1,4-Benzenedicarboxaldehyde, 2,3,5,6-tetrafluoro-, polymer with 5′-(4-aminophenyl)[1,1′:3′,1″-terphenyl]-4,4″-diamine
Imine-linked	1,4-Benzenedicarboxaldehyde, 2,3,5,6-tetrafluoro-, polymer with 4,4′,4″-(1,3,5-triazine-2,4,6-triyl)tris[benzenamine]
Imine-linked	1,3,5-Benzenetricarboxaldehyde, polymer with [1,1′-biphenyl]-4,4′-diamine
Imine-linked	1,2,3,4,5,6-Cyclohexanehexone, polymer with 2,3,6,7,10,11-triphenylenehexamine
Imine-linked	1,2,3,4,5,6-Cyclohexanehexone, polymer with 1,2,4,5-benzenetetramine
Imine-linked	[1,1′-Biphenyl]-4,4′-dicarboxaldehyde, polymer with 4,4′,4″,4‴-(21H,23H-porphine-5,10,15,20-tetrayl)tetrakis[benzenamine]
Imine-linked	Thieno[3,2-b]thiophene-2,5-dicarboxaldehyde, polymer with N1,N1-bis(4-aminophenyl)-1,4-benzenediamine
Imine-linked	Thieno[3,2-b]thiophene-2,5-dicarboxaldehyde, polymer with 5′-(4-aminophenyl)[1,1′:3′,1″-terphenyl]-4,4″-diamine
Imine-linked	Pyrylium, 2,4,6-trimethyl-, tetrafluoroborate(1-) (1:1), polymer with 4′-[4,6-bis(4′-formyl[1,1′-biphenyl]-4-yl)-1,3,5-triazin-2-yl][1,1′-biphenyl]-4-carboxaldehyde
β-Ketoenamine-linked	1,3,5-Benzenetricarboxaldehyde, 2,4,6-trihydroxy-, polymer with 1,4-benzenediamine
β-Ketoenamine-linked	Benzenesulfonic acid, 2,5-diamino-, polymer with 2,4,6-trihydroxy-1,3,5-benzenetricarboxaldehyde
β-Ketoenamine-linked	1,3,5-Benzenetricarboxaldehyde, 2,4,6-trihydroxy-, polymer with [1,1′-biphenyl]-4,4′-diamine
β-Ketoenamine-linked	1,3,5-Benzenetricarboxaldehyde, 2,4,6-trihydroxy-, polymer with [2,2′-bipyridine]-5,5′-diamine
β-Ketoenamine-linked	Phenanthridinium, 3,8-diamino-5-ethyl-6-phenyl-, bromide (1:1), polymer with 2,4,6-trihydroxy-1,3,5-benzenetricarboxaldehyde
β-Ketoenamine-linked	Benzenesulfonic acid, 2,5-diamino-, sodium salt (1:1), polymer with 2,4,6-trihydroxy-1,3,5-benzenetricarboxaldehyde
β-Ketoenamine-linked	Benzaldehyde, 4,4′,4″-(1,3,5-benzenetriyl)tris[2-hydroxy-, polymer with 5′-(4-aminophenyl)[1,1′:3′,1″-terphenyl]-4,4″-diamine
β-Ketoenamine-linked	5′-(4-Aminophenyl)[1,1′:3′,1″-terphenyl]-4,4″-diamine polymer with 2,4,6-trihydroxy-1,3,5-benzenetricarboxaldehyde
β-Ketoenamine-linked	1,4-Benzenedicarboxaldehyde, 2,5-dihydroxy-, polymer with 5′-(4-aminophenyl)[1,1′:3′,1″-terphenyl]-4,4″-diamine
β-Ketoenamine-linked	1,4-Benzenedicarboxaldehyde, 2,5-dihydroxy-, polymer with 4,4′,4″,4‴-(21H,23H-porphine-5,10,15,20-tetrayl)tetrakis[benzenamine]
β-Ketoenamine-linked	1,4-Benzenedicarboxaldehyde, 2,5-dihydroxy-, polymer with 4,4′,4″-(1,3,5-triazine-2,4,6-triyl)tris[benzenamine]
β-Ketoenamine-linked	1,3,5-Benzenetricarboxaldehyde, 2,4,6-trihydroxy-, polymer with 4,4′,4″-(1,3,5-triazine-2,4,6-triyl)tris[benzenamine]
β-Ketoenamine-linked	1,3,5-Benzenetricarboxaldehyde, 2,4,6-trihydroxy-, polymer with 4,4′-(1,2-diazenediyl)bis[benzenamine]
Boronate ester / boroxine-linked	Boronic acid, B,B′-1,4-phenylenebis-, polymer with 2,3,6,7,10,11-triphenylenehexol
Boronate ester / boroxine-linked	Boronic acid, B,B′-1,4-phenylenebis-, homopolymer
Boronate ester / boroxine-linked	Boronic acid, B,B′-2,7-pyrenediylbis-, polymer with 2,3,6,7,10,11-triphenylenehexol
Boronate ester / boroxine-linked	Boronic acid, B,B′-[1,1′-biphenyl]-4,4′-diylbis-, polymer with 2,3,6,7,10,11-triphenylenehexol
Hydrazone-linked	1,3,5-Benzenetricarboxaldehyde, polymer with hydrazine

Hydrazone-linked	1,3,5-Benzenetricarboxaldehyde, 2,4,6-trihydroxy-, polymer with hydrazine

Table S7: List of LDHs	
Ni containing LDHs	Nickel hydroxide oxide (Ni(OH)O)
Ni containing LDHs	Nickel zinc hydroxide
Ni containing LDHs	Nickel vanadium hydroxide (Ni0.75V0.25(OH)2)
Ni containing LDHs	Nickel titanium hydroxide
Co containing LDHs	Cobalt nickel hydroxide
Co containing LDHs	Cobalt iron hydroxide
Co containing LDHs	Cobalt molybdenum hydroxide
Co containing LDHs	Cobalt manganese hydroxide
Co containing LDHs	Cobalt copper hydroxide
Co containing LDHs	Cobalt zinc hydroxide
Co containing LDHs	Cobalt nickel hydroxide ((Co,Ni)(OH)2)
Co containing LDHs	Cobalt molybdenum nickel hydroxide
Co containing LDHs	Cobalt nickel hydroxide (Co2Ni(OH)6)
Co containing LDHs	Cobalt nickel hydroxide (Co2Ni(OH))
Co containing LDHs	Cobalt manganese nickel hydroxide
Co containing LDHs	Cobalt manganese hydroxide oxide ((Co,Mn)(OH)O)
Co containing LDHs	Cobalt lanthanum hydroxide
Co containing LDHs	Cobalt iron nickel hydroxide
Co containing LDHs	Cobalt iron carbonate hydroxide (Co0.66Fe0.34(CO3)0.17(OH)2)
Co containing LDHs	Cobalt copper zinc hydroxide
Co containing LDHs	Cobalt copper iron manganese nickel hydroxide
Co containing LDHs	Cobalt copper hydroxide (CoCu(OH)2)
Al containing LDHs	Aluminum magnesium hydroxide
Al containing LDHs	Aluminum nickel hydroxide
Al containing LDHs	Aluminum cobalt hydroxide
Al containing LDHs	Aluminum zinc hydroxide
Al containing LDHs	Aluminum cobalt nickel hydroxide
Al containing LDHs	Aluminum hydroxide oxide
Al containing LDHs	Aluminum hydroxide
Al containing LDHs	Aluminum cobalt lanthanum hydroxide
Al containing LDHs	Aluminum zinc hydroxide nitrate
Al containing LDHs	Aluminum zinc hydroxide (AlZn(OH)2)
Al containing LDHs	Aluminum nickel carbonate hydroxide
Al containing LDHs	Aluminum magnesium nickel hydroxide
Al containing LDHs	Aluminum iron nickel hydroxide
Al containing LDHs	Aluminum hydroxide (Al(OH)2)

Al containing LDHs	Aluminum cobalt zinc hydroxide
Al containing LDHs	Aluminum cobalt carbonate hydroxide (Al2Co6(CO3)(OH)6)
Al containing LDHs	Aluminum chromium nickel hydroxide
Fe containing LDHs	Iron nickel hydroxide
Fe containing LDHs	Iron hydroxide
Fe containing LDHs	Iron zinc hydroxide
Fe containing LDHs	Iron nickel hydroxide (FeNi(OH))
Fe containing LDHs	Iron sodium carbonate hydroxide (Fe2Na6(CO3)(OH)16)
Fe containing LDHs	Iron nickel zinc hydroxide
Fe containing LDHs	Iron nickel hydroxide oxide
Fe containing LDHs	Iron nickel hydroxide (Fe0.25Ni0.75(OH)2)
Fe containing LDHs	Iron magnesium zinc hydroxide
Fe containing LDHs	Iron magnesium nickel hydroxide
Fe containing LDHs	Iron magnesium hydroxide
Fe containing LDHs	Carbon iron magnesium chloride hydroxide oxide (C0.2FeMg2Cl0.6(OH)6O0.2)
Zn contaiing LDHs	Zinc hydroxide
Mg containing LDHs	Magnesium hydroxide
Mg containing LDHs	Magnesium manganese hydroxide
Mg containing LDHs	Magnesium nickel hydroxide
Mn containing LDHs	Manganese nickel hydroxide
Mn containing LDHs	Manganese nickel zinc hydroxide
Mn containing LDHs	Manganese nickel hydroxide (MnNi(OH)2)
Mn containing LDHs	Manganese hydroxide
Cu containing LDHs	Copper hydroxide
Cu containing LDHs	Copper nickel hydroxide
Cu containing LDHs	Copper iron hydroxide
Others	Titanium zinc hydroxide
Others	Gallium nickel hydroxide
Others	Chromium cobalt iron hydroxide
Bi containing LDHs	Bismuth nickel hydroxide

**Table S8: List of substances grouped in 2D perovskites by various parameters**	
**Table S8A: Perovskites grouped by metal**	
**Perovskites Metal group**	**Substance Name**
Lead perovskites	Barium cesium lead bromide (Ba2CsPb2Br9)
Lead perovskites	Barium cesium lead chloride (Ba2CsPb2Cl9)
Lead perovskites	Barium cesium lead iodide (Ba2CsPb2I9)
Lead perovskites	Barium lead iodide (Ba2PbI6)
Lead perovskites	Cesium lead bromide iodide (CsPb2BrI4)

Lead perovskites	Cesium lead iodide (CsPb2I7)
Lead perovskites	Plumbate(2-), bis(1-butanamine)tetraiodo-, hydrogen (1:2)
Lead perovskites	Plumbate(2-), bromotrichloro-, (T-4)-, dihydrogen, compd. with benzeneethanamine (1:2)
Lead perovskites	Plumbate(2-), bromotrichloro-, (T-4)-, hydrogen, compd. with benzenemethanamine (1:2:2)
Lead perovskites	Plumbate(2-), bromotriiodo-, (T-4)-, dihydrogen, compd. with benzeneethanamine (1:2)
Lead perovskites	Plumbate(2-), chlorotriiodo-, (T-4)-, hydrogen, compd. with methanamine (1:2:2)
Lead perovskites	Plumbate(2-), dibromodichloro-, (T-4)-, dihydrogen, compd. with benzeneethanamine (1:2)
Lead perovskites	Plumbate(2-), dibromodichloro-, (T-4)-, hydrogen, compd. with benzenemethanamine (1:2:2)
Lead perovskites	Plumbate(2-), dibromodiiodo-, (T-4)-, dihydrogen, compd. with benzeneethanamine (1:2)
Lead perovskites	Plumbate(2-), tetrabromo-, (T-4)-, dihydrogen, compd. with 1H-imidazole-1-propanamine (1:1)
Lead perovskites	Plumbate(2-), tetrabromo-, (T-4)-, dihydrogen, compd. with 2-propyn-1-amine (1:2)
Lead perovskites	Plumbate(2-), tetrabromo-, (T-4)-, hydrogen, compd. with 1-butanamine (1:2:2)
Lead perovskites	Plumbate(2-), tetrabromo-, (T-4)-, hydrogen, compd. with 1-cyclohexene-1-ethanamine (1:2:2)
Lead perovskites	Plumbate(2-), tetrabromo-, (T-4)-, hydrogen, compd. with 1-hexanamine (1:2:2)
Lead perovskites	Plumbate(2-), tetrabromo-, (T-4)-, hydrogen, compd. with 1-octanamine (1:2:2)
Lead perovskites	Plumbate(2-), tetrabromo-, (T-4)-, hydrogen, compd. with 1,4-butanediamine (1:2:2)
Lead perovskites	Plumbate(2-), tetrabromo-, (T-4)-, hydrogen, compd. with 2-naphthaleneethanamine (1:2:2)
Lead perovskites	Plumbate(2-), tetrabromo-, (T-4)-, hydrogen, compd. with 2-naphthalenemethanamine (1:2:2)
Lead perovskites	Plumbate(2-), tetrabromo-, (T-4)-, hydrogen, compd. with 3,5-decadiyn-1-amine (1:2:2)

Lead perovskites	Plumbate(2-), tetrabromo-, (T-4)-, hydrogen, compd. with 4-fluorobenzeneethanamine (1:2:2)
Lead perovskites	Plumbate(2-), tetrabromo-, (T-4)-, hydrogen, compd. with (αR)-4-fluoro-α-methylbenzenemethanamine (1:2:2)
Lead perovskites	Plumbate(2-), tetrabromo-, (T-4)-, hydrogen, compd. with benzeneethanamine (1:2:2)
Lead perovskites	Plumbate(2-), tetrabromo-, (T-4)-, hydrogen, compd. with benzenemethanamine (1:2:2)
Lead perovskites	Plumbate(2-), tetrabromo-, (T-4)-, hydrogen, compd. with cyclohexanamine (1:2:2)
Lead perovskites	Plumbate(2-), tetrabromo-, (T-4)-, hydrogen, compd. with ethanamine (1:2:2)
Lead perovskites	Plumbate(2-), tetrabromo-, (T-4)-, hydrogen, compd. with methylhydrazine (1:2:2)
Lead perovskites	Plumbate(2-), tetrabromo-, (T-4)-, hydrogen, compd. with tetrahydro-2H-pyran-4-amine (1:2:2)
Lead perovskites	Plumbate(2-), tetrachloro-, (T-4)-, dihydrogen, compd. with benzenemethanamine (1:2)
Lead perovskites	Plumbate(2-), tetrachloro-, (T-4)-, hydrogen, compd. with 1-butanamine (1:2:2)
Lead perovskites	Plumbate(2-), tetrachloro-, (T-4)-, hydrogen, compd. with 2-naphthaleneethanamine (1:2:2)
Lead perovskites	Plumbate(2-), tetrachloro-, (T-4)-, hydrogen, compd. with 2-naphthalenemethanamine (1:2:2)
Lead perovskites	Plumbate(2-), tetrachloro-, (T-4)-, hydrogen, compd. with 4-fluorobenzeneethanamine (1:2:2)
Lead perovskites	Plumbate(2-), tetrachloro-, (T-4)-, hydrogen, compd. with benzeneethanamine (1:2:2)
Lead perovskites	Plumbate(2-), tetraiodo-, (T-4)-, dihydrogen, compd. with 2-aminoethanol (1:2)
Lead perovskites	Plumbate(2-), tetraiodo-, (T-4)-, dihydrogen, compd. with 2,2′-bi-1H-imidazole (1:1)
Lead perovskites	Plumbate(2-), tetraiodo-, (T-4)-, dihydrogen, compd. with 4-fluorobenzenemethanamine (1:2)
Lead perovskites	Plumbate(2-), tetraiodo-, (T-4)-, dihydrogen, compd. with 4-methoxybenzeneethanamine (1:2)
Lead perovskites	Plumbate(2-), tetraiodo-, (T-4)-, dihydrogen, compd. with benzenamine (1:2)
Lead perovskites	Plumbate(2-), tetraiodo-, (T-4)-, dihydrogen, compd. with benzenebutanamine (1:2)
Lead perovskites	Plumbate(2-), tetraiodo-, (T-4)-, dihydrogen, compd. with guanidine (1:2)

Lead perovskites	Plumbate(2-), tetraiodo-, (T-4)-, dihydrogen, compd. with methanamine (1:2)
Lead perovskites	Plumbate(2-), tetraiodo-, (T-4)-, dihydrogen, compd. with α-methylbenzenemethanamine (1:2)
Lead perovskites	Plumbate(2-), tetraiodo-, (T-4)-, hydrogen, compd. with 1-butanamine (1:2:2)
Lead perovskites	Plumbate(2-), tetraiodo-, (T-4)-, hydrogen, compd. with 1-cyclohexene-1-ethanamine (1:2:2)
Lead perovskites	Plumbate(2-), tetraiodo-, (T-4)-, hydrogen, compd. with 1-decanamine (1:2:2)
Lead perovskites	Plumbate(2-), tetraiodo-, (T-4)-, hydrogen, compd. with 1-dodecanamine (1:2:2)
Lead perovskites	Plumbate(2-), tetraiodo-, (T-4)-, hydrogen, compd. with 1-heptanamine (1:2:2)
Lead perovskites	Plumbate(2-), tetraiodo-, (T-4)-, hydrogen, compd. with 1-hexadecanamine (1:2:2)
Lead perovskites	Plumbate(2-), tetraiodo-, (T-4)-, hydrogen, compd. with 1-hexanamine (1:2:2)
Lead perovskites	Plumbate(2-), tetraiodo-, (T-4)-, hydrogen, compd. with 1-naphthalenemethanamine (1:2:2)
Lead perovskites	Plumbate(2-), tetraiodo-, (T-4)-, hydrogen, compd. with 1-nonanamine (1:2:2)
Lead perovskites	Plumbate(2-), tetraiodo-, (T-4)-, hydrogen, compd. with 1-octanamine (1:2:2)
Lead perovskites	Plumbate(2-), tetraiodo-, (T-4)-, hydrogen, compd. with 1-pentanamine (1:2:2)
Lead perovskites	Plumbate(2-), tetraiodo-, (T-4)-, hydrogen, compd. with 1-propanamine (1:2:2)
Lead perovskites	Plumbate(2-), tetraiodo-, (T-4)-, hydrogen, compd. with 1-pyrenebutanamine (1:2:2)
Lead perovskites	Plumbate(2-), tetraiodo-, (T-4)-, hydrogen, compd. with 1-tetradecanamine (1:2:2)
Lead perovskites	Plumbate(2-), tetraiodo-, (T-4)-, hydrogen, compd. with 1-undecanamine (1:2:2)
Lead perovskites	Plumbate(2-), tetraiodo-, (T-4)-, hydrogen, compd. with 1,3,5-hexatriene-1,6-diamine (1:2:1)
Lead perovskites	Plumbate(2-), tetraiodo-, (T-4)-, hydrogen, compd. with 1,4-benzenediamine (1:2:1)
Lead perovskites	Plumbate(2-), tetraiodo-, (T-4)-, hydrogen, compd. with 1,4-benzenedimethanamine (1:2:1)
Lead perovskites	Plumbate(2-), tetraiodo-, (T-4)-, hydrogen, compd. with 1,4-butanediamine (1:2:1)
Lead perovskites	Plumbate(2-), tetraiodo-, (T-4)-, hydrogen, compd. with 1,5-naphthalenediamine (1:2:1)

Lead perovskites	Plumbate(2-), tetraiodo-, (T-4)-, hydrogen, compd. with 1,6-hexanediamine (1:2:1)
Lead perovskites	Plumbate(2-), tetraiodo-, (T-4)-, hydrogen, compd. with 1,7-heptanediamine (1:2:1)
Lead perovskites	Plumbate(2-), tetraiodo-, (T-4)-, hydrogen, compd. with 1,8-octanediamine (1:2:1)
Lead perovskites	Plumbate(2-), tetraiodo-, (T-4)-, hydrogen, compd. with 1H-imidazole-5-ethanamine (1:2:1)
Lead perovskites	Plumbate(2-), tetraiodo-, (T-4)-, hydrogen, compd. with 2-fluorobenzeneethanamine (1:2:2)
Lead perovskites	Plumbate(2-), tetraiodo-, (T-4)-, hydrogen, compd. with 2-methyl-1-propanamine (1:2:2)
Lead perovskites	Plumbate(2-), tetraiodo-, (T-4)-, hydrogen, compd. with 2-naphthaleneethanamine (1:2:2)
Lead perovskites	Plumbate(2-), tetraiodo-, (T-4)-, hydrogen, compd. with 2-naphthalenemethanamine (1:2:2)
Lead perovskites	Plumbate(2-), tetraiodo-, (T-4)-, hydrogen, compd. with 2-pyridineethanamine (1:2:1)
Lead perovskites	Plumbate(2-), tetraiodo-, (T-4)-, hydrogen, compd. with 2-thiopheneethanamine (1:2:2)
Lead perovskites	Plumbate(2-), tetraiodo-, (T-4)-, hydrogen, compd. with 2,2′-(2,5-cyclohexadiene-1,4-diylidene)bis[propanedinitrile] and 1-pyrenebutanamine (1:2:2:2)
Lead perovskites	Plumbate(2-), tetraiodo-, (T-4)-, hydrogen, compd. with 2,2′-[1,2-ethanediylbis(oxy)]bis[ethanamine] (1:2:1)
Lead perovskites	Plumbate(2-), tetraiodo-, (T-4)-, hydrogen, compd. with 2,3,4,5,6-pentafluorobenzeneethanamine (1:2:2)
Lead perovskites	Plumbate(2-), tetraiodo-, (T-4)-, hydrogen, compd. with 3-fluorobenzeneethanamine (1:2:2)
Lead perovskites	Plumbate(2-), tetraiodo-, (T-4)-, hydrogen, compd. with 3-methyl-1-butanamine (1:2:2)
Lead perovskites	Plumbate(2-), tetraiodo-, (T-4)-, hydrogen, compd. with 3-piperidinemethanamine (1:2:1)
Lead perovskites	Plumbate(2-), tetraiodo-, (T-4)-, hydrogen, compd. with 3-pyridineethanamine (1:2:1)
Lead perovskites	Plumbate(2-), tetraiodo-, (T-4)-, hydrogen, compd. with 3-pyridinemethanamine (1:2:1)
Lead perovskites	Plumbate(2-), tetraiodo-, (T-4)-, hydrogen, compd. with 4-(trifluoromethyl)benzeneethanamine (1:2:2)

Lead perovskites	Plumbate(2-), tetraiodo-, (T-4)-, hydrogen, compd. with 4-bromo-α-methylbenzenemethanamine (1:2:2)
Lead perovskites	Plumbate(2-), tetraiodo-, (T-4)-, hydrogen, compd. with 4-fluorobenzeneethanamine (1:2:1)
Lead perovskites	Plumbate(2-), tetraiodo-, (T-4)-, hydrogen, compd. with 4-fluorobenzeneethanamine (1:2:2)
Lead perovskites	Plumbate(2-), tetraiodo-, (T-4)-, hydrogen, compd. with 4-piperidinemethanamine (1:2:1)
Lead perovskites	Plumbate(2-), tetraiodo-, (T-4)-, hydrogen, compd. with 4-pyridineethanamine (1:2:1)
Lead perovskites	Plumbate(2-), tetraiodo-, (T-4)-, hydrogen, compd. with 4-pyridinemethanamine (1:2:1)
Lead perovskites	Plumbate(2-), tetraiodo-, (T-4)-, hydrogen, compd. with 4,4-difluoropiperidine (1:2:2)
Lead perovskites	Plumbate(2-), tetraiodo-, (T-4)-, hydrogen, compd. with (αR)-α-methylbenzenemethanamine (1:2:2)
Lead perovskites	Plumbate(2-), tetraiodo-, (T-4)-, hydrogen, compd. with (αS)-α-methylbenzenemethanamine (1:2:2)
Lead perovskites	Plumbate(2-), tetraiodo-, (T-4)-, hydrogen, compd. with benzeneethanamine (1:2:2)
Lead perovskites	Plumbate(2-), tetraiodo-, (T-4)-, hydrogen, compd. with benzenemethanamine (1:2:2)
Lead perovskites	Plumbate(2-), tetraiodo-, (T-4)-, hydrogen, compd. with cyclohexanamine (1:2:2)
Lead perovskites	Plumbate(2-), tetraiodo-, (T-4)-, hydrogen, compd. with cyclohexaneethanamine (1:2:2)
Lead perovskites	Plumbate(2-), tetraiodo-, (T-4)-, hydrogen, compd. with cyclohexanemethanamine (1:2:2)
Lead perovskites	Plumbate(2-), tetraiodo-, (T-4)-, hydrogen, compd. with cyclopentanamine (1:2:2)
Lead perovskites	Plumbate(2-), tetraiodo-, (T-4)-, hydrogen, compd. with ethanamine (1:2:2)
Lead perovskites	Plumbate(2-), tetraiodo-, (T-4)-, hydrogen, compd. with guanidine and methanimidamide (1:2:1:1)
Lead perovskites	Plumbate(2-), tetraiodo-, (T-4)-, hydrogen, compd. with β-methylbenzeneethanamine (1:2:2)
Lead perovskites	Plumbate(3-), μ-bromohexabromodi-, hydrogen, compd. with 1-butanamine and methanamine (1:3:2:1)
Lead perovskites	Plumbate(3-), μ-bromohexabromodi-, hydrogen, compd. with 1-butanamine and methanimidamide (1:3:2:1)

Lead perovskites	Plumbate(3-), μ-bromohexabromodi-, hydrogen, compd. with 1-heptanamine and methanamine (1:3:2:1)
Lead perovskites	Plumbate(3-), μ-bromohexabromodi-, hydrogen, compd. with 1-hexanamine and methanamine (1:3:2:1)
Lead perovskites	Plumbate(3-), μ-bromohexabromodi-, hydrogen, compd. with methanamine and 1-octanamine (1:3:1:2)
Lead perovskites	Plumbate(3-), μ-bromohexabromodi-, hydrogen, compd. with methanimidamide and 1-naphthalenemethanamine (1:3:1:2)
Lead perovskites	Plumbate(3-), μ-bromohexabromodi-, trihydrogen, compd. with benzeneethanamine and methanamine (1:2:1)
Lead perovskites	Plumbate(3-), μ-iodohexaiododi-, hydrogen, compd. with 1-butanamine and methanamine (1:3:2:1)
Lead perovskites	Plumbate(3-), μ-iodohexaiododi-, hydrogen, compd. with 1-butanamine and methanimidamide (1:3:2:1)
Lead perovskites	Plumbate(3-), μ-iodohexaiododi-, hydrogen, compd. with 1-dodecanamine and methanamine (1:3:2:1)
Lead perovskites	Plumbate(3-), μ-iodohexaiododi-, hydrogen, compd. with 1-hexanamine and methanamine (1:3:2:1)
Lead perovskites	Plumbate(3-), μ-iodohexaiododi-, hydrogen, compd. with 1,4-benzenedimethanamine and methanimidamide (1:3:1:1)
Lead perovskites	Plumbate(3-), μ-iodohexaiododi-, hydrogen, compd. with benzeneethanamine and methanamine (1:3:2:1)
Lead perovskites	Plumbate(3-), μ-iodohexaiododi-, hydrogen, compd. with benzenemethanamine and methanamine (1:3:2:1)
Lead perovskites	Plumbate(3-), μ-iodohexaiododi-, hydrogen, compd. with methanamine and 1-octanamine (1:3:1:2)
Lead perovskites	Plumbate(3-), μ-iodohexaiododi-, hydrogen, compd. with methanamine and 1-tetradecanamine (1:3:1:2)
Lead perovskites	Plumbate(3-), μ-iodohexaiododi-, hydrogen, compd. with methanamine and 1,3-propanediamine (1:3:1:1)
Lead perovskites	Plumbate(3-), μ-iodohexaiododi-, hydrogen, compd. with methanamine and 1,8-octanediamine (1:3:1:1)

Lead perovskites	Plumbate(3-), μ-iodohexaiododi-, hydrogen, compd. with methanamine and 2-methyl-1-propanamine (1:3:1:2)
Lead perovskites	Plumbate(3-), μ-iodohexaiododi-, hydrogen, compd. with methanimidamide and 1-naphthalenemethanamine (1:3:1:2)
Lead perovskites	Plumbate(3-), μ-iodohexaiododi-, hydrogen, compd. with N-methylmethanamine and 1-pentanamine (1:3:1:2)
Lead perovskites	Plumbate(3-), μ-iodohexaiododi-, trihydrogen, compd. with methanamine and 2-thiophenemethanamine (1:1:2)
Lead perovskites	Plumbate(4-), di-μ-bromooctabromotri-, cesium hydrogen, compd. with benzeneethanamine (1:2:2:2)
Lead perovskites	Plumbate(4-), di-μ-bromooctabromotri-, hydrogen, compd. with 1-butanamine, compd. with methanamine (1:4:2:2)
Lead perovskites	Plumbate(4-), di-μ-bromooctabromotri-, hydrogen, compd. with benzeneethanamine and methanamine (1:4:2:2)
Lead perovskites	Plumbate(4-), di-μ-iodooctaiodotri-, hydrogen, compd. with 1-butanamine and ethanamine (1:4:2:2)
Lead perovskites	Plumbate(4-), di-μ-iodooctaiodotri-, hydrogen, compd. with 1-butanamine and methanamine (1:4:2:2)
Lead perovskites	Plumbate(4-), di-μ-iodooctaiodotri-, hydrogen, compd. with 1-butanamine and methanimidamide (1:4:2:2)
Lead perovskites	Plumbate(4-), di-μ-iodooctaiodotri-, hydrogen, compd. with benzenemethanamine and methanamine (1:4:2:2)
Lead perovskites	Plumbate(4-), di-μ-iodooctaiodotri-, hydrogen, compd. with methanamine and 1,3-propanediamine (1:4:2:1)
Lead perovskites	Plumbate(4-), di-μ-iodooctaiodotri-, hydrogen, compd. with methanamine and 1,8-octanediamine (1:4:2:1)
Lead perovskites	Plumbate(4-), di-μ-iodooctaiodotri-, hydrogen, compd. with methanamine and 2-methyl-1-propanamine (1:4:2:2)
Lead perovskites	Plumbate(4-), di-μ-iodooctaiodotri-, hydrogen, compd. with methanamine and 4-piperidinemethanamine (1:4:2:1)
Lead perovskites	Plumbate(4-), hexa-μ-bromotetrabromotri-, hydrogen, compd. with benzeneethanamine and methanimidamide (1:4:2:2)
Lead perovskites	Plumbate(4-), hexa-μ-bromotetrabromotri-, hydrogen, compd. with ethanamine (1:4:4)

Lead perovskites	Plumbate(4-), hexa-μ-iodotetraiodotri-, hydrogen, compd. with 1-butanamine and methanamine (1:4:2:2)
Lead perovskites	Plumbate(4-), hexa-μ-iodotetraiodotri-, hydrogen, compd. with 1-hexanamine and methanamine (1:4:2:2)
Lead perovskites	Plumbate(4-), hexa-μ-iodotetraiodotri-, hydrogen, compd. with 1,4-benzenedimethanamine and methanimidamide (1:4:1:2)
Lead perovskites	Plumbate(4-), hexa-μ-iodotetraiodotri-, hydrogen, compd. with 1,4-butanediamine and methanamine (2:8:1:6)
Lead perovskites	Plumbate(4-), hexaiodo-, (OC-6-11)-, hydrogen, compd. with benzeneethanamine (1:4:4)
Lead perovskites	Plumbate(4-), hexaiodo-, tetracesium, (OC-6-11)-
Lead perovskites	Plumbate(4-), tri-μ-iododi-μ3-iodopentaiodotri-, hydrogen, compd. with 1-butanamine and methanamine (1:4:2:2)
Lead perovskites	Plumbate(4-), tri-μ-iodoheptaiodotri-, hydrogen, compd. with benzeneethanamine and methanamine (1:4:2:2)
Lead perovskites	Plumbate(5-), tri-μ-bromodecabromotetra-, cesium hydrogen, compd. with benzeneethanamine (1:3:2:2)
Lead perovskites	Plumbate(5-), tri-μ-bromodecabromotetra-, hydrogen, compd. with 1-butanamine and methanamine (1:5:2:3)
Lead perovskites	Plumbate(5-), tri-μ-bromodecabromotetra-, hydrogen, compd. with benzeneethanamine and 2-propanamine (1:5:2:3)
Lead perovskites	Plumbate(5-), tri-μ-iododecaiodotetra-, hydrogen, compd. with 1-butanamine and methanamine (1:5:2:3)
Lead perovskites	Plumbate(5-), tri-μ-iododecaiodotetra-, hydrogen, compd. with 1,4-benzenedimethanamine and methanimidamide (1:5:1:3)
Lead perovskites	Plumbate(5-), tri-μ-iododecaiodotetra-, hydrogen, compd. with benzeneethanamine and methanamine (1:5:2:3)
Lead perovskites	Plumbate(5-), tri-μ-iododecaiodotetra-, hydrogen, compd. with methanamine and 1,3-propanediamine (1:5:3:1)
Lead perovskites	Plumbate(5-), tri-μ-iododecaiodotetra-, hydrogen, compd. with methanamine and 1,8-octanediamine (1:5:3:1)

Lead perovskites	Plumbate(5-), tri-μ-iododecaiodotetra-, hydrogen, compd. with methanamine and 2-methyl-1-propanamine (1:5:3:2)
Lead perovskites	Plumbate(5-), tri-μ-iododecaiodotetra-, hydrogen, compd. with methanamine and 3-pyridinemethanamine (1:5:3:1)
Lead perovskites	Plumbate(5-), tri-μ-iododecaiodotetra-, hydrogen, compd. with methanamine and 4-pyridinemethanamine (1:5:3:1)
Lead perovskites	Plumbate(5-), μ-bromooctabromodi-, hydrogen, compd. with 1,2-ethanediamine hydrobromide (1:5:4:3)
Lead perovskites	Plumbate(6-), tetra-μ-iodododecaiodopenta-, hydrogen, compd. with 1-butanamine and methanamine (1:6:2:4)
Lead perovskites	Plumbate(6-), tetra-μ-iodododecaiodopenta-, hydrogen, compd. with 4-fluorobenzeneethanamine and methanamine (1:6:2:4)
Lead perovskites	Plumbate(6-), tetra-μ-iodododecaiodopenta-, hydrogen, compd. with benzeneethanamine and methanamine (1:6:2:4)
Lead perovskites	Plumbate(6-), tetra-μ-iodododecaiodopenta-, hydrogen, compd. with methanamine and 1-octanamine (1:6:4:2)
Lead perovskites	Plumbate(6-), tetra-μ-iodododecaiodopenta-, hydrogen, compd. with methanamine and 1-propanamine (1:6:4:2)
Lead perovskites	Plumbate(6-), tetra-μ-iodododecaiodopenta-, hydrogen, compd. with methanamine and 2-methyl-1-propanamine (1:6:4:2)
Lead perovskites	β-Alanine, (T-4)-tetraiodoplumbate(2-) (2:1)
Tin perovskites	Benzeneethanamine, (T-4)-tetraiodostannate(2-) (2:1)
Tin perovskites	Stannate(2-), tetrabromo-, (T-4)-, dihydrogen, compd. with benzeneethanamine (1:2)
Tin perovskites	Stannate(2-), tetrabromo-, (T-4)-, hydrogen, compd. with 1-butanamine (1:2:2)
Tin perovskites	Stannate(2-), tetrabromo-, (T-4)-, hydrogen, compd. with 1-dodecanamine (1:2:2)
Tin perovskites	Stannate(2-), tetrabromo-, (T-4)-, hydrogen, compd. with 1-octadecanamine (1:2:2)
Tin perovskites	Stannate(2-), tetrabromo-, (T-4)-, hydrogen, compd. with 1-octanamine (1:2:2)
Tin perovskites	Stannate(2-), tetrabromo-, (T-4)-, hydrogen, compd. with 1,8-octanediamine (1:2:1)
Tin perovskites	Stannate(2-), tetrachloro-, (T-4)-, dihydrogen, compd. with 1-butanamine (1:2)
Tin perovskites	Stannate(2-), tetraiodo-, (T-4)-, dihydrogen, compd. with 1,4-butanediamine (1:1)

Tin perovskites	Stannate(2-), tetraiodo-, (T-4)-, dihydrogen, compd. with 4-fluorobenzeneethanamine (1:2)
Tin perovskites	Stannate(2-), tetraiodo-, (T-4)-, hydrogen (T-4)-tetraiodogermanate(2-), compd. with benzeneethanamine (1:4:1:2)
Tin perovskites	Stannate(2-), tetraiodo-, (T-4)-, hydrogen (T-4)-tetraiodogermanate(2-), compd. with benzeneethanamine (1:8:3:2)
Tin perovskites	Stannate(2-), tetraiodo-, (T-4)-, hydrogen (T-4)-tetraiodogermanate(2-), compd. with benzeneethanamine (1:16:7:2)
Tin perovskites	Stannate(2-), tetraiodo-, (T-4)-, hydrogen, compd. with 1-butanamine (1:2:2)
Tin perovskites	Stannate(2-), tetraiodo-, (T-4)-, hydrogen, compd. with 1-hexanamine (1:2:2)
Tin perovskites	Stannate(2-), tetraiodo-, (T-4)-, hydrogen, compd. with 1-octanamine (1:2:2)
Tin perovskites	Stannate(2-), tetraiodo-, (T-4)-, hydrogen, compd. with 2-thiopheneethanamine (1:2:2)

Table S8B: Perovskites grouped by phase

**Perovskite Phase group**	**Substance Name**
Dion-Jacobson	Plumbate(2-), tetrabromo-, (T-4)-, dihydrogen, compd. with 1H-imidazole-1-propanamine (1:1)
Dion-Jacobson	Plumbate(2-), tetrabromo-, (T-4)-, hydrogen, compd. with 1,4-butanediamine (1:2:2)
Dion-Jacobson	Plumbate(2-), tetraiodo-, (T-4)-, dihydrogen, compd. with 2,2′-bi-1H-imidazole (1:1)
Dion-Jacobson	Plumbate(2-), tetraiodo-, (T-4)-, hydrogen, compd. with 1,3,5-hexatriene-1,6-diamine (1:2:1)
Dion-Jacobson	Plumbate(2-), tetraiodo-, (T-4)-, hydrogen, compd. with 1,4-benzenediamine (1:2:1)
Dion-Jacobson	Plumbate(2-), tetraiodo-, (T-4)-, hydrogen, compd. with 1,4-benzenedimethanamine (1:2:1)
Dion-Jacobson	Plumbate(2-), tetraiodo-, (T-4)-, hydrogen, compd. with 1,4-butanediamine (1:2:1)
Dion-Jacobson	Plumbate(2-), tetraiodo-, (T-4)-, hydrogen, compd. with 1,5-naphthalenediamine (1:2:1)
Dion-Jacobson	Plumbate(2-), tetraiodo-, (T-4)-, hydrogen, compd. with 1,6-hexanediamine (1:2:1)
Dion-Jacobson	Plumbate(2-), tetraiodo-, (T-4)-, hydrogen, compd. with 1,7-heptanediamine (1:2:1)
Dion-Jacobson	Plumbate(2-), tetraiodo-, (T-4)-, hydrogen, compd. with 1,8-octanediamine (1:2:1)
Dion-Jacobson	Plumbate(2-), tetraiodo-, (T-4)-, hydrogen, compd. with 1H-imidazole-5-ethanamine (1:2:1)

Dion-Jacobson	Plumbate(2-), tetraiodo-, (T-4)-, hydrogen, compd. with 2-pyridineethanamine (1:2:1)
Dion-Jacobson	Plumbate(2-), tetraiodo-, (T-4)-, hydrogen, compd. with 2,2′-[1,2-ethanediylbis(oxy)]bis[ethanamine] (1:2:1)
Dion-Jacobson	Plumbate(2-), tetraiodo-, (T-4)-, hydrogen, compd. with 3-piperidinemethanamine (1:2:1)
Dion-Jacobson	Plumbate(2-), tetraiodo-, (T-4)-, hydrogen, compd. with 3-pyridinemethanamine (1:2:1)
Dion-Jacobson	Plumbate(2-), tetraiodo-, (T-4)-, hydrogen, compd. with 4-fluorobenzeneethanamine (1:2:1)
Dion-Jacobson	Plumbate(2-), tetraiodo-, (T-4)-, hydrogen, compd. with 4-piperidinemethanamine (1:2:1)
Dion-Jacobson	Plumbate(2-), tetraiodo-, (T-4)-, hydrogen, compd. with 4-pyridineethanamine (1:2:1)
Dion-Jacobson	Plumbate(2-), tetraiodo-, (T-4)-, hydrogen, compd. with 4-pyridinemethanamine (1:2:1)
Dion-Jacobson	Plumbate(3-), μ-iodohexaiododi-, hydrogen, compd. with 1,4-benzenedimethanamine and methanimidamide (1:3:1:1)
Dion-Jacobson	Plumbate(3-), μ-iodohexaiododi-, hydrogen, compd. with methanamine and 1,3-propanediamine (1:3:1:1)
Dion-Jacobson	Plumbate(3-), μ-iodohexaiododi-, hydrogen, compd. with methanamine and 1,8-octanediamine (1:3:1:1)
Dion-Jacobson	Plumbate(4-), di-μ-iodooctaiodotri-, hydrogen, compd. with methanamine and 1,3-propanediamine (1:4:2:1)
Dion-Jacobson	Plumbate(4-), di-μ-iodooctaiodotri-, hydrogen, compd. with methanamine and 1,8-octanediamine (1:4:2:1)
Dion-Jacobson	Plumbate(4-), di-μ-iodooctaiodotri-, hydrogen, compd. with methanamine and 4-piperidinemethanamine (1:4:2:1)
Dion-Jacobson	Plumbate(4-), hexa-μ-iodotetraiodotri-, hydrogen, compd. with 1,4-benzenedimethanamine and methanimidamide (1:4:1:2)
Dion-Jacobson	Plumbate(4-), hexa-μ-iodotetraiodotri-, hydrogen, compd. with 1,4-butanediamine and methanamine (2:8:1:6)
Dion-Jacobson	Plumbate(5-), tri-μ-iododecaiodotetra-, hydrogen, compd. with 1,4-benzenedimethanamine and methanimidamide (1:5:1:3)
Dion-Jacobson	Plumbate(5-), tri-μ-iododecaiodotetra-, hydrogen, compd. with methanamine and 1,3-propanediamine (1:5:3:1)

Dion-Jacobson	Plumbate(5-), tri-μ-iododecaiodotetra-, hydrogen, compd. with methanamine and 1,8-octanediamine (1:5:3:1)
Dion-Jacobson	Plumbate(5-), tri-μ-iododecaiodotetra-, hydrogen, compd. with methanamine and 3-pyridinemethanamine (1:5:3:1)
Dion-Jacobson	Plumbate(5-), tri-μ-iododecaiodotetra-, hydrogen, compd. with methanamine and 4-pyridinemethanamine (1:5:3:1)
Dion-Jacobson	Plumbate(5-), μ-bromooctabromodi-, hydrogen, compd. with 1,2-ethanediamine hydrobromide (1:5:4:3)
Dion-Jacobson	Stannate(2-), tetrabromo-, (T-4)-, hydrogen, compd. with 1,8-octanediamine (1:2:1)
Dion-Jacobson	Stannate(2-), tetraiodo-, (T-4)-, dihydrogen, compd. with 1,4-butanediamine (1:1)
Ruddlesden-Popper	Barium cesium lead bromide (Ba2CsPb2Br9)
Ruddlesden-Popper	Barium cesium lead chloride (Ba2CsPb2Cl9)
Ruddlesden-Popper	Barium cesium lead iodide (Ba2CsPb2I9)
Ruddlesden-Popper	Barium lead iodide (Ba2PbI6)
Ruddlesden-Popper	Benzeneethanamine, (T-4)-tetraiodostannate(2-) (2:1)
Ruddlesden-Popper	Cesium lead bromide iodide (CsPb2BrI4)
Ruddlesden-Popper	Cesium lead iodide (CsPb2I7)
Ruddlesden-Popper	Plumbate(2-), bis(1-butanamine)tetraiodo-, hydrogen (1:2)
Ruddlesden-Popper	Plumbate(2-), bromotrichloro-, (T-4)-, dihydrogen, compd. with benzeneethanamine (1:2)
Ruddlesden-Popper	Plumbate(2-), bromotrichloro-, (T-4)-, hydrogen, compd. with benzenemethanamine (1:2:2)
Ruddlesden-Popper	Plumbate(2-), bromotriiodo-, (T-4)-, dihydrogen, compd. with benzeneethanamine (1:2)
Ruddlesden-Popper	Plumbate(2-), chlorotriiodo-, (T-4)-, hydrogen, compd. with methanamine (1:2:2)
Ruddlesden-Popper	Plumbate(2-), dibromodichloro-, (T-4)-, dihydrogen, compd. with benzeneethanamine (1:2)
Ruddlesden-Popper	Plumbate(2-), dibromodichloro-, (T-4)-, hydrogen, compd. with benzenemethanamine (1:2:2)
Ruddlesden-Popper	Plumbate(2-), dibromodiiodo-, (T-4)-, dihydrogen, compd. with benzeneethanamine (1:2)
Ruddlesden-Popper	Plumbate(2-), tetrabromo-, (T-4)-, dihydrogen, compd. with 2-propyn-1-amine (1:2)

Ruddlesden-Popper	Plumbate(2-), tetrabromo-, (T-4)-, hydrogen, compd. with 1-butanamine (1:2:2)
Ruddlesden-Popper	Plumbate(2-), tetrabromo-, (T-4)-, hydrogen, compd. with 1-cyclohexene-1-ethanamine (1:2:2)
Ruddlesden-Popper	Plumbate(2-), tetrabromo-, (T-4)-, hydrogen, compd. with 1-hexanamine (1:2:2)
Ruddlesden-Popper	Plumbate(2-), tetrabromo-, (T-4)-, hydrogen, compd. with 1-octanamine (1:2:2)
Ruddlesden-Popper	Plumbate(2-), tetrabromo-, (T-4)-, hydrogen, compd. with 2-naphthaleneethanamine (1:2:2)
Ruddlesden-Popper	Plumbate(2-), tetrabromo-, (T-4)-, hydrogen, compd. with 2-naphthalenemethanamine (1:2:2)
Ruddlesden-Popper	Plumbate(2-), tetrabromo-, (T-4)-, hydrogen, compd. with 3,5-decadiyn-1-amine (1:2:2)
Ruddlesden-Popper	Plumbate(2-), tetrabromo-, (T-4)-, hydrogen, compd. with 4-fluorobenzeneethanamine (1:2:2)
Ruddlesden-Popper	Plumbate(2-), tetrabromo-, (T-4)-, hydrogen, compd. with (αR)-4-fluoro-α-methylbenzenemethanamine (1:2:2)
Ruddlesden-Popper	Plumbate(2-), tetrabromo-, (T-4)-, hydrogen, compd. with benzeneethanamine (1:2:2)
Ruddlesden-Popper	Plumbate(2-), tetrabromo-, (T-4)-, hydrogen, compd. with benzenemethanamine (1:2:2)
Ruddlesden-Popper	Plumbate(2-), tetrabromo-, (T-4)-, hydrogen, compd. with cyclohexanamine (1:2:2)
Ruddlesden-Popper	Plumbate(2-), tetrabromo-, (T-4)-, hydrogen, compd. with ethanamine (1:2:2)
Ruddlesden-Popper	Plumbate(2-), tetrabromo-, (T-4)-, hydrogen, compd. with methylhydrazine (1:2:2)
Ruddlesden-Popper	Plumbate(2-), tetrabromo-, (T-4)-, hydrogen, compd. with tetrahydro-2H-pyran-4-amine (1:2:2)
Ruddlesden-Popper	Plumbate(2-), tetrachloro-, (T-4)-, dihydrogen, compd. with benzenemethanamine (1:2)
Ruddlesden-Popper	Plumbate(2-), tetrachloro-, (T-4)-, hydrogen, compd. with 1-butanamine (1:2:2)
Ruddlesden-Popper	Plumbate(2-), tetrachloro-, (T-4)-, hydrogen, compd. with 2-naphthaleneethanamine (1:2:2)
Ruddlesden-Popper	Plumbate(2-), tetrachloro-, (T-4)-, hydrogen, compd. with 2-naphthalenemethanamine (1:2:2)
Ruddlesden-Popper	Plumbate(2-), tetrachloro-, (T-4)-, hydrogen, compd. with 4-fluorobenzeneethanamine (1:2:2)

Ruddlesden-Popper	Plumbate(2-), tetrachloro-, (T-4)-, hydrogen, compd. with benzeneethanamine (1:2:2)
Ruddlesden-Popper	Plumbate(2-), tetraiodo-, (T-4)-, dihydrogen, compd. with 2-aminoethanol (1:2)
Ruddlesden-Popper	Plumbate(2-), tetraiodo-, (T-4)-, dihydrogen, compd. with 4-fluorobenzenemethanamine (1:2)
Ruddlesden-Popper	Plumbate(2-), tetraiodo-, (T-4)-, dihydrogen, compd. with 4-methoxybenzeneethanamine (1:2)
Ruddlesden-Popper	Plumbate(2-), tetraiodo-, (T-4)-, dihydrogen, compd. with benzenamine (1:2)
Ruddlesden-Popper	Plumbate(2-), tetraiodo-, (T-4)-, dihydrogen, compd. with benzenebutanamine (1:2)
Ruddlesden-Popper	Plumbate(2-), tetraiodo-, (T-4)-, dihydrogen, compd. with guanidine (1:2)
Ruddlesden-Popper	Plumbate(2-), tetraiodo-, (T-4)-, dihydrogen, compd. with methanamine (1:2)
Ruddlesden-Popper	Plumbate(2-), tetraiodo-, (T-4)-, dihydrogen, compd. with α-methylbenzenemethanamine (1:2)
Ruddlesden-Popper	Plumbate(2-), tetraiodo-, (T-4)-, hydrogen, compd. with 1-butanamine (1:2:2)
Ruddlesden-Popper	Plumbate(2-), tetraiodo-, (T-4)-, hydrogen, compd. with 1-cyclohexene-1-ethanamine (1:2:2)
Ruddlesden-Popper	Plumbate(2-), tetraiodo-, (T-4)-, hydrogen, compd. with 1-decanamine (1:2:2)
Ruddlesden-Popper	Plumbate(2-), tetraiodo-, (T-4)-, hydrogen, compd. with 1-dodecanamine (1:2:2)
Ruddlesden-Popper	Plumbate(2-), tetraiodo-, (T-4)-, hydrogen, compd. with 1-heptanamine (1:2:2)
Ruddlesden-Popper	Plumbate(2-), tetraiodo-, (T-4)-, hydrogen, compd. with 1-hexadecanamine (1:2:2)
Ruddlesden-Popper	Plumbate(2-), tetraiodo-, (T-4)-, hydrogen, compd. with 1-hexanamine (1:2:2)
Ruddlesden-Popper	Plumbate(2-), tetraiodo-, (T-4)-, hydrogen, compd. with 1-naphthalenemethanamine (1:2:2)
Ruddlesden-Popper	Plumbate(2-), tetraiodo-, (T-4)-, hydrogen, compd. with 1-nonanamine (1:2:2)
Ruddlesden-Popper	Plumbate(2-), tetraiodo-, (T-4)-, hydrogen, compd. with 1-octanamine (1:2:2)
Ruddlesden-Popper	Plumbate(2-), tetraiodo-, (T-4)-, hydrogen, compd. with 1-pentanamine (1:2:2)
Ruddlesden-Popper	Plumbate(2-), tetraiodo-, (T-4)-, hydrogen, compd. with 1-propanamine (1:2:2)
Ruddlesden-Popper	Plumbate(2-), tetraiodo-, (T-4)-, hydrogen, compd. with 1-pyrenebutanamine (1:2:2)

Ruddlesden-Popper	Plumbate(2-), tetraiodo-, (T-4)-, hydrogen, compd. with 1-tetradecanamine (1:2:2)
Ruddlesden-Popper	Plumbate(2-), tetraiodo-, (T-4)-, hydrogen, compd. with 1-undecanamine (1:2:2)
Ruddlesden-Popper	Plumbate(2-), tetraiodo-, (T-4)-, hydrogen, compd. with 2-fluorobenzeneethanamine (1:2:2)
Ruddlesden-Popper	Plumbate(2-), tetraiodo-, (T-4)-, hydrogen, compd. with 2-methyl-1-propanamine (1:2:2)
Ruddlesden-Popper	Plumbate(2-), tetraiodo-, (T-4)-, hydrogen, compd. with 2-naphthaleneethanamine (1:2:2)
Ruddlesden-Popper	Plumbate(2-), tetraiodo-, (T-4)-, hydrogen, compd. with 2-naphthalenemethanamine (1:2:2)
Ruddlesden-Popper	Plumbate(2-), tetraiodo-, (T-4)-, hydrogen, compd. with 2-thiopheneethanamine (1:2:2)
Ruddlesden-Popper	Plumbate(2-), tetraiodo-, (T-4)-, hydrogen, compd. with 2,2′-(2,5-cyclohexadiene-1,4-diylidene)bis[propanedinitrile] and 1-pyrenebutanamine (1:2:2:2)
Ruddlesden-Popper	Plumbate(2-), tetraiodo-, (T-4)-, hydrogen, compd. with 2,3,4,5,6-pentafluorobenzeneethanamine (1:2:2)
Ruddlesden-Popper	Plumbate(2-), tetraiodo-, (T-4)-, hydrogen, compd. with 3-fluorobenzeneethanamine (1:2:2)
Ruddlesden-Popper	Plumbate(2-), tetraiodo-, (T-4)-, hydrogen, compd. with 3-methyl-1-butanamine (1:2:2)
Ruddlesden-Popper	Plumbate(2-), tetraiodo-, (T-4)-, hydrogen, compd. with 4-(trifluoromethyl)benzeneethanamine (1:2:2)
Ruddlesden-Popper	Plumbate(2-), tetraiodo-, (T-4)-, hydrogen, compd. with 4-bromo-α-methylbenzenemethanamine (1:2:2)
Ruddlesden-Popper	Plumbate(2-), tetraiodo-, (T-4)-, hydrogen, compd. with 4-fluorobenzeneethanamine (1:2:2)
Ruddlesden-Popper	Plumbate(2-), tetraiodo-, (T-4)-, hydrogen, compd. with 4,4-difluoropiperidine (1:2:2)
Ruddlesden-Popper	Plumbate(2-), tetraiodo-, (T-4)-, hydrogen, compd. with (αR)-α-methylbenzenemethanamine (1:2:2)
Ruddlesden-Popper	Plumbate(2-), tetraiodo-, (T-4)-, hydrogen, compd. with (αS)-α-methylbenzenemethanamine (1:2:2)
Ruddlesden-Popper	Plumbate(2-), tetraiodo-, (T-4)-, hydrogen, compd. with benzeneethanamine (1:2:2)
Ruddlesden-Popper	Plumbate(2-), tetraiodo-, (T-4)-, hydrogen, compd. with benzenemethanamine (1:2:2)

Ruddlesden-Popper	Plumbate(2-), tetraiodo-, (T-4)-, hydrogen, compd. with cyclohexanamine (1:2:2)
Ruddlesden-Popper	Plumbate(2-), tetraiodo-, (T-4)-, hydrogen, compd. with cyclohexaneethanamine (1:2:2)
Ruddlesden-Popper	Plumbate(2-), tetraiodo-, (T-4)-, hydrogen, compd. with cyclohexanemethanamine (1:2:2)
Ruddlesden-Popper	Plumbate(2-), tetraiodo-, (T-4)-, hydrogen, compd. with cyclopentanamine (1:2:2)
Ruddlesden-Popper	Plumbate(2-), tetraiodo-, (T-4)-, hydrogen, compd. with ethanamine (1:2:2)
Ruddlesden-Popper	Plumbate(2-), tetraiodo-, (T-4)-, hydrogen, compd. with guanidine and methanimidamide (1:2:1:1)
Ruddlesden-Popper	Plumbate(2-), tetraiodo-, (T-4)-, hydrogen, compd. with β-methylbenzeneethanamine (1:2:2)
Ruddlesden-Popper	Plumbate(3-), μ-bromohexabromodi-, hydrogen, compd. with 1-butanamine and methanamine (1:3:2:1)
Ruddlesden-Popper	Plumbate(3-), μ-bromohexabromodi-, hydrogen, compd. with 1-butanamine and methanimidamide (1:3:2:1)
Ruddlesden-Popper	Plumbate(3-), μ-bromohexabromodi-, hydrogen, compd. with 1-heptanamine and methanamine (1:3:2:1)
Ruddlesden-Popper	Plumbate(3-), μ-bromohexabromodi-, hydrogen, compd. with 1-hexanamine and methanamine (1:3:2:1)
Ruddlesden-Popper	Plumbate(3-), μ-bromohexabromodi-, hydrogen, compd. with methanamine and 1-octanamine (1:3:1:2)
Ruddlesden-Popper	Plumbate(3-), μ-bromohexabromodi-, hydrogen, compd. with methanimidamide and 1-naphthalenemethanamine (1:3:1:2)
Ruddlesden-Popper	Plumbate(3-), μ-bromohexabromodi-, trihydrogen, compd. with benzeneethanamine and methanamine (1:2:1)
Ruddlesden-Popper	Plumbate(3-), μ-iodohexaiododi-, hydrogen, compd. with 1-butanamine and methanamine (1:3:2:1)
Ruddlesden-Popper	Plumbate(3-), μ-iodohexaiododi-, hydrogen, compd. with 1-butanamine and methanimidamide (1:3:2:1)
Ruddlesden-Popper	Plumbate(3-), μ-iodohexaiododi-, hydrogen, compd. with 1-dodecanamine and methanamine (1:3:2:1)
Ruddlesden-Popper	Plumbate(3-), μ-iodohexaiododi-, hydrogen, compd. with 1-hexanamine and methanamine (1:3:2:1)

Ruddlesden-Popper	Plumbate(3-), μ-iodohexaiododi-, hydrogen, compd. with benzeneethanamine and methanamine (1:3:2:1)
Ruddlesden-Popper	Plumbate(3-), μ-iodohexaiododi-, hydrogen, compd. with benzenemethanamine and methanamine (1:3:2:1)
Ruddlesden-Popper	Plumbate(3-), μ-iodohexaiododi-, hydrogen, compd. with methanamine and 1-octanamine (1:3:1:2)
Ruddlesden-Popper	Plumbate(3-), μ-iodohexaiododi-, hydrogen, compd. with methanamine and 1-tetradecanamine (1:3:1:2)
Ruddlesden-Popper	Plumbate(3-), μ-iodohexaiododi-, hydrogen, compd. with methanamine and 2-methyl-1-propanamine (1:3:1:2)
Ruddlesden-Popper	Plumbate(3-), μ-iodohexaiododi-, hydrogen, compd. with methanimidamide and 1-naphthalenemethanamine (1:3:1:2)
Ruddlesden-Popper	Plumbate(3-), μ-iodohexaiododi-, hydrogen, compd. with N-methylmethanamine and 1-pentanamine (1:3:1:2)
Ruddlesden-Popper	Plumbate(3-), μ-iodohexaiododi-, trihydrogen, compd. with methanamine and 2-thiophenemethanamine (1:1:2)
Ruddlesden-Popper	Plumbate(4-), di-μ-bromooctabromotri-, cesium hydrogen, compd. with benzeneethanamine (1:2:2:2)
Ruddlesden-Popper	Plumbate(4-), di-μ-bromooctabromotri-, hydrogen, compd. with 1-butanamine, compd. with methanamine (1:4:2:2)
Ruddlesden-Popper	Plumbate(4-), di-μ-bromooctabromotri-, hydrogen, compd. with benzeneethanamine and methanamine (1:4:2:2)
Ruddlesden-Popper	Plumbate(4-), di-μ-iodooctaiodotri-, hydrogen, compd. with 1-butanamine and ethanamine (1:4:2:2)
Ruddlesden-Popper	Plumbate(4-), di-μ-iodooctaiodotri-, hydrogen, compd. with 1-butanamine and methanamine (1:4:2:2)
Ruddlesden-Popper	Plumbate(4-), di-μ-iodooctaiodotri-, hydrogen, compd. with 1-butanamine and methanimidamide (1:4:2:2)
Ruddlesden-Popper	Plumbate(4-), di-μ-iodooctaiodotri-, hydrogen, compd. with benzenemethanamine and methanamine (1:4:2:2)
Ruddlesden-Popper	Plumbate(4-), di-μ-iodooctaiodotri-, hydrogen, compd. with methanamine and 2-methyl-1-propanamine (1:4:2:2)

Ruddlesden-Popper	Plumbate(4-), hexa-μ-bromotetrabromotri-, hydrogen, compd. with benzeneethanamine and methanimidamide (1:4:2:2)
Ruddlesden-Popper	Plumbate(4-), hexa-μ-bromotetrabromotri-, hydrogen, compd. with ethanamine (1:4:4)
Ruddlesden-Popper	Plumbate(4-), hexa-μ-iodotetraiodotri-, hydrogen, compd. with 1-butanamine and methanamine (1:4:2:2)
Ruddlesden-Popper	Plumbate(4-), hexa-μ-iodotetraiodotri-, hydrogen, compd. with 1-hexanamine and methanamine (1:4:2:2)
Ruddlesden-Popper	Plumbate(4-), hexaiodo-, (OC-6-11)-, hydrogen, compd. with benzeneethanamine (1:4:4)
Ruddlesden-Popper	Plumbate(4-), hexaiodo-, tetracesium, (OC-6-11)-
Ruddlesden-Popper	Plumbate(4-), tri-μ-iododi-μ3-iodopentaiodotri-, hydrogen, compd. with 1-butanamine and methanamine (1:4:2:2)
Ruddlesden-Popper	Plumbate(4-), tri-μ-iodoheptaiodotri-, hydrogen, compd. with benzeneethanamine and methanamine (1:4:2:2)
Ruddlesden-Popper	Plumbate(5-), tri-μ-bromodecabromotetra-, cesium hydrogen, compd. with benzeneethanamine (1:3:2:2)
Ruddlesden-Popper	Plumbate(5-), tri-μ-bromodecabromotetra-, hydrogen, compd. with 1-butanamine and methanamine (1:5:2:3)
Ruddlesden-Popper	Plumbate(5-), tri-μ-bromodecabromotetra-, hydrogen, compd. with benzeneethanamine and 2-propanamine (1:5:2:3)
Ruddlesden-Popper	Plumbate(5-), tri-μ-iododecaiodotetra-, hydrogen, compd. with 1-butanamine and methanamine (1:5:2:3)
Ruddlesden-Popper	Plumbate(5-), tri-μ-iododecaiodotetra-, hydrogen, compd. with benzeneethanamine and methanamine (1:5:2:3)
Ruddlesden-Popper	Plumbate(5-), tri-μ-iododecaiodotetra-, hydrogen, compd. with methanamine and 2-methyl-1-propanamine (1:5:3:2)
Ruddlesden-Popper	Plumbate(6-), tetra-μ-iodododecaiodopenta-, hydrogen, compd. with 1-butanamine and methanamine (1:6:2:4)
Ruddlesden-Popper	Plumbate(6-), tetra-μ-iodododecaiodopenta-, hydrogen, compd. with 4-fluorobenzeneethanamine and methanamine (1:6:2:4)
Ruddlesden-Popper	Plumbate(6-), tetra-μ-iodododecaiodopenta-, hydrogen, compd. with benzeneethanamine and methanamine (1:6:2:4)

Ruddlesden-Popper	Plumbate(6-), tetra-μ-iodododecaiodopenta-, hydrogen, compd. with methanamine and 1-octanamine (1:6:4:2)
Ruddlesden-Popper	Plumbate(6-), tetra-μ-iodododecaiodopenta-, hydrogen, compd. with methanamine and 1-propanamine (1:6:4:2)
Ruddlesden-Popper	Plumbate(6-), tetra-μ-iodododecaiodopenta-, hydrogen, compd. with methanamine and 2-methyl-1-propanamine (1:6:4:2)
Ruddlesden-Popper	Stannate(2-), tetrabromo-, (T-4)-, dihydrogen, compd. with benzeneethanamine (1:2)
Ruddlesden-Popper	Stannate(2-), tetrabromo-, (T-4)-, hydrogen, compd. with 1-butanamine (1:2:2)
Ruddlesden-Popper	Stannate(2-), tetrabromo-, (T-4)-, hydrogen, compd. with 1-dodecanamine (1:2:2)
Ruddlesden-Popper	Stannate(2-), tetrabromo-, (T-4)-, hydrogen, compd. with 1-octadecanamine (1:2:2)
Ruddlesden-Popper	Stannate(2-), tetrabromo-, (T-4)-, hydrogen, compd. with 1-octanamine (1:2:2)
Ruddlesden-Popper	Stannate(2-), tetrachloro-, (T-4)-, dihydrogen, compd. with 1-butanamine (1:2)
Ruddlesden-Popper	Stannate(2-), tetraiodo-, (T-4)-, dihydrogen, compd. with 4-fluorobenzeneethanamine (1:2)
Ruddlesden-Popper	Stannate(2-), tetraiodo-, (T-4)-, hydrogen, compd. with 1-butanamine (1:2:2)
Ruddlesden-Popper	Stannate(2-), tetraiodo-, (T-4)-, hydrogen, compd. with 1-hexanamine (1:2:2)
Ruddlesden-Popper	Stannate(2-), tetraiodo-, (T-4)-, hydrogen, compd. with 1-octanamine (1:2:2)
Ruddlesden-Popper	Stannate(2-), tetraiodo-, (T-4)-, hydrogen, compd. with 2-thiopheneethanamine (1:2:2)
Ruddlesden-Popper	β-Alanine, (T-4)-tetraiodoplumbate(2-) (2:1)
**Table S8C: Perovskite grouped by number of layers**	
**Perovskite layer number group**	**Substance Name**
1 layer	Benzeneethanamine, (T-4)-tetraiodostannate(2-) (2:1)
1 layer	Plumbate(2-), bis(1-butanamine)tetraiodo-, hydrogen (1:2)
1 layer	Plumbate(2-), bromotrichloro-, (T-4)-, dihydrogen, compd. with benzeneethanamine (1:2)
1 layer	Plumbate(2-), bromotrichloro-, (T-4)-, hydrogen, compd. with benzenemethanamine (1:2:2)
1 layer	Plumbate(2-), bromotriiodo-, (T-4)-, dihydrogen, compd. with benzeneethanamine (1:2)

1 layer	Plumbate(2-), chlorotriiodo-, (T-4)-, hydrogen, compd. with methanamine (1:2:2)
1 layer	Plumbate(2-), dibromodichloro-, (T-4)-, dihydrogen, compd. with benzeneethanamine (1:2)
1 layer	Plumbate(2-), dibromodichloro-, (T-4)-, hydrogen, compd. with benzenemethanamine (1:2:2)
1 layer	Plumbate(2-), dibromodiiodo-, (T-4)-, dihydrogen, compd. with benzeneethanamine (1:2)
1 layer	Plumbate(2-), tetrabromo-, (T-4)-, dihydrogen, compd. with 1H-imidazole-1-propanamine (1:1)
1 layer	Plumbate(2-), tetrabromo-, (T-4)-, dihydrogen, compd. with 2-propyn-1-amine (1:2)
1 layer	Plumbate(2-), tetrabromo-, (T-4)-, hydrogen, compd. with 1-butanamine (1:2:2)
1 layer	Plumbate(2-), tetrabromo-, (T-4)-, hydrogen, compd. with 1-cyclohexene-1-ethanamine (1:2:2)
1 layer	Plumbate(2-), tetrabromo-, (T-4)-, hydrogen, compd. with 1-hexanamine (1:2:2)
1 layer	Plumbate(2-), tetrabromo-, (T-4)-, hydrogen, compd. with 1-octanamine (1:2:2)
1 layer	Plumbate(2-), tetrabromo-, (T-4)-, hydrogen, compd. with 1,4-butanediamine (1:2:2)
1 layer	Plumbate(2-), tetrabromo-, (T-4)-, hydrogen, compd. with 2-naphthaleneethanamine (1:2:2)
1 layer	Plumbate(2-), tetrabromo-, (T-4)-, hydrogen, compd. with 2-naphthalenemethanamine (1:2:2)
1 layer	Plumbate(2-), tetrabromo-, (T-4)-, hydrogen, compd. with 3,5-decadiyn-1-amine (1:2:2)
1 layer	Plumbate(2-), tetrabromo-, (T-4)-, hydrogen, compd. with 4-fluorobenzeneethanamine (1:2:2)
1 layer	Plumbate(2-), tetrabromo-, (T-4)-, hydrogen, compd. with (αR)-4-fluoro-α-methylbenzenemethanamine (1:2:2)
1 layer	Plumbate(2-), tetrabromo-, (T-4)-, hydrogen, compd. with benzeneethanamine (1:2:2)
1 layer	Plumbate(2-), tetrabromo-, (T-4)-, hydrogen, compd. with benzenemethanamine (1:2:2)
1 layer	Plumbate(2-), tetrabromo-, (T-4)-, hydrogen, compd. with cyclohexanamine (1:2:2)
1 layer	Plumbate(2-), tetrabromo-, (T-4)-, hydrogen, compd. with ethanamine (1:2:2)

1 layer	Plumbate(2-), tetrabromo-, (T-4)-, hydrogen, compd. with methylhydrazine (1:2:2)
1 layer	Plumbate(2-), tetrabromo-, (T-4)-, hydrogen, compd. with tetrahydro-2H-pyran-4-amine (1:2:2)
1 layer	Plumbate(2-), tetrachloro-, (T-4)-, dihydrogen, compd. with benzenemethanamine (1:2)
1 layer	Plumbate(2-), tetrachloro-, (T-4)-, hydrogen, compd. with 1-butanamine (1:2:2)
1 layer	Plumbate(2-), tetrachloro-, (T-4)-, hydrogen, compd. with 2-naphthaleneethanamine (1:2:2)
1 layer	Plumbate(2-), tetrachloro-, (T-4)-, hydrogen, compd. with 2-naphthalenemethanamine (1:2:2)
1 layer	Plumbate(2-), tetrachloro-, (T-4)-, hydrogen, compd. with 4-fluorobenzeneethanamine (1:2:2)
1 layer	Plumbate(2-), tetrachloro-, (T-4)-, hydrogen, compd. with benzeneethanamine (1:2:2)
1 layer	Plumbate(2-), tetraiodo-, (T-4)-, dihydrogen, compd. with 2-aminoethanol (1:2)
1 layer	Plumbate(2-), tetraiodo-, (T-4)-, dihydrogen, compd. with 2,2′-bi-1H-imidazole (1:1)
1 layer	Plumbate(2-), tetraiodo-, (T-4)-, dihydrogen, compd. with 4-fluorobenzenemethanamine (1:2)
1 layer	Plumbate(2-), tetraiodo-, (T-4)-, dihydrogen, compd. with 4-methoxybenzeneethanamine (1:2)
1 layer	Plumbate(2-), tetraiodo-, (T-4)-, dihydrogen, compd. with benzenamine (1:2)
1 layer	Plumbate(2-), tetraiodo-, (T-4)-, dihydrogen, compd. with benzenebutanamine (1:2)
1 layer	Plumbate(2-), tetraiodo-, (T-4)-, dihydrogen, compd. with guanidine (1:2)
1 layer	Plumbate(2-), tetraiodo-, (T-4)-, dihydrogen, compd. with methanamine (1:2)
1 layer	Plumbate(2-), tetraiodo-, (T-4)-, dihydrogen, compd. with α-methylbenzenemethanamine (1:2)
1 layer	Plumbate(2-), tetraiodo-, (T-4)-, hydrogen, compd. with 1-butanamine (1:2:2)
1 layer	Plumbate(2-), tetraiodo-, (T-4)-, hydrogen, compd. with 1-cyclohexene-1-ethanamine (1:2:2)
1 layer	Plumbate(2-), tetraiodo-, (T-4)-, hydrogen, compd. with 1-decanamine (1:2:2)
1 layer	Plumbate(2-), tetraiodo-, (T-4)-, hydrogen, compd. with 1-dodecanamine (1:2:2)

1 layer	Plumbate(2-), tetraiodo-, (T-4)-, hydrogen, compd. with 1-heptanamine (1:2:2)
1 layer	Plumbate(2-), tetraiodo-, (T-4)-, hydrogen, compd. with 1-hexadecanamine (1:2:2)
1 layer	Plumbate(2-), tetraiodo-, (T-4)-, hydrogen, compd. with 1-hexanamine (1:2:2)
1 layer	Plumbate(2-), tetraiodo-, (T-4)-, hydrogen, compd. with 1-naphthalenemethanamine (1:2:2)
1 layer	Plumbate(2-), tetraiodo-, (T-4)-, hydrogen, compd. with 1-nonanamine (1:2:2)
1 layer	Plumbate(2-), tetraiodo-, (T-4)-, hydrogen, compd. with 1-octanamine (1:2:2)
1 layer	Plumbate(2-), tetraiodo-, (T-4)-, hydrogen, compd. with 1-pentanamine (1:2:2)
1 layer	Plumbate(2-), tetraiodo-, (T-4)-, hydrogen, compd. with 1-propanamine (1:2:2)
1 layer	Plumbate(2-), tetraiodo-, (T-4)-, hydrogen, compd. with 1-pyrenebutanamine (1:2:2)
1 layer	Plumbate(2-), tetraiodo-, (T-4)-, hydrogen, compd. with 1-tetradecanamine (1:2:2)
1 layer	Plumbate(2-), tetraiodo-, (T-4)-, hydrogen, compd. with 1-undecanamine (1:2:2)
1 layer	Plumbate(2-), tetraiodo-, (T-4)-, hydrogen, compd. with 1,3,5-hexatriene-1,6-diamine (1:2:1)
1 layer	Plumbate(2-), tetraiodo-, (T-4)-, hydrogen, compd. with 1,4-benzenediamine (1:2:1)
1 layer	Plumbate(2-), tetraiodo-, (T-4)-, hydrogen, compd. with 1,4-benzenedimethanamine (1:2:1)
1 layer	Plumbate(2-), tetraiodo-, (T-4)-, hydrogen, compd. with 1,4-butanediamine (1:2:1)
1 layer	Plumbate(2-), tetraiodo-, (T-4)-, hydrogen, compd. with 1,5-naphthalenediamine (1:2:1)
1 layer	Plumbate(2-), tetraiodo-, (T-4)-, hydrogen, compd. with 1,6-hexanediamine (1:2:1)
1 layer	Plumbate(2-), tetraiodo-, (T-4)-, hydrogen, compd. with 1,7-heptanediamine (1:2:1)
1 layer	Plumbate(2-), tetraiodo-, (T-4)-, hydrogen, compd. with 1,8-octanediamine (1:2:1)
1 layer	Plumbate(2-), tetraiodo-, (T-4)-, hydrogen, compd. with 1H-imidazole-5-ethanamine (1:2:1)
1 layer	Plumbate(2-), tetraiodo-, (T-4)-, hydrogen, compd. with 2-fluorobenzeneethanamine (1:2:2)
1 layer	Plumbate(2-), tetraiodo-, (T-4)-, hydrogen, compd. with 2-methyl-1-propanamine (1:2:2)

1 layer	Plumbate(2-), tetraiodo-, (T-4)-, hydrogen, compd. with 2-naphthaleneethanamine (1:2:2)
1 layer	Plumbate(2-), tetraiodo-, (T-4)-, hydrogen, compd. with 2-naphthalenemethanamine (1:2:2)
1 layer	Plumbate(2-), tetraiodo-, (T-4)-, hydrogen, compd. with 2-pyridineethanamine (1:2:1)
1 layer	Plumbate(2-), tetraiodo-, (T-4)-, hydrogen, compd. with 2-thiopheneethanamine (1:2:2)
1 layer	Plumbate(2-), tetraiodo-, (T-4)-, hydrogen, compd. with 2,2′-(2,5-cyclohexadiene-1,4-diylidene)bis[propanedinitrile] and 1-pyrenebutanamine (1:2:2:2)
1 layer	Plumbate(2-), tetraiodo-, (T-4)-, hydrogen, compd. with 2,2′-[1,2-ethanediylbis(oxy)]bis[ethanamine] (1:2:1)
1 layer	Plumbate(2-), tetraiodo-, (T-4)-, hydrogen, compd. with 2,3,4,5,6-pentafluorobenzeneethanamine (1:2:2)
1 layer	Plumbate(2-), tetraiodo-, (T-4)-, hydrogen, compd. with 3-fluorobenzeneethanamine (1:2:2)
1 layer	Plumbate(2-), tetraiodo-, (T-4)-, hydrogen, compd. with 3-methyl-1-butanamine (1:2:2)
1 layer	Plumbate(2-), tetraiodo-, (T-4)-, hydrogen, compd. with 3-piperidinemethanamine (1:2:1)
1 layer	Plumbate(2-), tetraiodo-, (T-4)-, hydrogen, compd. with 3-pyridineethanamine (1:2:1)
1 layer	Plumbate(2-), tetraiodo-, (T-4)-, hydrogen, compd. with 3-pyridinemethanamine (1:2:1)
1 layer	Plumbate(2-), tetraiodo-, (T-4)-, hydrogen, compd. with 4-(trifluoromethyl)benzeneethanamine (1:2:2)
1 layer	Plumbate(2-), tetraiodo-, (T-4)-, hydrogen, compd. with 4-bromo-α-methylbenzenemethanamine (1:2:2)
1 layer	Plumbate(2-), tetraiodo-, (T-4)-, hydrogen, compd. with 4-fluorobenzeneethanamine (1:2:1)
1 layer	Plumbate(2-), tetraiodo-, (T-4)-, hydrogen, compd. with 4-fluorobenzeneethanamine (1:2:2)
1 layer	Plumbate(2-), tetraiodo-, (T-4)-, hydrogen, compd. with 4-piperidinemethanamine (1:2:1)
1 layer	Plumbate(2-), tetraiodo-, (T-4)-, hydrogen, compd. with 4-pyridineethanamine (1:2:1)
1 layer	Plumbate(2-), tetraiodo-, (T-4)-, hydrogen, compd. with 4-pyridinemethanamine (1:2:1)

1 layer	Plumbate(2-), tetraiodo-, (T-4)-, hydrogen, compd. with 4,4-difluoropiperidine (1:2:2)
1 layer	Plumbate(2-), tetraiodo-, (T-4)-, hydrogen, compd. with (αR)-α-methylbenzenemethanamine (1:2:2)
1 layer	Plumbate(2-), tetraiodo-, (T-4)-, hydrogen, compd. with (αS)-α-methylbenzenemethanamine (1:2:2)
1 layer	Plumbate(2-), tetraiodo-, (T-4)-, hydrogen, compd. with benzeneethanamine (1:2:2)
1 layer	Plumbate(2-), tetraiodo-, (T-4)-, hydrogen, compd. with benzenemethanamine (1:2:2)
1 layer	Plumbate(2-), tetraiodo-, (T-4)-, hydrogen, compd. with cyclohexanamine (1:2:2)
1 layer	Plumbate(2-), tetraiodo-, (T-4)-, hydrogen, compd. with cyclohexaneethanamine (1:2:2)
1 layer	Plumbate(2-), tetraiodo-, (T-4)-, hydrogen, compd. with cyclohexanemethanamine (1:2:2)
1 layer	Plumbate(2-), tetraiodo-, (T-4)-, hydrogen, compd. with cyclopentanamine (1:2:2)
1 layer	Plumbate(2-), tetraiodo-, (T-4)-, hydrogen, compd. with ethanamine (1:2:2)
1 layer	Plumbate(2-), tetraiodo-, (T-4)-, hydrogen, compd. with guanidine and methanimidamide (1:2:1:1)
1 layer	Plumbate(2-), tetraiodo-, (T-4)-, hydrogen, compd. with β-methylbenzeneethanamine (1:2:2)
1 layer	Stannate(2-), tetrabromo-, (T-4)-, dihydrogen, compd. with benzeneethanamine (1:2)
1 layer	Stannate(2-), tetrabromo-, (T-4)-, hydrogen, compd. with 1-butanamine (1:2:2)
1 layer	Stannate(2-), tetrabromo-, (T-4)-, hydrogen, compd. with 1-dodecanamine (1:2:2)
1 layer	Stannate(2-), tetrabromo-, (T-4)-, hydrogen, compd. with 1-octadecanamine (1:2:2)
1 layer	Stannate(2-), tetrabromo-, (T-4)-, hydrogen, compd. with 1-octanamine (1:2:2)
1 layer	Stannate(2-), tetrabromo-, (T-4)-, hydrogen, compd. with 1,8-octanediamine (1:2:1)
1 layer	Stannate(2-), tetrachloro-, (T-4)-, dihydrogen, compd. with 1-butanamine (1:2)
1 layer	Stannate(2-), tetraiodo-, (T-4)-, dihydrogen, compd. with 1,4-butanediamine (1:1)
1 layer	Stannate(2-), tetraiodo-, (T-4)-, dihydrogen, compd. with 4-fluorobenzeneethanamine (1:2)
1 layer	Stannate(2-), tetraiodo-, (T-4)-, hydrogen, compd. with 1-butanamine (1:2:2)

1 layer	Stannate(2-), tetraiodo-, (T-4)-, hydrogen, compd. with 1-hexanamine (1:2:2)
1 layer	Stannate(2-), tetraiodo-, (T-4)-, hydrogen, compd. with 1-octanamine (1:2:2)
1 layer	Stannate(2-), tetraiodo-, (T-4)-, hydrogen, compd. with 2-thiopheneethanamine (1:2:2)
1 layer	β-Alanine, (T-4)-tetraiodoplumbate(2-) (2:1)
2 layers	Cesium lead bromide iodide (CsPb2BrI4)
2 layers	Cesium lead iodide (CsPb2I7)
2 layers	Plumbate(3-), μ-bromohexabromodi-, hydrogen, compd. with 1-butanamine and methanamine (1:3:2:1)
2 layers	Plumbate(3-), μ-bromohexabromodi-, hydrogen, compd. with 1-butanamine and methanimidamide (1:3:2:1)
2 layers	Plumbate(3-), μ-bromohexabromodi-, hydrogen, compd. with 1-heptanamine and methanamine (1:3:2:1)
2 layers	Plumbate(3-), μ-bromohexabromodi-, hydrogen, compd. with 1-hexanamine and methanamine (1:3:2:1)
2 layers	Plumbate(3-), μ-bromohexabromodi-, hydrogen, compd. with methanamine and 1-octanamine (1:3:1:2)
2 layers	Plumbate(3-), μ-bromohexabromodi-, hydrogen, compd. with methanimidamide and 1-naphthalenemethanamine (1:3:1:2)
2 layers	Plumbate(3-), μ-bromohexabromodi-, trihydrogen, compd. with benzeneethanamine and methanamine (1:2:1)
2 layers	Plumbate(3-), μ-iodohexaiododi-, hydrogen, compd. with 1-butanamine and methanamine (1:3:2:1)
2 layers	Plumbate(3-), μ-iodohexaiododi-, hydrogen, compd. with 1-butanamine and methanimidamide (1:3:2:1)
2 layers	Plumbate(3-), μ-iodohexaiododi-, hydrogen, compd. with 1-dodecanamine and methanamine (1:3:2:1)
2 layers	Plumbate(3-), μ-iodohexaiododi-, hydrogen, compd. with 1-hexanamine and methanamine (1:3:2:1)
2 layers	Plumbate(3-), μ-iodohexaiododi-, hydrogen, compd. with 1,4-benzenedimethanamine and methanimidamide (1:3:1:1)
2 layers	Plumbate(3-), μ-iodohexaiododi-, hydrogen, compd. with benzeneethanamine and methanamine (1:3:2:1)

2 layers	Plumbate(3-), μ-iodohexaiododi-, hydrogen, compd. with benzenemethanamine and methanamine (1:3:2:1)
2 layers	Plumbate(3-), μ-iodohexaiododi-, hydrogen, compd. with methanamine and 1-octanamine (1:3:1:2)
2 layers	Plumbate(3-), μ-iodohexaiododi-, hydrogen, compd. with methanamine and 1-tetradecanamine (1:3:1:2)
2 layers	Plumbate(3-), μ-iodohexaiododi-, hydrogen, compd. with methanamine and 1,3-propanediamine (1:3:1:1)
2 layers	Plumbate(3-), μ-iodohexaiododi-, hydrogen, compd. with methanamine and 1,8-octanediamine (1:3:1:1)
2 layers	Plumbate(3-), μ-iodohexaiododi-, hydrogen, compd. with methanamine and 2-methyl-1-propanamine (1:3:1:2)
2 layers	Plumbate(3-), μ-iodohexaiododi-, hydrogen, compd. with methanimidamide and 1-naphthalenemethanamine (1:3:1:2)
2 layers	Plumbate(3-), μ-iodohexaiododi-, hydrogen, compd. with N-methylmethanamine and 1-pentanamine (1:3:1:2)
2 layers	Plumbate(3-), μ-iodohexaiododi-, trihydrogen, compd. with methanamine and 2-thiophenemethanamine (1:1:2)
3 layers	Plumbate(4-), di-μ-bromooctabromotri-, cesium hydrogen, compd. with benzeneethanamine (1:2:2:2)
3 layers	Plumbate(4-), di-μ-bromooctabromotri-, hydrogen, compd. with 1-butanamine, compd. with methanamine (1:4:2:2)
3 layers	Plumbate(4-), di-μ-bromooctabromotri-, hydrogen, compd. with benzeneethanamine and methanamine (1:4:2:2)
3 layers	Plumbate(4-), di-μ-iodooctaiodotri-, hydrogen, compd. with 1-butanamine and ethanamine (1:4:2:2)
3 layers	Plumbate(4-), di-μ-iodooctaiodotri-, hydrogen, compd. with 1-butanamine and methanamine (1:4:2:2)
3 layers	Plumbate(4-), di-μ-iodooctaiodotri-, hydrogen, compd. with 1-butanamine and methanimidamide (1:4:2:2)
3 layers	Plumbate(4-), di-μ-iodooctaiodotri-, hydrogen, compd. with benzenemethanamine and methanamine (1:4:2:2)

3 layers	Plumbate(4-), di-μ-iodooctaiodotri-, hydrogen, compd. with methanamine and 1,3-propanediamine (1:4:2:1)
3 layers	Plumbate(4-), di-μ-iodooctaiodotri-, hydrogen, compd. with methanamine and 1,8-octanediamine (1:4:2:1)
3 layers	Plumbate(4-), di-μ-iodooctaiodotri-, hydrogen, compd. with methanamine and 2-methyl-1-propanamine (1:4:2:2)
3 layers	Plumbate(4-), di-μ-iodooctaiodotri-, hydrogen, compd. with methanamine and 4-piperidinemethanamine (1:4:2:1)
3 layers	Plumbate(4-), hexa-μ-bromotetrabromotri-, hydrogen, compd. with benzeneethanamine and methanimidamide (1:4:2:2)
3 layers	Plumbate(4-), hexa-μ-bromotetrabromotri-, hydrogen, compd. with ethanamine (1:4:4)
3 layers	Plumbate(4-), hexa-μ-iodotetraiodotri-, hydrogen, compd. with 1-butanamine and methanamine (1:4:2:2)
3 layers	Plumbate(4-), hexa-μ-iodotetraiodotri-, hydrogen, compd. with 1-hexanamine and methanamine (1:4:2:2)
3 layers	Plumbate(4-), hexa-μ-iodotetraiodotri-, hydrogen, compd. with 1,4-benzenedimethanamine and methanimidamide (1:4:1:2)
3 layers	Plumbate(4-), hexa-μ-iodotetraiodotri-, hydrogen, compd. with 1,4-butanediamine and methanamine (2:8:1:6)
3 layers	Plumbate(4-), hexaiodo-, (OC-6-11)-, hydrogen, compd. with benzeneethanamine (1:4:4)
3 layers	Plumbate(4-), tri-μ-iododi-μ3-iodopentaiodotri-, hydrogen, compd. with 1-butanamine and methanamine (1:4:2:2)
3 layers	Plumbate(4-), tri-μ-iodoheptaiodotri-, hydrogen, compd. with benzeneethanamine and methanamine (1:4:2:2)
4 layers	Plumbate(5-), tri-μ-bromodecabromotetra-, cesium hydrogen, compd. with benzeneethanamine (1:3:2:2)
4 layers	Plumbate(5-), tri-μ-bromodecabromotetra-, hydrogen, compd. with 1-butanamine and methanamine (1:5:2:3)
4 layers	Plumbate(5-), tri-μ-bromodecabromotetra-, hydrogen, compd. with benzeneethanamine and 2-propanamine (1:5:2:3)

4 layers	Plumbate(5-), tri-μ-iododecaiodotetra-, hydrogen, compd. with 1-butanamine and methanamine (1:5:2:3)
4 layers	Plumbate(5-), tri-μ-iododecaiodotetra-, hydrogen, compd. with 1,4-benzenedimethanamine and methanimidamide (1:5:1:3)
4 layers	Plumbate(5-), tri-μ-iododecaiodotetra-, hydrogen, compd. with benzeneethanamine and methanamine (1:5:2:3)
4 layers	Plumbate(5-), tri-μ-iododecaiodotetra-, hydrogen, compd. with methanamine and 1,3-propanediamine (1:5:3:1)
4 layers	Plumbate(5-), tri-μ-iododecaiodotetra-, hydrogen, compd. with methanamine and 1,8-octanediamine (1:5:3:1)
4 layers	Plumbate(5-), tri-μ-iododecaiodotetra-, hydrogen, compd. with methanamine and 2-methyl-1-propanamine (1:5:3:2)
4 layers	Plumbate(5-), tri-μ-iododecaiodotetra-, hydrogen, compd. with methanamine and 3-pyridinemethanamine (1:5:3:1)
4 layers	Plumbate(5-), tri-μ-iododecaiodotetra-, hydrogen, compd. with methanamine and 4-pyridinemethanamine (1:5:3:1)
4 layers	Plumbate(5-), μ-bromooctabromodi-, hydrogen, compd. with 1,2-ethanediamine hydrobromide (1:5:4:3)
5 layers	Plumbate(4-), hexaiodo-, tetracesium, (OC-6-11)-
5 layers	Plumbate(6-), tetra-μ-iodododecaiodopenta-, hydrogen, compd. with 1-butanamine and methanamine (1:6:2:4)
5 layers	Plumbate(6-), tetra-μ-iodododecaiodopenta-, hydrogen, compd. with 4-fluorobenzeneethanamine and methanamine (1:6:2:4)
5 layers	Plumbate(6-), tetra-μ-iodododecaiodopenta-, hydrogen, compd. with benzeneethanamine and methanamine (1:6:2:4)
5 layers	Plumbate(6-), tetra-μ-iodododecaiodopenta-, hydrogen, compd. with methanamine and 1-octanamine (1:6:4:2)
5 layers	Plumbate(6-), tetra-μ-iodododecaiodopenta-, hydrogen, compd. with methanamine and 1-propanamine (1:6:4:2)
5 layers	Plumbate(6-), tetra-μ-iodododecaiodopenta-, hydrogen, compd. with methanamine and 2-methyl-1-propanamine (1:6:4:2)
**Table S8D: Perovskites grouped by halides**	

Perovskite halide group	Substance Name
Bromide	Barium cesium lead bromide (Ba2CsPb2Br9)
Bromide	Plumbate(2-), tetrabromo-, (T-4)-, dihydrogen, compd. with 1H-imidazole-1-propanamine (1:1)
Bromide	Plumbate(2-), tetrabromo-, (T-4)-, dihydrogen, compd. with 2-propyn-1-amine (1:2)
Bromide	Plumbate(2-), tetrabromo-, (T-4)-, hydrogen, compd. with 1-butanamine (1:2:2)
Bromide	Plumbate(2-), tetrabromo-, (T-4)-, hydrogen, compd. with 1-cyclohexene-1-ethanamine (1:2:2)
Bromide	Plumbate(2-), tetrabromo-, (T-4)-, hydrogen, compd. with 1-hexanamine (1:2:2)
Bromide	Plumbate(2-), tetrabromo-, (T-4)-, hydrogen, compd. with 1-octanamine (1:2:2)
Bromide	Plumbate(2-), tetrabromo-, (T-4)-, hydrogen, compd. with 1,4-butanediamine (1:2:2)
Bromide	Plumbate(2-), tetrabromo-, (T-4)-, hydrogen, compd. with 2-naphthaleneethanamine (1:2:2)
Bromide	Plumbate(2-), tetrabromo-, (T-4)-, hydrogen, compd. with 2-naphthalenemethanamine (1:2:2)
Bromide	Plumbate(2-), tetrabromo-, (T-4)-, hydrogen, compd. with 3,5-decadiyn-1-amine (1:2:2)
Bromide	Plumbate(2-), tetrabromo-, (T-4)-, hydrogen, compd. with 4-fluorobenzeneethanamine (1:2:2)
Bromide	Plumbate(2-), tetrabromo-, (T-4)-, hydrogen, compd. with (αR)-4-fluoro-α-methylbenzenemethanamine (1:2:2)
Bromide	Plumbate(2-), tetrabromo-, (T-4)-, hydrogen, compd. with benzeneethanamine (1:2:2)
Bromide	Plumbate(2-), tetrabromo-, (T-4)-, hydrogen, compd. with benzenemethanamine (1:2:2)
Bromide	Plumbate(2-), tetrabromo-, (T-4)-, hydrogen, compd. with cyclohexanamine (1:2:2)
Bromide	Plumbate(2-), tetrabromo-, (T-4)-, hydrogen, compd. with ethanamine (1:2:2)
Bromide	Plumbate(2-), tetrabromo-, (T-4)-, hydrogen, compd. with methylhydrazine (1:2:2)
Bromide	Plumbate(2-), tetrabromo-, (T-4)-, hydrogen, compd. with tetrahydro-2H-pyran-4-amine (1:2:2)
Bromide	Plumbate(3-), µ-bromohexabromodi-, hydrogen, compd. with 1-butanamine and methanamine (1:3:2:1)

Bromide	Plumbate(3-), μ-bromohexabromodi-, hydrogen, compd. with 1-butanamine and methanimidamide (1:3:2:1)
Bromide	Plumbate(3-), μ-bromohexabromodi-, hydrogen, compd. with 1-heptanamine and methanamine (1:3:2:1)
Bromide	Plumbate(3-), μ-bromohexabromodi-, hydrogen, compd. with 1-hexanamine and methanamine (1:3:2:1)
Bromide	Plumbate(3-), μ-bromohexabromodi-, hydrogen, compd. with methanamine and 1-octanamine (1:3:1:2)
Bromide	Plumbate(3-), μ-bromohexabromodi-, hydrogen, compd. with methanimidamide and 1-naphthalenemethanamine (1:3:1:2)
Bromide	Plumbate(3-), μ-bromohexabromodi-, trihydrogen, compd. with benzeneethanamine and methanamine (1:2:1)
Bromide	Plumbate(4-), di-μ-bromooctabromotri-, cesium hydrogen, compd. with benzeneethanamine (1:2:2:2)
Bromide	Plumbate(4-), di-μ-bromooctabromotri-, hydrogen, compd. with 1-butanamine, compd. with methanamine (1:4:2:2)
Bromide	Plumbate(4-), di-μ-bromooctabromotri-, hydrogen, compd. with benzeneethanamine and methanamine (1:4:2:2)
Bromide	Plumbate(4-), hexa-μ-bromotetrabromotri-, hydrogen, compd. with benzeneethanamine and methanimidamide (1:4:2:2)
Bromide	Plumbate(4-), hexa-μ-bromotetrabromotri-, hydrogen, compd. with ethanamine (1:4:4)
Bromide	Plumbate(5-), tri-μ-bromodecabromotetra-, cesium hydrogen, compd. with benzeneethanamine (1:3:2:2)
Bromide	Plumbate(5-), tri-μ-bromodecabromotetra-, hydrogen, compd. with 1-butanamine and methanamine (1:5:2:3)
Bromide	Plumbate(5-), tri-μ-bromodecabromotetra-, hydrogen, compd. with benzeneethanamine and 2-propanamine (1:5:2:3)
Bromide	Plumbate(5-), μ-bromooctabromodi-, hydrogen, compd. with 1,2-ethanediamine hydrobromide (1:5:4:3)
Bromide	Stannate(2-), tetrabromo-, (T-4)-, dihydrogen, compd. with benzeneethanamine (1:2)
Bromide	Stannate(2-), tetrabromo-, (T-4)-, hydrogen, compd. with 1-butanamine (1:2:2)
Bromide	Stannate(2-), tetrabromo-, (T-4)-, hydrogen, compd. with 1-dodecanamine (1:2:2)

Bromide	Stannate(2-), tetrabromo-, (T-4)-, hydrogen, compd. with 1-octadecanamine (1:2:2)
Bromide	Stannate(2-), tetrabromo-, (T-4)-, hydrogen, compd. with 1-octanamine (1:2:2)
Bromide	Stannate(2-), tetrabromo-, (T-4)-, hydrogen, compd. with 1,8-octanediamine (1:2:1)
Chloride	Barium cesium lead chloride (Ba2CsPb2Cl9)
Chloride	Plumbate(2-), tetrachloro-, (T-4)-, dihydrogen, compd. with benzenemethanamine (1:2)
Chloride	Plumbate(2-), tetrachloro-, (T-4)-, hydrogen, compd. with 1-butanamine (1:2:2)
Chloride	Plumbate(2-), tetrachloro-, (T-4)-, hydrogen, compd. with 2-naphthaleneethanamine (1:2:2)
Chloride	Plumbate(2-), tetrachloro-, (T-4)-, hydrogen, compd. with 2-naphthalenemethanamine (1:2:2)
Chloride	Plumbate(2-), tetrachloro-, (T-4)-, hydrogen, compd. with 4-fluorobenzeneethanamine (1:2:2)
Chloride	Plumbate(2-), tetrachloro-, (T-4)-, hydrogen, compd. with benzeneethanamine (1:2:2)
Chloride	Stannate(2-), tetrachloro-, (T-4)-, dihydrogen, compd. with 1-butanamine (1:2)
Iodide	Barium cesium lead iodide (Ba2CsPb2I9)
Iodide	Barium lead iodide (Ba2PbI6)
Iodide	Benzeneethanamine, (T-4)-tetraiodostannate(2-) (2:1)
Iodide	Cesium lead iodide (CsPb2I7)
Iodide	Plumbate(2-), bis(1-butanamine)tetraiodo-, hydrogen (1:2)
Iodide	Plumbate(2-), tetraiodo-, (T-4)-, dihydrogen, compd. with 2-aminoethanol (1:2)
Iodide	Plumbate(2-), tetraiodo-, (T-4)-, dihydrogen, compd. with 2,2′-bi-1H-imidazole (1:1)
Iodide	Plumbate(2-), tetraiodo-, (T-4)-, dihydrogen, compd. with 4-fluorobenzenemethanamine (1:2)
Iodide	Plumbate(2-), tetraiodo-, (T-4)-, dihydrogen, compd. with 4-methoxybenzeneethanamine (1:2)
Iodide	Plumbate(2-), tetraiodo-, (T-4)-, dihydrogen, compd. with benzenamine (1:2)
Iodide	Plumbate(2-), tetraiodo-, (T-4)-, dihydrogen, compd. with benzenebutanamine (1:2)
Iodide	Plumbate(2-), tetraiodo-, (T-4)-, dihydrogen, compd. with guanidine (1:2)
Iodide	Plumbate(2-), tetraiodo-, (T-4)-, dihydrogen, compd. with methanamine (1:2)

Iodide	Plumbate(2-), tetraiodo-, (T-4)-, dihydrogen, compd. with α-methylbenzenemethanamine (1:2)
Iodide	Plumbate(2-), tetraiodo-, (T-4)-, hydrogen, compd. with 1-butanamine (1:2:2)
Iodide	Plumbate(2-), tetraiodo-, (T-4)-, hydrogen, compd. with 1-cyclohexene-1-ethanamine (1:2:2)
Iodide	Plumbate(2-), tetraiodo-, (T-4)-, hydrogen, compd. with 1-decanamine (1:2:2)
Iodide	Plumbate(2-), tetraiodo-, (T-4)-, hydrogen, compd. with 1-dodecanamine (1:2:2)
Iodide	Plumbate(2-), tetraiodo-, (T-4)-, hydrogen, compd. with 1-heptanamine (1:2:2)
Iodide	Plumbate(2-), tetraiodo-, (T-4)-, hydrogen, compd. with 1-hexadecanamine (1:2:2)
Iodide	Plumbate(2-), tetraiodo-, (T-4)-, hydrogen, compd. with 1-hexanamine (1:2:2)
Iodide	Plumbate(2-), tetraiodo-, (T-4)-, hydrogen, compd. with 1-naphthalenemethanamine (1:2:2)
Iodide	Plumbate(2-), tetraiodo-, (T-4)-, hydrogen, compd. with 1-nonanamine (1:2:2)
Iodide	Plumbate(2-), tetraiodo-, (T-4)-, hydrogen, compd. with 1-octanamine (1:2:2)
Iodide	Plumbate(2-), tetraiodo-, (T-4)-, hydrogen, compd. with 1-pentanamine (1:2:2)
Iodide	Plumbate(2-), tetraiodo-, (T-4)-, hydrogen, compd. with 1-propanamine (1:2:2)
Iodide	Plumbate(2-), tetraiodo-, (T-4)-, hydrogen, compd. with 1-pyrenebutanamine (1:2:2)
Iodide	Plumbate(2-), tetraiodo-, (T-4)-, hydrogen, compd. with 1-tetradecanamine (1:2:2)
Iodide	Plumbate(2-), tetraiodo-, (T-4)-, hydrogen, compd. with 1-undecanamine (1:2:2)
Iodide	Plumbate(2-), tetraiodo-, (T-4)-, hydrogen, compd. with 1,3,5-hexatriene-1,6-diamine (1:2:1)
Iodide	Plumbate(2-), tetraiodo-, (T-4)-, hydrogen, compd. with 1,4-benzenediamine (1:2:1)
Iodide	Plumbate(2-), tetraiodo-, (T-4)-, hydrogen, compd. with 1,4-benzenedimethanamine (1:2:1)
Iodide	Plumbate(2-), tetraiodo-, (T-4)-, hydrogen, compd. with 1,4-butanediamine (1:2:1)
Iodide	Plumbate(2-), tetraiodo-, (T-4)-, hydrogen, compd. with 1,5-naphthalenediamine (1:2:1)
Iodide	Plumbate(2-), tetraiodo-, (T-4)-, hydrogen, compd. with 1,6-hexanediamine (1:2:1)

Iodide	Plumbate(2-), tetraiodo-, (T-4)-, hydrogen, compd. with 1,7-heptanediamine (1:2:1)
Iodide	Plumbate(2-), tetraiodo-, (T-4)-, hydrogen, compd. with 1,8-octanediamine (1:2:1)
Iodide	Plumbate(2-), tetraiodo-, (T-4)-, hydrogen, compd. with 1H-imidazole-5-ethanamine (1:2:1)
Iodide	Plumbate(2-), tetraiodo-, (T-4)-, hydrogen, compd. with 2-fluorobenzeneethanamine (1:2:2)
Iodide	Plumbate(2-), tetraiodo-, (T-4)-, hydrogen, compd. with 2-methyl-1-propanamine (1:2:2)
Iodide	Plumbate(2-), tetraiodo-, (T-4)-, hydrogen, compd. with 2-naphthaleneethanamine (1:2:2)
Iodide	Plumbate(2-), tetraiodo-, (T-4)-, hydrogen, compd. with 2-naphthalenemethanamine (1:2:2)
Iodide	Plumbate(2-), tetraiodo-, (T-4)-, hydrogen, compd. with 2-pyridineethanamine (1:2:1)
Iodide	Plumbate(2-), tetraiodo-, (T-4)-, hydrogen, compd. with 2-thiopheneethanamine (1:2:2)
Iodide	Plumbate(2-), tetraiodo-, (T-4)-, hydrogen, compd. with 2,2′-(2,5-cyclohexadiene-1,4-diylidene)bis[propanedinitrile] and 1-pyrenebutanamine (1:2:2:2)
Iodide	Plumbate(2-), tetraiodo-, (T-4)-, hydrogen, compd. with 2,2′-[1,2-ethanediylbis(oxy)]bis[ethanamine] (1:2:1)
Iodide	Plumbate(2-), tetraiodo-, (T-4)-, hydrogen, compd. with 2,3,4,5,6-pentafluorobenzeneethanamine (1:2:2)
Iodide	Plumbate(2-), tetraiodo-, (T-4)-, hydrogen, compd. with 3-fluorobenzeneethanamine (1:2:2)
Iodide	Plumbate(2-), tetraiodo-, (T-4)-, hydrogen, compd. with 3-methyl-1-butanamine (1:2:2)
Iodide	Plumbate(2-), tetraiodo-, (T-4)-, hydrogen, compd. with 3-piperidinemethanamine (1:2:1)
Iodide	Plumbate(2-), tetraiodo-, (T-4)-, hydrogen, compd. with 3-pyridineethanamine (1:2:1)
Iodide	Plumbate(2-), tetraiodo-, (T-4)-, hydrogen, compd. with 3-pyridinemethanamine (1:2:1)
Iodide	Plumbate(2-), tetraiodo-, (T-4)-, hydrogen, compd. with 4-(trifluoromethyl)benzeneethanamine (1:2:2)
Iodide	Plumbate(2-), tetraiodo-, (T-4)-, hydrogen, compd. with 4-bromo-α-methylbenzenemethanamine (1:2:2)

Iodide	Plumbate(2-), tetraiodo-, (T-4)-, hydrogen, compd. with 4-fluorobenzeneethanamine (1:2:1)
Iodide	Plumbate(2-), tetraiodo-, (T-4)-, hydrogen, compd. with 4-fluorobenzeneethanamine (1:2:2)
Iodide	Plumbate(2-), tetraiodo-, (T-4)-, hydrogen, compd. with 4-piperidinemethanamine (1:2:1)
Iodide	Plumbate(2-), tetraiodo-, (T-4)-, hydrogen, compd. with 4-pyridineethanamine (1:2:1)
Iodide	Plumbate(2-), tetraiodo-, (T-4)-, hydrogen, compd. with 4-pyridinemethanamine (1:2:1)
Iodide	Plumbate(2-), tetraiodo-, (T-4)-, hydrogen, compd. with 4,4-difluoropiperidine (1:2:2)
Iodide	Plumbate(2-), tetraiodo-, (T-4)-, hydrogen, compd. with (αR)-α-methylbenzenemethanamine (1:2:2)
Iodide	Plumbate(2-), tetraiodo-, (T-4)-, hydrogen, compd. with (αS)-α-methylbenzenemethanamine (1:2:2)
Iodide	Plumbate(2-), tetraiodo-, (T-4)-, hydrogen, compd. with benzeneethanamine (1:2:2)
Iodide	Plumbate(2-), tetraiodo-, (T-4)-, hydrogen, compd. with benzenemethanamine (1:2:2)
Iodide	Plumbate(2-), tetraiodo-, (T-4)-, hydrogen, compd. with cyclohexanamine (1:2:2)
Iodide	Plumbate(2-), tetraiodo-, (T-4)-, hydrogen, compd. with cyclohexaneethanamine (1:2:2)
Iodide	Plumbate(2-), tetraiodo-, (T-4)-, hydrogen, compd. with cyclohexanemethanamine (1:2:2)
Iodide	Plumbate(2-), tetraiodo-, (T-4)-, hydrogen, compd. with cyclopentanamine (1:2:2)
Iodide	Plumbate(2-), tetraiodo-, (T-4)-, hydrogen, compd. with ethanamine (1:2:2)
Iodide	Plumbate(2-), tetraiodo-, (T-4)-, hydrogen, compd. with guanidine and methanimidamide (1:2:1:1)
Iodide	Plumbate(2-), tetraiodo-, (T-4)-, hydrogen, compd. with β-methylbenzeneethanamine (1:2:2)
Iodide	Plumbate(3-), μ-iodohexaiododi-, hydrogen, compd. with 1-butanamine and methanamine (1:3:2:1)
Iodide	Plumbate(3-), μ-iodohexaiododi-, hydrogen, compd. with 1-butanamine and methanimidamide (1:3:2:1)
Iodide	Plumbate(3-), μ-iodohexaiododi-, hydrogen, compd. with 1-dodecanamine and methanamine (1:3:2:1)

Iodide	Plumbate(3-), μ-iodohexaiododi-, hydrogen, compd. with 1-hexanamine and methanamine (1:3:2:1)
Iodide	Plumbate(3-), μ-iodohexaiododi-, hydrogen, compd. with 1,4-benzenedimethanamine and methanimidamide (1:3:1:1)
Iodide	Plumbate(3-), μ-iodohexaiododi-, hydrogen, compd. with benzeneethanamine and methanamine (1:3:2:1)
Iodide	Plumbate(3-), μ-iodohexaiododi-, hydrogen, compd. with benzenemethanamine and methanamine (1:3:2:1)
Iodide	Plumbate(3-), μ-iodohexaiododi-, hydrogen, compd. with methanamine and 1-octanamine (1:3:1:2)
Iodide	Plumbate(3-), μ-iodohexaiododi-, hydrogen, compd. with methanamine and 1-tetradecanamine (1:3:1:2)
Iodide	Plumbate(3-), μ-iodohexaiododi-, hydrogen, compd. with methanamine and 1,3-propanediamine (1:3:1:1)
Iodide	Plumbate(3-), μ-iodohexaiododi-, hydrogen, compd. with methanamine and 1,8-octanediamine (1:3:1:1)
Iodide	Plumbate(3-), μ-iodohexaiododi-, hydrogen, compd. with methanamine and 2-methyl-1-propanamine (1:3:1:2)
Iodide	Plumbate(3-), μ-iodohexaiododi-, hydrogen, compd. with methanimidamide and 1-naphthalenemethanamine (1:3:1:2)
Iodide	Plumbate(3-), μ-iodohexaiododi-, hydrogen, compd. with N-methylmethanamine and 1-pentanamine (1:3:1:2)
Iodide	Plumbate(3-), μ-iodohexaiododi-, trihydrogen, compd. with methanamine and 2-thiophenemethanamine (1:1:2)
Iodide	Plumbate(4-), di-μ-iodooctaiodotri-, hydrogen, compd. with 1-butanamine and ethanamine (1:4:2:2)
Iodide	Plumbate(4-), di-μ-iodooctaiodotri-, hydrogen, compd. with 1-butanamine and methanamine (1:4:2:2)
Iodide	Plumbate(4-), di-μ-iodooctaiodotri-, hydrogen, compd. with 1-butanamine and methanimidamide (1:4:2:2)
Iodide	Plumbate(4-), di-μ-iodooctaiodotri-, hydrogen, compd. with benzenemethanamine and methanamine (1:4:2:2)

Iodide	Plumbate(4-), di-μ-iodooctaiodotri-, hydrogen, compd. with methanamine and 1,3-propanediamine (1:4:2:1)
Iodide	Plumbate(4-), di-μ-iodooctaiodotri-, hydrogen, compd. with methanamine and 1,8-octanediamine (1:4:2:1)
Iodide	Plumbate(4-), di-μ-iodooctaiodotri-, hydrogen, compd. with methanamine and 2-methyl-1-propanamine (1:4:2:2)
Iodide	Plumbate(4-), di-μ-iodooctaiodotri-, hydrogen, compd. with methanamine and 4-piperidinemethanamine (1:4:2:1)
Iodide	Plumbate(4-), hexa-μ-iodotetraiodotri-, hydrogen, compd. with 1-butanamine and methanamine (1:4:2:2)
Iodide	Plumbate(4-), hexa-μ-iodotetraiodotri-, hydrogen, compd. with 1-hexanamine and methanamine (1:4:2:2)
Iodide	Plumbate(4-), hexa-μ-iodotetraiodotri-, hydrogen, compd. with 1,4-benzenedimethanamine and methanimidamide (1:4:1:2)
Iodide	Plumbate(4-), hexa-μ-iodotetraiodotri-, hydrogen, compd. with 1,4-butanediamine and methanamine (2:8:1:6)
Iodide	Plumbate(4-), hexaiodo-, (OC-6-11)-, hydrogen, compd. with benzeneethanamine (1:4:4)
Iodide	Plumbate(4-), hexaiodo-, tetracesium, (OC-6-11)-
Iodide	Plumbate(4-), tri-μ-iododi-μ3-iodopentaiodotri-, hydrogen, compd. with 1-butanamine and methanamine (1:4:2:2)
Iodide	Plumbate(4-), tri-μ-iodoheptaiodotri-, hydrogen, compd. with benzeneethanamine and methanamine (1:4:2:2)
Iodide	Plumbate(5-), tri-μ-iododecaiodotetra-, hydrogen, compd. with 1-butanamine and methanamine (1:5:2:3)
Iodide	Plumbate(5-), tri-μ-iododecaiodotetra-, hydrogen, compd. with 1,4-benzenedimethanamine and methanimidamide (1:5:1:3)
Iodide	Plumbate(5-), tri-μ-iododecaiodotetra-, hydrogen, compd. with benzeneethanamine and methanamine (1:5:2:3)
Iodide	Plumbate(5-), tri-μ-iododecaiodotetra-, hydrogen, compd. with methanamine and 1,3-propanediamine (1:5:3:1)

Iodide	Plumbate(5-), tri-μ-iododecaiodotetra-, hydrogen, compd. with methanamine and 1,8-octanediamine (1:5:3:1)
Iodide	Plumbate(5-), tri-μ-iododecaiodotetra-, hydrogen, compd. with methanamine and 2-methyl-1-propanamine (1:5:3:2)
Iodide	Plumbate(5-), tri-μ-iododecaiodotetra-, hydrogen, compd. with methanamine and 3-pyridinemethanamine (1:5:3:1)
Iodide	Plumbate(5-), tri-μ-iododecaiodotetra-, hydrogen, compd. with methanamine and 4-pyridinemethanamine (1:5:3:1)
Iodide	Plumbate(6-), tetra-μ-iodododecaiodopenta-, hydrogen, compd. with 1-butanamine and methanamine (1:6:2:4)
Iodide	Plumbate(6-), tetra-μ-iodododecaiodopenta-, hydrogen, compd. with 4-fluorobenzeneethanamine and methanamine (1:6:2:4)
Iodide	Plumbate(6-), tetra-μ-iodododecaiodopenta-, hydrogen, compd. with benzeneethanamine and methanamine (1:6:2:4)
Iodide	Plumbate(6-), tetra-μ-iodododecaiodopenta-, hydrogen, compd. with methanamine and 1-octanamine (1:6:4:2)
Iodide	Plumbate(6-), tetra-μ-iodododecaiodopenta-, hydrogen, compd. with methanamine and 1-propanamine (1:6:4:2)
Iodide	Plumbate(6-), tetra-μ-iodododecaiodopenta-, hydrogen, compd. with methanamine and 2-methyl-1-propanamine (1:6:4:2)
Iodide	Stannate(2-), tetraiodo-, (T-4)-, dihydrogen, compd. with 1,4-butanediamine (1:1)
Iodide	Stannate(2-), tetraiodo-, (T-4)-, dihydrogen, compd. with 4-fluorobenzeneethanamine (1:2)
Iodide	Stannate(2-), tetraiodo-, (T-4)-, hydrogen (T-4)-tetraiodogermanate(2-), compd. with benzeneethanamine (1:4:1:2)
Iodide	Stannate(2-), tetraiodo-, (T-4)-, hydrogen (T-4)-tetraiodogermanate(2-), compd. with benzeneethanamine (1:8:3:2)
Iodide	Stannate(2-), tetraiodo-, (T-4)-, hydrogen (T-4)-tetraiodogermanate(2-), compd. with benzeneethanamine (1:16:7:2)
Iodide	Stannate(2-), tetraiodo-, (T-4)-, hydrogen, compd. with 1-butanamine (1:2:2)
Iodide	Stannate(2-), tetraiodo-, (T-4)-, hydrogen, compd. with 1-hexanamine (1:2:2)

Iodide	Stannate(2-), tetraiodo-, (T-4)-, hydrogen, compd. with 1-octanamine (1:2:2)
Iodide	Stannate(2-), tetraiodo-, (T-4)-, hydrogen, compd. with 2-thiopheneethanamine (1:2:2)
Iodide	β-Alanine, (T-4)-tetraiodoplumbate(2-) (2:1)
Mixed halide	Cesium lead bromide iodide (CsPb2BrI4)
Mixed halide	Cesium lead chloride iodide (Cs2PbCl2I2)
Mixed halide	Plumbate(2-), bromotrichloro-, (T-4)-, dihydrogen, compd. with benzeneethanamine (1:2)
Mixed halide	Plumbate(2-), bromotrichloro-, (T-4)-, hydrogen, compd. with benzenemethanamine (1:2:2)
Mixed halide	Plumbate(2-), bromotriiodo-, (T-4)-, dihydrogen, compd. with benzeneethanamine (1:2)
Mixed halide	Plumbate(2-), chlorotriiodo-, (T-4)-, hydrogen, compd. with methanamine (1:2:2)
Mixed halide	Plumbate(2-), dibromodichloro-, (T-4)-, dihydrogen, compd. with benzeneethanamine (1:2)
Mixed halide	Plumbate(2-), dibromodichloro-, (T-4)-, hydrogen, compd. with benzenemethanamine (1:2:2)
Mixed halide	Plumbate(2-), dibromodiiodo-, (T-4)-, dihydrogen, compd. with benzeneethanamine (1:2)

Table S9: List of Clays with their structural groups	
2:1 (TOT) swelling clays – smectite group	Montmorillonite
2:1 (TOT) swelling clays – smectite group	Hectorite
2:1 (TOT) swelling clays – smectite group	Nontronite
2:1 (TOT) swelling clays – smectite group	Saponite
2:1 (TOT) swelling clays – smectite group	Saponite
2:1 (TOT) swelling clays – smectite group	Laponite S 482
2:1 (TOT) swelling clays – smectite group	B 38
2:1 (TOT) limited-swelling clays	Vermiculite
1:1 (TO) kaolin group	Halloysite
1:1 (TO) kaolin group	Kaolinite
1:1 (TO) kaolin group	Halloysite
1:1 (TO) kaolin group	Endellite
1:1 (TO) kaolin group	Nacrite-2M2
1:1 (TO) kaolin group	Nacrite
1:1 (TO) kaolin group	Dickite

Fibrous / ribbon silicates	Palygorskite ([Mg(Al0.5-1Fe0-0.5)]Si4(OH)O10.4H2O)
Fibrous / ribbon silicates	Sepiolite
2:1 (TOT) non-swelling micas	Illite ([Al1.75(Fe0-1Mg0-1)0.25]K0.75(Si3.5Al0.5)[(OH)0.5-1F0-0.5]2O10)
2:1 (TOT) non-swelling micas	Sericite
2:1 (TOT) neutral layered silicates	Pyrophyllite (AlH(SiO3)2)
2:1 (TOT) neutral layered silicates	Ferripyrophyllite (FeH(SiO3)2)
Others	Sudoite (Al(Al0-1Fe0-1)3Mg2Si3(OH)8O10)
Others	Sodium-rectorite
Others	Pennantite
Others	Lizardite (Mg3H2(SiO4)2.H2O)
Others	Donbassite
Others	Cookeite (Li[Al5O2(SiO4)3].4H2O)
Others	Cloisite 30B
Others	Borocookeite (Al4Li(Si3B)(OH)8O10)

Table S10: Substance names grouped for 2D transition metal oxides

**Metal-based oxide**	**Substance name**
Mo–based Oxide	Molybdenum oxide (MoO3)
Mo–based Oxide	Molybdenum dioxide
Mo–based Oxide	Molybdenum oxide
Mo–based Oxide	Molybdenum sodium oxide (Mo2Na0.9O4)
Mo–based Oxide	Molybdenum sodium oxide
Mo–based Oxide	Ammonium molybdenum oxide
V-based oxide	Vanadium oxide (V2O5)
V-based oxide	Vanadium oxide
V-based oxide	Vanadium oxide (V5O12)
V-based oxide	Vanadium oxide (V2O5), hydrate (1:1)
V-based oxide	Lithium vanadium oxide
V-based oxide	Lithium vanadium oxide (LiV2O5)
V-based oxide	Lithium vanadium oxide, hydrate
V-based oxide	Sodium vanadium oxide
V-based oxide	Sodium vanadium oxide (NaV6O15)
V-based oxide	Potassium vanadium oxide
V-based oxide	Potassium vanadium oxide (KV2O5)
V-based oxide	Calcium vanadium oxide
V-based oxide	Calcium vanadium oxide (CaV4O9)
V-based oxide	Ammonium vanadium oxide ((NH4)VO3)

W-based oxide	Tungsten oxide (WO3)
W-based oxide	Tungsten oxide (W10O29)
W-based oxide	Sodium tungsten oxide
W-based oxide	Sodium tungsten oxide (Na2WO4)

Table S11: Substance names grouped for non-Mxene 2D transition metal nitrides, carbides & borides

TM-N/C/B	Substance names
Nitride	Titanium mononitride
Nitride	Tungsten nitride
Nitride	Molybdenum nitride
Nitride	Molybdenum nitride (Mo2N)
Nitride	Zirconium nitride (ZrN)
Nitride	Vanadium nitride (VN)
Nitride	Tungsten nitride (W2N3)
Nitride	Tungsten nitride (W2N)
Nitride	Titanium nitride (TiN0.75)
Nitride	Tantalum nitride (TaN)
Nitride	Ruthenium nitride (RuN2)
Nitride	Niobium nitride (NbN2)
Nitride	Niobium nitride (NbN)
Nitride	Molybdenum nitride (MoN2)
Nitride	Molybdenum nitride (Mo5N6)
Nitride	Hafnium nitride (HfN)
Nitride	Vanadium nitride (VN2)
Nitride	Vanadium nitride
Nitride	Tungsten nitride (WN2)
Nitride	Tungsten nitride (WN)
Nitride	Tungsten nitride (W5N6)
Nitride	Tungsten nitride (W4N2), radical ion(1-)
Nitride	Tungsten nitride (W2N2)
Nitride	Titanium nitride (TiN2)
Nitride	Titanium nitride (Ti2N)
Nitride	Technetium nitride (99TcN2)
Nitride	Tantalum nitride (TaN2)

Nitride	Rhenium nitride (ReN2)
Nitride	Osmium nitride (OsN2)
Nitride	Niobium nitride (Nb4N5)
Nitride	Niobium nitride (Nb3N4)
Nitride	Niobium nitride (Nb2N3)
Nitride	Niobium nitride
Nitride	Nickel nitride
Nitride	Molybdenum nitride (MoN)
Nitride	Manganese nitride (MnN2)
Nitride	Iron nitride (FeN2)
Nitride	Iron nitride
Nitride	Hafnium nitride (HfN2)
Nitride	Hafnium nitride (HfN1.5)
Nitride	Hafnium hydride nitride (Hf2HN3)
Nitride	Hafnium fluoride nitride (Hf2FN3)
Nitride	Hafnium chloride nitride (HfClN)
Nitride	Copper nitride (Cu2N3)
Nitride	Copper nitride
Nitride	Copper hydride nitride (Cu2HN3)
Nitride	Copper fluoride nitride (Cu2FN3)
Nitride	Copper chloride nitride (Cu2ClN3)
Nitride	Cobalt nitride (CoN1.5)
Nitride	Cobalt nitride
Nitride	Chromium nitride (CrN2)
Carbide	Titanium carbide (TiC)
Carbide	Vanadium carbide (V2C)
Carbide	Niobium carbide (Nb2C)
Carbide	Tungsten carbide (WC)
Carbide	Tantalum carbide (TaC)
Carbide	Tantalum carbide (Ta4C3)
Carbide	Niobium carbide (NbC)
Carbide	Molybdenum carbide (Mo2C)
Carbide	Vanadium carbide (VC)
Carbide	Hafnium carbide (HfC)
Carbide	Chromium vanadium carbide (Cr1.5V1.5C2)
Carbide	Chromium carbide (CrC)
Carbide	Chromium carbide (Cr2C)
Carbide	Vanadium carbide (V3C2)
Carbide	Tungsten carbide (W2C3)
Carbide	Tungsten carbide (W2C)
Carbide	Tungsten carbide (W0.2C0.8)

Carbide	Titanium carbide (TiC2)
Carbide	Titanium carbide (TiC0.75)
Carbide	Titanium carbide (Ti2C3)
Carbide	Tantalum carbide (Ta2C)
Carbide	Scandium carbide
Carbide	Ruthenium carbide
Carbide	Rhenium carbide (ReC)
Carbide	Rhenium carbide (Re2C)
Carbide	Nickel carbide
Carbide	Molybdenum carbide
Carbide	Molybdenum carbide (MoC)
Carbide	Manganese carbide (MnC)
Carbide	Manganese carbide
Carbide	Iron carbide
Carbide	Copper carbide
Carbide	Cobalt carbide
Carbide	Chromium carbide (CrC2)
Carbide	Chromium carbide (Cr3C2)
Carbide	Chromium carbide
Boride	Chromium boride (CrB)
Boride	Titanium diboride
Boride	Vanadium diboride
Boride	Niobium boride (NbB2)
Boride	Molybdenum boride (MoB2)
Boride	Molybdenum boride (MoB)
Boride	Iron boride (FeB2)
Boride	Zirconium boride (ZrB)
Boride	Titanium boride (TiB)
Boride	Molybdenum boride
Boride	Manganese boride (MnB)
Boride	Hafnium boride (HfB2)
Boride	Hafnium boride (HfB)
Boride	Vanadium boride (VB)
Boride	Tungsten boride (WB2)
Boride	Titanium boride
Boride	Tantalum boride (TaB2)
Boride	Tantalum boride (TaB)
Boride	Scandium boride (ScB2)
Boride	Ruthenium boride (RuB2)
Boride	Osmium boride (OsB2)
Boride	Niobium boride (NbB)

Boride	Molybdenum boride (Mo2B)
Boride	Manganese boride (MnB2)
Boride	Iron boride (FeB)
Boride	Cobalt boride (CoB2)
Boride	Chromium boride (CrB2)
Boride	Zirconium diboride
Boride	Zirconium boride (ZrB1.5)
Boride	Zirconium boride (Zr2B)
Boride	Zirconium boride
Boride	Vanadium boride (VB12)
Boride	Vanadium boride (V3B4)
Boride	Vanadium boride (V2B6)
Boride	Vanadium boride (V2B2)
Boride	Tungsten boride (WB)
Boride	Tungsten boride (W2B3)
Boride	Tungsten boride (W2B2)
Boride	Titanium boride (TiB12)
Boride	Technetium boride (TcB2)
Boride	Tantalum boride (Ta3B4)
Boride	Tantalum boride (Ta2B3)
Boride	Tantalum boride (Ta2B)
Boride	Tantalum boride
Boride	Scandium boride (ScB)
Boride	Rhenium boride (ReB2)
Boride	Niobium boride (Nb5B)
Boride	Niobium boride (Nb3B4)
Boride	Niobium boride (Nb2B3)
Boride	Niobium boride (Nb2B)
Boride	Niobium boride
Boride	Nickel boride (NiB2)
Boride	Nickel boride (NiB)
Boride	Nickel boride (Ni2B6)
Boride	Molybdenum boride (MoB2.2)
Boride	Molybdenum boride (Mo2B5)
Boride	Molybdenum boride (Mo2B3)
Boride	Manganese boride (MnB12)
Boride	Manganese boride (Mn3B4)
Boride	Manganese boride (Mn2B2)
Boride	Manganese boride
Boride	Iron boride (FeB12)
Boride	Iron boride (Fe2B6)

Boride	Iron boride (Fe2B)
Boride	Hafnium boride (Hf2B3)
Boride	Hafnium boride (Hf2B)
Boride	Gold boride (Au2B)
Boride	Copper boride (CuB2)
Boride	Cobalt boride (CoB)
Boride	Cobalt boride (Co2B6)
Boride	Chromium manganese boride (CrMnB2)
Boride	Chromium iron boride (CrFeB2)
Boride	Chromium boride (CrB3)
Boride	Chromium boride (CrB12)
Boride	Chromium boride
